# Regulatory T cells in homeostasis and disease: molecular mechanisms and therapeutic potential

**DOI:** 10.1038/s41392-025-02326-4

**Published:** 2025-10-14

**Authors:** Lingling Wang, Ying Liang, Chunxia Zhao, Peijun Ma, Shulin Zeng, Dongen Ju, Minggao Zhao, Min Yu, Yun Shi

**Affiliations:** 1 Wuchang Hospital Affiliated to Wuhan University of Science and Technology, Wuhan Wuchang Hospital, Wuhan, China; 2 Precision Pharmacy & Drug Development Center, Department of Pharmacy, Tangdu Hospital, Fourth Military Medical University, Xi’an, Shaanxi China; 3 Department of Clinical Laboratory, Shanghai Mental Health Center, Shanghai Jiao Tong University School of Medicine, Shanghai, China; 4 Department of Urology, Xijing Hospital, Fourth Military Medical University, Xi’an, Shaanxi China; 5 Key Laboratory of Metabolism and Molecular Medicine, Ministry of Education, Department of Biochemistry and Molecular Biology, School of Basic Medical Sciences, Fudan University, Shanghai, China; 6 Department of Biochemistry and Molecular Biology, School of Basic Medical Sciences, Fudan University, Shanghai, China; 7 Department of Immunology & Theranostics, Arthur Riggs Diabetes & Metabolism Research Institute, Beckman Research Institute of the City of Hope, Duarte, CA USA

**Keywords:** Drug development, Outcomes research

## Abstract

Regulatory T cells (Treg cells or Tregs), a subset of CD4⁺ T cells with immunosuppressive properties, are essential for immune homeostasis and self-tolerance. Characterized by their immunosuppressive capabilities and reliance on the transcription factor Foxp3 (Forkhead box protein P3), Tregs employ multiple mechanisms, including cytokine secretion, metabolic control, and cell contact inhibition, to restrain excessive immune activation to prevent autoimmunity while maintaining tissue repair processes. However, dysregulation in their frequency or function—whether deficiency or hyperactivity—is implicated in diverse pathologies, spanning autoimmune disorders, cancer progression, transplant rejection, and emerging associations with neurological and cardiovascular diseases. Thus, Treg-targeted strategies represent a promising approach for restoring immune balance under various conditions. This review synthesizes current knowledge on Treg biology, from their discovery and definition of markers to their new regulatory mechanisms. We further explore the roles of Tregs across diseases, emphasizing their context-dependent therapeutic potential. Strategies to deplete or inhibit Tregs in cancer immunotherapy contrast with approaches to expand or stabilize their function in autoimmunity and transplantation. However, challenges persist, including achieving tissue-specific targeting, ensuring the functional stability of engineered Tregs, and minimizing off-target effects. By integrating mechanistic insights with translational innovations, this review provides a roadmap for advancing Treg-based therapies, ultimately aiming to restore immune equilibrium in a disease-specific manner.

## Introduction

Immune homeostasis refers to the immune system’s ability to maintain balance and stability, ensuring proper function under normal physiological conditions and during immune responses.^1^ Foxp3, the key transcription factor of Treg cells, governs their function by modulating the expression of specific genes, either activating or repressing targets.^2–4^ As essential regulators of immune balance, Foxp3⁺ Tregs contribute to self-tolerance and immune homeostasis.^5,6^ An imbalance in Treg activity or numbers can have significant consequences: excessive Treg activity or abundance may result in immunodeficiency, chronic infections, and cancer, whereas insufficient Treg activity or numbers may induce autoimmunity, immunopathology, and compromised pathogen-specific immune responses.^7^ Notably, Tregs exhibit exceptional plasticity, enabling them to adapt to their microenvironment and enhancing their suppressive functions.^8^ However, under inflammatory conditions, these cells may differentiate into effector cells, such as Th1 or Th17 cells.^9^ Tregs not only suppress T cells but also modulate B cells, natural killer (NK) cells, and nonlymphoid cells, such as those in adipose tissue.^1,10,11^ Exploring Treg metabolism can provide insights into targeted therapeutic strategies.

Treg cell targeting has become a promising approach for modulating immune responses, restoring balance, and treating various diseases. These cells are crucial for preserving immune stability by modulating excessive immune responses and safeguarding against autoimmunity. Given their central role in immune regulation, Treg-targeted therapies have been widely applied across multiple diseases, notably in cancer immunotherapy, where Tregs serve as key immunosuppressive factors by inhibiting antitumor immunity, promoting tumor progression, and reducing therapeutic efficacy.^12–14^ Current targeting strategies include monoclonal antibodies,^15^ small-molecule inhibitors,^16^ bispecific antibodies,^17^ and cell-based therapies, such as engineered TCR-Tregs and CAR-Tregs.^18^ While these approaches show significant promise, they also face challenges, such as achieving selective targeting of tumor-infiltrating Tregs (TI-Tregs), minimizing the risk of autoimmunity, and addressing Treg heterogeneity and plasticity. Among these strategies, cell-based therapies have gained particular attention, with engineered CAR-Tregs demonstrating remarkable efficacy in disease models, including transplantation,^19,20^ autoimmune disorders,^21^ and graft-versus-host disease (GVHD).^22^ Clinical trials involving patients with type 1 diabetes^23,24^ and GVHD^16,25,26^ have provided preliminary evidence supporting the feasibility, safety, and efficacy of Treg-based therapies. Moving forward, deepening our understanding of Treg biology, including their metabolic states and epigenetic regulation, optimizing targeting strategies, and developing precise regulatory tools will pave the way for expanding the clinical applications of Treg-targeted therapies across a wide range of disease settings. This review explores the functions and characteristics of Treg cells, as well as their roles in various diseases and therapeutic approaches, including cancer, neurodegenerative disorders, autoinflammatory and autoimmune diseases, and transplantation.

## The discovery history of Treg cells

**Fig. 1:**
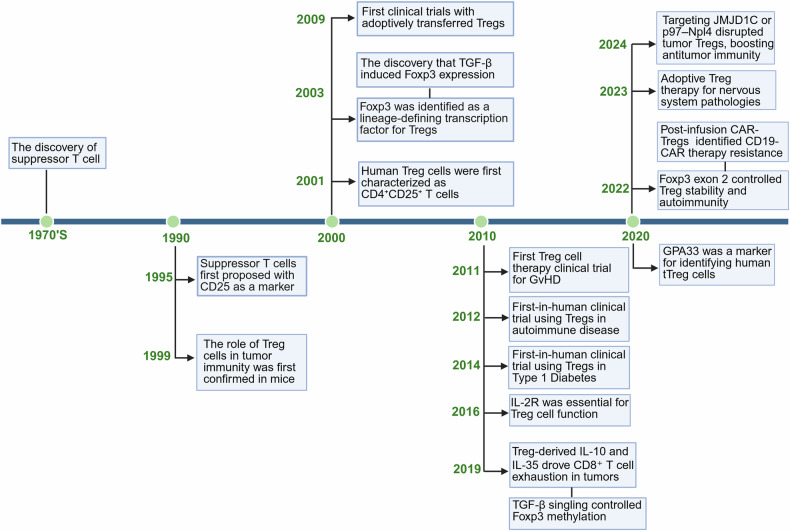
History of Treg cells. Timeline showing representative events of key biological discoveries, clinical trials, and key mechanism discovery for Treg cell immunoregulatory targets of anticancer therapeutics. The blue boxes denote seminal breakthroughs. This figure was created with Biorender.com

In the early 1970s, researchers first identified a subset of T cells with immunosuppressive properties.^27–29^ Specifically, in 1995, Sakaguchi et al. reported that CD25 (IL-2 receptor subunit-α) was a phenotypic marker of mouse suppressive CD4^+^ T cells and proposed the concept of Treg cells.^30^ Later, this type of suppressive T cell was also identified in the human CD4^+^CD25^high^ T cell population.^31–33^ In 2003, Foxp3, a forkhead/winged-helix transcription factor specifically expressed in Tregs, was subsequently identified as a lineage-defining factor in mouse^2,3,34^ and human Tregs,^35,36^ thus establishing CD4^+^CD25^+^Foxp3^+^ cells as a defining feature of Tregs. In the same year, scientists reported that transforming growth factor (TGF)-β can induce naive CD4^+^ cells to differentiate into Treg cells and inhibit the activity of conventional T cells in vitro.^37^ Since 2010, researchers have explored the characteristics of Tregs and the role of Foxp3 in treating diseases. Extensive preclinical studies have demonstrated the critical role of Tregs in preventing GVHD,^25,26,38^ autoimmune diseases,^21,39^ transplant rejection,^19,20,40^ tumors,^41,42^ and even neurodegenerative diseases.^43,44^ These studies have yielded positive results and provided a theoretical basis for the therapeutic application of Tregs. The first clinical trial of adoptive transfer of Tregs in patients with GVHD was published in 2009.^45^ In 2011 and 2012, researchers conducted human clinical studies on the adoptive transfer of Treg cells in patients with stem cell transplantation^46^ and Crohn’s disease. The first clinical use of Tregs for type 1 diabetes treatment was reported in 2014, which demonstrated that ex vivo expanded autologous Tregs were safe for patients.^47^ Additionally, research has indicated that Treg depletion or transplantation of in vitro-depleted Treg suspensions into T cell-deficient mice effectively suppresses tumor growth.^48^ More recently, Deepali et al. reported that IL-10 and IL-35 from Treg cells in the tumor microenvironment (TME) promote CD8^+^ T cell exhaustion, and that their deletion reduces tumor growth.^49^ Targeting JMJD1C^50^ or p97–Npl4^51^ disrupts tumor Treg cells, increasing antitumor immunity. Advances in cell encapsulation and microneedle technologies offer novel delivery systems for Tregs, protecting them from immune rejection and enhancing their efficacy (Fig. 1).^52^

## Characterization of Treg cells

### Classification of Treg cells

Treg cells exhibit substantial heterogeneity and can be classified according to their origin, activation status, tissue-specific localization, and recent insights from single-cell sequencing technologies. Traditionally, Tregs are categorized based on their developmental origin into three main Treg populations: (1) the majority of Foxp3^+^ Tregs are generated in the thymus and are referred to as thymus-derived Tregs (tTregs); (2) in the periphery, a certain proportion of Tregs can be derived from Foxp3^−^ T conventional (Tconv) cells, and this population is known as peripherally derived Tregs (pTregs); and (3) when Tconv cells are stimulated in vitro with TGF-β, they differentiate into Foxp3⁺ T cells, known as in vitro-induced Tregs (iTregs).^53^ Early studies identified Helios^54^ and Neuropilin-1 (NRP1)^55,56^ as tTreg cell markers, but they were subsequently identified in pTreg cells.^55^ Recently, GPA33 was shown to be a reliable marker for human tTregs.^57^ tTregs exhibit a stable regulatory phenotype characterized by full demethylation of the Treg-specific demethylated region (TSDR), ensuring long-term immunosuppressive function. In contrast, pTregs show partial TSDR demethylation, potentially making them less stable and more susceptible to conversion into effector T cells under certain conditions. Moreover, iTregs retain a fully methylated TSDR, indicating their transient and less stable regulatory nature.^58,59^ In addition to origin-based subsets, Tregs can also be distinguished based on their activation status. Human naive or resting Tregs (rTreg) typically express CD45RA and relatively low levels of Foxp3 (CD45RA⁺Foxp3^lo^). Upon activation, they transition to an activated state characterized by CD45RO expression along with elevated levels of Foxp3, inducible co-stimulator (ICOS), and cytotoxic T lymphocyte antigen-4 (CTLA-4), and acquire enhanced suppressive functions.^60–63^ Similarly, in mice, naive (resting) Tregs possess a CD44^lo^CD62L⁺ phenotype and primarily reside in lymphoid tissues. Activated Tregs (aTregs), which are characterized by CD44^hi^CD62L⁻ along with high ICOS and CTLA-4 expression, migrate to sites of inflammation to exert potent immunosuppressive effects.^64,65^ Recent research has further revealed the existence of specialized tissue-specific Treg subsets adapted uniquely to their anatomical niches. Adipose tissue-resident Tregs express distinct markers, such as peroxisome proliferator-activated receptor (PPARγ) and ST2 (the receptor for IL-33), which play critical roles in regulating adipose tissue inflammation and metabolic homeostasis.^66,67^ Intestine-resident Tregs frequently coexpress RAR-related orphan receptor γt (RORγt), which is essential for maintaining intestinal mucosal tolerance.^68,69^ Tumor-infiltrating Tregs typically express high levels of ICOS, PD-1, and CCR8, significantly contributing to immune evasion and tumor progression.^70,71^ Additionally, skin-resident Tregs express homing markers such as cutaneous lymphocyte antigen (CLA) and CCR4,^70,72^ whereas central nervous system (CNS)-resident Tregs produce amphiregulin and express CD103, supporting tissue repair functions.^73,74^

Recent advances in single-cell RNA sequencing (scRNA-seq) have provided new insights into the classification of novel Treg subsets with high resolution.^75–78^ Using scRNA-seq, six Treg clusters have been identified in healthy peripheral blood,^76^ whereas other studies suggest further subdivision into nine clusters.^77^ Furthermore, scRNA-seq analysis revealed that T cell receptor (TCR) signal strength does not affect the proportions of resting/activated Tregs. However, TCR signals unexpectedly shape the diverse functional states of activated Tregs.^75^ Additionally, Treg cells exhibit transcriptional flexibility, adapting to different tissue environments while preserving core expression programs across homeostasis, disease, and species.^76^ Trajectory analyses revealed two Treg differentiation paths (I/II) with distinct phenotypic and functional programs, culminating in the Foxp3^hi^ and MKI67^hi^ subsets, driven by transcription factors such as Foxp3 and SUB1, respectively. These findings provide a single-cell atlas for exploring Treg complexity in health and disease.^77^ These single-cell insights emphasize the complexity of Treg biology, underscoring the diversity of Treg functional states, which were previously unrecognized via traditional methods.

### Markers of Treg cells

Treg-expressed molecules are categorized as intracellular (Foxp3, Helios) and extracellular (CD304, CD25, CD39, CD73, CD62L, CD103, CD134, FR4, CD127⁻, CD152, CD279, GITR, CD223, TIGIT) (Table 1). Foxp3 and Helios are key intracellular markers of Tregs. Foxp3, a crucial transcription factor, drives the differentiation of naive CD4⁺ T cells into Tregs and is essential for their suppressive function.^79,80^ Helios, a member of the Ikaros zinc finger protein family, is expressed predominantly in tTregs.^81,82^ CD304 (NRP-1), a transmembrane protein, is highly expressed on tTregs.^55,56^ Therefore, Helios and NRP1 are often used to separate tTreg cells from pTreg cells in certain physiological settings. CD25, the alpha chain of the IL-2 receptor, plays a key role in the activation and proliferation of immune cells. It is widely used to identify Treg cells and has therapeutic significance in both immunosuppressive and activating settings.^15,83^ CD39 and CD73, two ectonucleases abundantly expressed on Tregs, serve as key markers for this T cell subset.^84,85^ These ectonucleases are essential for adenosine production in Treg cells, as they work together to generate extracellular adenosine through ATP hydrolysis, promoting an immunosuppressive microenvironment.^84,86^ CD62L, a selectin molecule, facilitates the homing of immune cells to lymphoid tissues.^87,88^ Treg cells support their migration to secondary lymphoid organs.^89^ Like CD62L, CD103 (αEβ7 integrin) is an adhesion molecule essential for mucosal immunity that facilitates lymphocyte adhesion, graft rejection, and gut-homing effector cell generation. It plays a crucial role in retaining Tregs at inflammatory sites.^90^ CD134 (OX40) is a costimulatory receptor that is transiently expressed on effector T cells after TCR activation and promotes their proliferation and memory formation. Additionally, OX40 costimulates Tregs, reducing their suppressive function and Foxp3 expression.^91,92^ The ability of OX40 to activate T cells while limiting Treg suppression makes OX40 a key therapeutic target in cancer and autoimmunity. Folic acid receptor 4 (FR4) is expressed at high levels in TGF-β-induced Tregs and tTregs and plays a crucial role in sustaining Treg proliferation.^93,94^ CD127^low^, along with Foxp3 and CD25, serve as identification markers for Tregs, which are characterized as Foxp3^+^CD25^+^CD127^low^.^95,96^ CD152 (CTLA-4), CD279 (PD-1) and GITR are immune checkpoint molecules highly expressed on Tregs, where they engage their respective ligands on effector T cells to mediate immunosuppression. These two molecules help Tregs maintain tolerance by preventing overactive immune responses but also enable tumors to evade immunity through enhanced Treg-mediated suppression.^97–100^ CD223 (LAG-3) and TIGIT are immune checkpoint molecules, such as PD-1 and CTLA-4, but they have distinct functions, particularly in different tissue sites where they regulate various aspects of immunity. Many patients continue to show limited response to therapies targeting CTLA-4 and PD-1, driving the exploration of LAG-3 and TIGIT as the next generation of coinhibitory receptor targets in clinical trials.^101,102^ The specific functions of these inhibitory checkpoint molecules are discussed in detail in the section on immunotherapy strategies targeting Tregs in human diseases.

**Table 1:** Markers of Treg cells

Markers	Alias	Distributed	Characteristic	Function
Intracellular Markers				
Foxp3	Forkhead box P3	Treg cells	Transcription factors	Play a suppressive role in the immune system.^5,6,79,80,536^
Helios		Treg cells	Transcription factors	Helios is critical for maintaining Treg identity, repressing their ability to express effector cytokines, involved in the regulation of lymphocyte development.^81,82^
Extracellular Markers				
CD304	NRP1	Tregs, dendritic cells, endothelial cells, neuronal cells, epithelial and tumor cells	Transmembrane glycoprotein	NRP1 is a multifunctional receptor that plays key roles in various biological processes, particularly in immune regulation, angiogenesis, and neural development.^55,56,537^
CD25	IL-2Rα/Ly-43/P55/Tac	Activated T and B cells, thymocyte subsets, pre-B cells, Treg cells	Transmembrane glycoprotein	Marker of T cell activation, one of biomarkers to track disease progression and to indicate outcome for clinical disorders; a therapeutic target.^15,83,538^
CD39	NTPDase 1	B cells, dendritic cells, T cell subsets, Treg cells, memory T cells	Transmembrane glycoprotein	Degrade ATP to AMP, hydrolysis of extracellular ATP is crucial in terms of their immunosuppressive functions.^84,86^
CD73	5’Nucleotidase	Myeloid cells, T cells	Transmembrane glycoprotein	Convert extracellular ATP to immunosuppressive adenosine in concert with CD39 in normal tissues to limit excessive immune response.^84–86^
CD62L	LECAM-1	B and T cells, NK cells, tumor cells monocytes, granulocytes,	Transmembrane glycoprotein	Regulates leukocyte migration to inflammation sites and lymphocyte recirculation between blood and lymphoid tissues.^88,89,539^
CD103	ITGAE	T cells	Transmembrane glycoprotein	Mediates cell adhesion, migration, and lymphocyte homing of cell through interaction with E-cadherin.^90^
FR4	FRα	Treg cells	Transmembrane glycoprotein	Bind to CD4 and CD25 to distinguish Tregs from other types of T cells; significantly downregulates the expression of Tregs and improves the effect of chemotherapy and PD-1/PD-L1 inhibitors.^93,94^
CD127^low^	IL-7Rα	Naive CD4^+^ T cells, immature B cell, T cells, a subset of monocytes, a subset of CD34^+^ cells	Transmembrane glycoprotein	It can serve as a marker of Treg and conventional T cell differentiation.^95,540^
CD152	CTLA-4/Ly-56	Activated T cells, B cells	Transmembrane glycoprotein	Functions as an immune checkpoint receptor and downregulates immune responses.^97–99^
GITR	TNFRSF18/AITR	Activated CD4^+^ and CD8^+^ T cells, NK cells, myeloid cells, Treg cells	Transmembrane glycoprotein	Enhance T cell activation, proliferation, and cytokine production, and eliminate the inhibitory function of Tregs. Activation of GITR in vivo leads to the development of autoimmune diseases and restores suppressed immune responses.^100,418,541^
CD223	LAG-3	NK cells, B cells, activated and exhausted CD4^+^ and CD8^+^ T cells, Treg cells, dendritic cells	Transmembrane glycoprotein	Delivering inhibitory signals that regulate immune cell homeostasis, T cell activation, proliferation, cytokine production, cytolytic activity and other functions.^402^
TIGIT	Vstm3/VSIG9	T cells, B cells and NK cells	Transmembrane glycoprotein	TIGIT is a coinhibitory receptor expressed on T cells and natural killer (NK) cells, playing a key role in immune regulation by interacting with its ligands, such as CD155 (PVR) and CD112 (Nectin-2).^542,543^

### Stability of Tregs

**Fig. 2:**
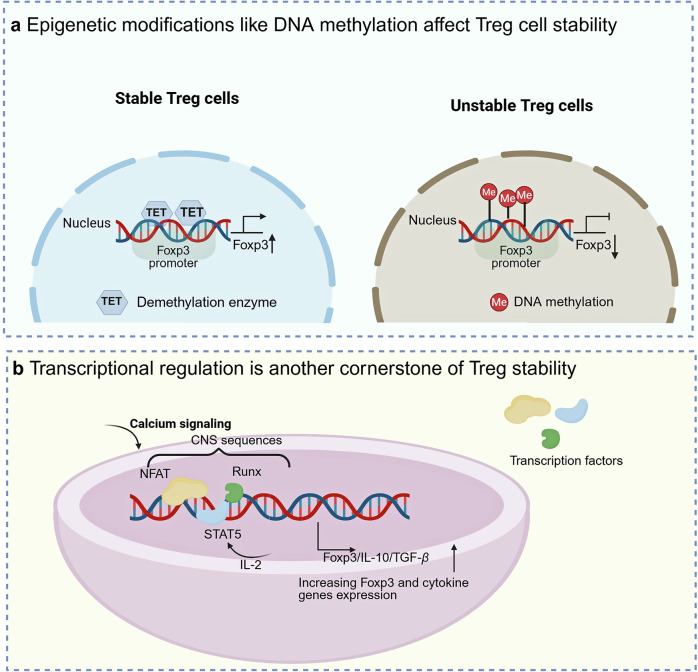
Regulatory mechanisms for Treg cell stability. **a** Treg cell stability can be influenced by epigenetic modifications such as DNA methylation. For instance, the promoter of *Foxp3* is usually demethylated to activate *Foxp3* expression. **b** The expression of *Foxp3* and other genes specific to Treg cells, such as TGF-β and IL-10, is precisely regulated by crucial transcription factors, including NFAT, STAT5, and Runx. This figure was created with Biorender.com

Treg cell stability refers to the capacity of regulatory T cells to sustain Foxp3 expression under inflammatory stress while preventing their conversion into pro-inflammatory effector cells. In simpler terms, it refers to their capacity to remain as Foxp3^+^ Treg cells and avoid transitioning into Foxp3⁻ ex-Treg cells.^103–106^ This stability is a complex phenomenon governed by metabolic programs, epigenetic modifications, and transcriptional regulation.^107^ This stability is crucial for the ability of Tregs to perform their immunosuppressive functions effectively. Recent studies have highlighted the intricate regulation of Treg cell stability by metabolic programs.^107–109^ Metabolic pathways, including glycolytic flux, fatty acid β-oxidation (FAO), and amino acid catabolism, are essential for maintaining Treg cell stability.^106^ In addition to metabolic regulation, epigenetic mechanisms critically govern Treg cell stability.^110–112^ Epigenetic modifications, specifically DNA methylation and histone acetylation, play pivotal roles in stabilizing the Treg phenotype. These modifications act as molecular switches, turning genes on or off that are critical for Treg function. For instance, stable Treg cells tend to have demethylated *Foxp3* loci, whereas unstable Treg cells may exhibit methylation changes that compromise Foxp3 expression.^113^ Tregs maintain their identity and suppressive capabilities in a dynamic immune environment by regulating gene expression at the epigenetic level (Fig. 2a).^113^

Transcriptional regulation is another cornerstone of Treg stability.^114,115^ Key transcription factors, such as NFAT, STAT5, and Runx, orchestrate the expression of Foxp3 and other Treg-specific genes, such as *IL-2RA* and *IL-10*.^116,117^ These transcription factors synergistically assemble, binding to the promoters and enhancers of Treg-specific genes to ensure the robust expression of the Treg gene signature. For instance, NFAT is reactivated by calcium signaling to induce Foxp3 expression,^118^ thereby promoting Treg cell differentiation. In contrast, STAT5 is activated downstream of cytokine receptors, such as IL-2R, and is indispensable for Treg cell survival and proliferation.^119^ Additionally, these transcription factors regulate other genes crucial for Treg function, including those encoding negative immune cytokine superfamily members such as IL-10 and TGF-β (Fig. 2b).^120^ The elaborate transcriptional network governed by these factors mediates the distinctive transcriptional profile of Treg cells, which underpins their suppressive capacity and stability. Conserved noncoding sequences (CNS0–CNS3) within the *Foxp3* locus recruit transcription factors to regulate *Foxp3* expression.^58,121,122^ In Treg cells, CNS0 serves as a binding platform for the STAT5 transcription factor in the IL-2Rβ/JAK3–STAT5 signaling axis, thereby promoting the differentiation of thymic precursor cells into Tregs.^123^ The CNS1 element contains the TGF-β–NFAT response element,^58,124^ whereas the CNS2 element, also known as the TSDR, is enriched with CpG motifs, whose demethylation is critical for maintaining Foxp3 stability.^14,113,125–127^ Runx–CBFβ complexes are required for maintaining Treg stability by binding to the CNS2 locus of *Foxp3*.^128,129^ c-Rel binding to CNS3 enhances Treg production in the thymus and periphery. Additionally, CNS3 cooperates with CNS0 to initiate and stabilize Foxp3 expression, regulating Treg development, maintenance, and self-tolerance.^58,124^

### Plasticity of Tregs

**Fig. 3:**
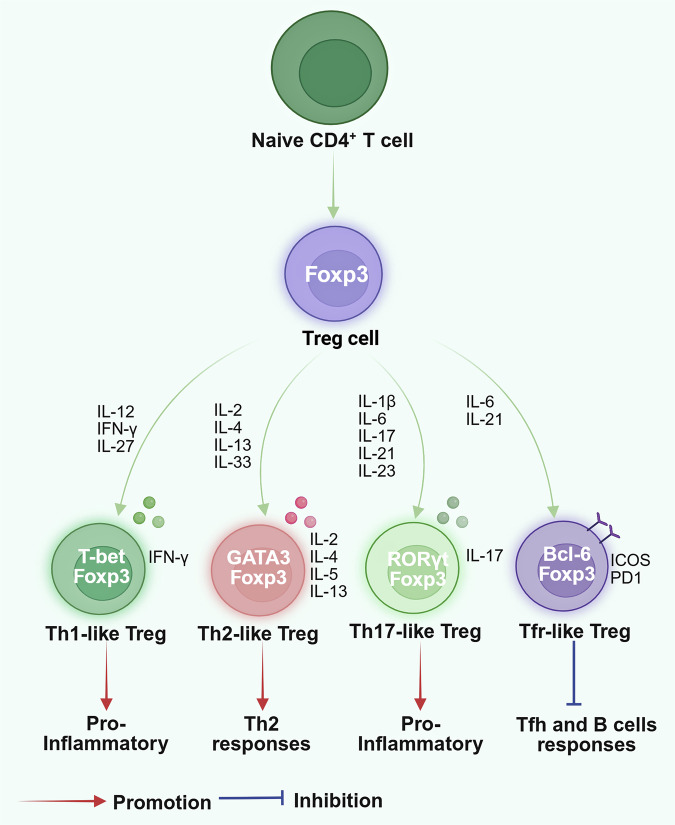
Treg cell plasticity. Tregs are reprogrammed into Th-like Tregs in response to various cytokines. Th1-like Tregs are induced by IL-12, IL-27, and IFN-γ; Th2-like Tregs are induced by IL-2, IL-4, IL-13, and IL-33; Th17-like Tregs are induced by IL-1β, IL-6, IL-17, IL-21, and IL-23; and Tfr-like Tregs are induced by IL-6 and IL-21.^544^ This figure was created with Biorender.com

The functional adaptability of regulatory T cells manifests as their capacity to undergo ‌transcriptional reprogramming‌, mimicking effector T lymphocyte differentiation pathways, thereby generating T helper lineage-mimicking Treg clusters‌ with ‌stable Foxp3 maintenance‌. This adaptation allows Tregs to exert targeted immunosuppressive functions in response to specific inflammatory signals and environmental conditions.^105,106^ Several Th-like Treg subsets have been identified, including type 1 Tregs (Tr1), which are defined by the co-expression of T-BET, CXCR3, and Foxp3; Th2-skewed Tregs (Gata3^+^IRF4^+^IL4^+^Foxp3^+^); RORγt+ Tregs, which are characterized by the dual expression of IL-17A and Foxp3; and follicular Tregs (Tfr-like Tregs) (BCL6^+^Foxp3^+^) (Fig. 3).^114,130–132^ Th1-like Tregs produce IFN-γ and upregulate Th1-associated markers, including T-BET and CXCR3. Their induction is driven by factors such as IL-12, IFN-γ, and IL-27.^133,134^ Th2-like Tregs with elevated IL-4, GATA3, and IRF4 expression appear under pathological conditions. They effectively suppress Th1 and Th17 responses,^135–137^ and exhibit greater migratory capacity, enabling rapid accumulation at tumor sites, where they contribute to a tumor-promoting environment.^137^ Th17-like Tregs express IL-17, IL-22, IL-23R, and RORγt,^138,139^ and are found primarily in the intestines of humans^140,141^ and mice.^142^ While their exact role remains unclear, these cells may be pathogenic, contributing to inflammatory bowel disease (IBD) and colon cancer.^42^ Tfr-like Tregs, characterized by BCL6 and Foxp3 co-expression, specialize in regulating follicular responses within germinal centers (GCs) and maintaining immune tolerance and B cell homeostasis.^143,144^

## New regulatory mechanisms of Tregs

### New cellular metabolism and signaling pathways

**Fig. 4:**
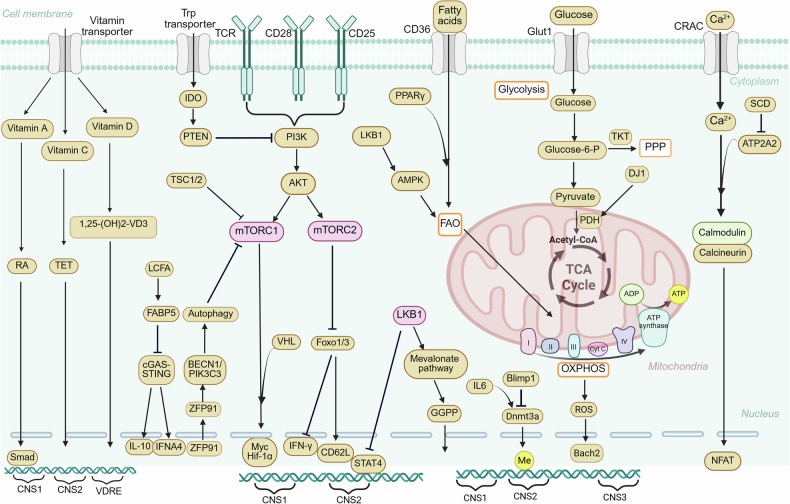
Overview of Treg metabolism. Foxp3 expression in Tregs is modulated by nutrient metabolites, intracellular metabolic intermediates, and signaling pathways. The vitamin A derivative RA promotes iTreg differentiation by enhancing TGF-β signaling via ERK1/2. Vitamin C stabilizes Foxp3 by demethylating CNS2. 1,25(OH)₂D₃ promotes Foxp3 expression by binding to the VDRE region. FABP5 inhibition enhances Treg suppressive function by activating the cGAS-STING signaling pathway. Upon TCR stimulation, ZFP91 translocates to the cytoplasm, facilitating BECN1-PIK3C3 complex formation and enhancing TCR-induced autophagy, supporting Treg metabolism and function. LKB1 maintains Foxp3 stability by inhibiting STAT4-driven CNS2 methylation and activating the mevalonate pathway, which results in the production of metabolites such as cholesterol and GGPP. Blimp1 inhibits Dnmt3a upregulation and prevents CNS2 methylation at the *Foxp3* locus, thereby maintaining Treg stability and function. SCD1 deficiency upregulates ATP2A2, which, upon TCR activation, enhances the calcium–NFAT1–Foxp3 axis, promoting Treg differentiation. This figure was created with Biorender.com

Cellular metabolism is essential for regulating various aspects of Treg cells, including their stability, function and differentiation. The key metabolic signaling pathways involved include nutrient uptake, glycolysis, mitochondrial metabolism, and lipid and fatty acid metabolism (Fig. 4). Notably, immune receptors and metabolism-related environmental signals, such as nutrients, interact extensively to mediate immune responses in vivo.^145,146^ Among these metabolic regulators, all-trans retinoic acid (RA), a vitamin A derivative, promotes iTreg differentiation by enhancing TGF-β signaling through the ERK1/2 pathway.^147^ Similarly, vitamin C facilitates the demethylation of the CpG motif in *Foxp3* CNS2 through ten-eleven translocation (TET2), promoting stable Foxp3 expression.^148,149^ Additionally, 1,25-dihydroxyvitamin D₃ (1,25(OH)₂D₃) enhances Foxp3 expression by binding to the vitamin D response element (VDRE) within the CNS region of the human *Foxp3* gene.^150–152^ Tregs depend primarily on lipid oxidative phosphorylation (OXPHOS), similar to memory T cells, in contrast to the glycolytic preference observed in effector T cells.^106,153–156^ Fatty acid binding protein 5 (FABP5) is a member of the lipid chaperone family. Inhibition of FABP5 in Tregs induces mitochondrial DNA (mtDNA) release, activating the cGAS-STING signaling pathway. This pathway boosts IL-10 and IFNA4 production and reinforces the suppressive function of Tregs.^157^ Stearoyl-CoA desaturase 1 (SCD1) is the rate-limiting enzyme in oleic acid synthesis during fatty acid metabolism. SCD1 or oleic acid deficiency elevates H3K79me2 at the ATP2A2 locus, increasing ATP2A2 expression. This activation enhances the calcium–calcineurin–NFAT axis, driving Tconvs differentiation into Tregs in the mouse thymus.^158^ CD36, a scavenger receptor, plays a key role in long-chain fatty acid absorption and lipid metabolism. The activation of peroxisome proliferator-activated receptor gamma (PPARγ) increases the frequency of Treg cells by increasing CD36/carnitine palmitoyltransferase-1 (CPT-1)-driven FAO and the subsequent N-glycosylation of TβRII/IL-2Rα, thereby promoting Treg function.^159^ In tumors, CD36 deletion in Tregs alters fatty acid metabolism, increasing IFN-γ and TNF-α production while reducing tumor infiltration.^160^ Furthermore, CD36-PPAR-β signaling regulates mitochondrial fitness and lactate metabolism, which are crucial for Treg survival. CD36 blockade via monoclonal antibodies enhances antitumor immunity, offering a strategy to reshape the TME while preserving systemic immune balance.^161^ Vacuolar protein sorting 33B (Vps33B) maintains the homeostasis of the endosome/lysosome system and regulates the activation of the amino acid signal-dependent mTORC1 pathway and cellular metabolism, thereby maintaining the suppressive function of Treg cells.^50^ Myc regulates Treg accumulation and functional activation by modulating mitochondrial oxidation, independent of FAO. Myc-deficient Tregs exhibit defective accumulation and activation, leading to autoimmune diseases accompanied by effector T cell responses.^162^ The enzyme pyruvate dehydrogenase (PDH) serves as a regulator of the tricarboxylic acid (TCA) cycle. Park7 (DJ-1) binds to PDHE1-β (PDHB) to promote PDH activity and OXPHOS. Treg-specific DJ-1 deletion reduces Treg survival in mice and reduces Treg stability and number in aged mice.^163^ Methylenetetrahydrofolate dehydrogenase-2 (MTHFD2) is a mitochondrial enzyme involved in folate metabolism. MTHFD2 deficiency enhances Treg differentiation by depleting purine pools, increasing the accumulation of purine biosynthetic intermediates, and reducing mTORC1 signaling.^164^ Moreover, the metabolism of nicotinamide adenine dinucleotide (NAD^+^) is linked to the regulation of Treg cell function. NAD^+^ levels influence the activation and function of Tregs, indicating that the modulation of NAD^+^ metabolism may affect Treg cell-mediated immune tolerance.^165^ Treg cells in the TME highly express the immune checkpoint receptor PD-1. Deleting or inhibiting PD-1 in Treg cells can delay tumor progression by weakening lipid metabolism and the proliferation of TI-Tregs.^166,167^ Additionally, in mice lacking PD-L2, pTreg-specific knockout of PD-L2 reduced the number of pTregs at steady state and their suppressive activity. Transketolase (TKT) is an essential enzyme in the pentose phosphate pathway (PPP). TKT deficiency disrupts Treg suppressive activity by causing uncontrolled OXPHOS while maintaining normal Treg cell numbers.^168^ Mitochondrial respiratory chain complex III knockout in Treg cells impairs their suppressive function without altering their cell number in mice.^169^ Mitochondria-derived reactive oxygen species (ROS) reportedly increase the stability of SUMO-specific protease 3 (SENP3) by inhibiting its ubiquitin-mediated degradation,^170,171^ and triggering the deSUMOylation of the transcription factor BACH2.^172^ SENP3-mediated BACH2 deSUMOylation prevents its nuclear export, thus maintaining Treg stability and function.^172^ Glycolysis is key for Treg cell migration to inflamed tissues and is mediated by glucokinase induced by the PI3K‒mTORC2 pathway.^173^ Additionally, the PI3K–AKT–mTORC2 pathway promotes Treg migration to peripheral nonlymphoid organs while limiting lymphoid recirculation by suppressing forkhead box O 1/3 (Foxo1/3).^174^ Furthermore, hypoxia-inducible factor (HIF)-1α, a transcription factor, has been reported to increase glycolysis, induce Foxp3 expression,^175,176^ and enhance the migration of Treg cells.^177^ Consistent with these reports, VHL, an E3 ubiquitin ligase, is essential for Treg stability and suppressive function through the regulation of the HIF-1α pathway.^178^ Treg cells in human tumors increase glucose consumption, promote cell senescence and suppress effector T cells, which hinder tumor immunotherapy. The activation of Toll-like receptor 8 (TLR8) impairs glucose uptake and glycolysis in Tregs, disrupting their function and enhancing antitumor immunity.^179^

Liver kinase B1 (LKB1) is a kinase that phosphorylates and activates AMP-activated protein kinase (AMPK), triggering metabolic adaptations to restore energy balance.^180–182^ Emerging evidence suggests that LKB1 is essential for regulating Treg cell metabolism and stability. Interestingly, these events seem to occur independently of AMPK,^183–186^ indicating the involvement of other downstream targets in Treg metabolic regulation. Recent studies on Tregs lacking LKB1 revealed that LKB1 maintains Foxp3 stability by inhibiting STAT4-driven methylation of CNS2 at the *Foxp3* locus,^183^ and by activating the mevalonate pathway, which produces key metabolites such as cholesterol and the isoprenoid geranylgeranyl pyrophosphate (GGPP). Moreover, treatment with mevalonate or GGPP rescues the function and stability of Tregs lacking LKB1.^184^ In addition, LKB1 function in Treg cells also contributes to the activation of β-catenin signaling, regulating immune regulatory molecules such as PD-1, GITR, and OX40 to effectively suppress Th2 responses.^185^ The function of LKB1 in Tregs is driven by MAP/microtubule affinity-regulating kinases (MARKs) and salt-inducible kinases (SIKs).^186^ However, the precise mechanisms regulating LKB1 activation in Treg cells require further investigation. Amino acids support Treg cell function through TCR-induced mTORC1 activation. The RagA and RheB GTPases play key roles in amino acid-driven mTORC1 activation in Tregs. Treg-specific RagA-RagB- or Rheb1-Rheb2-knockout mice exhibit impaired amino acid metabolism, reduced Treg cell proliferation, and fatal autoimmune diseases.^187^ Notably, Tregs in peripheral tissues, especially tumors, show increased sensitivity to nutrient sensing via Rag GTPases. Selective deletion of RagA reduces Treg infiltration in tumors, thereby enhancing antitumor immune responses.^188^ Recent research has shown that the IL-33/ST2 signaling pathway plays a very important role in ST^+^ Treg cells. IL-33 facilitates the accumulation and maintenance of ST2^+^ Tregs in the intestine, thereby enhancing their protective role in intestinal inflammation.^189^ Lymphoid-derived visceral adipose tissue (VAT) Treg precursors (PPARγ^low^), guided by tissue-specific TCR signals via C-C motif chemokine receptor 2 (CCL2), migrate to the VAT, where IL-33 drives their local expansion and differentiation into ST2^+^ VAT Tregs (PPARγ^hi^).^190–193^ In individuals with inflammatory bowel disease, RORγt^+^ Treg cells expand and express pro-inflammatory cytokines via Wnt–β-catenin signaling, which disrupts transcription factor T cell factor 1 (TCF-1) and Foxp3 regulation of pro-inflammatory genes, promoting a disease-associated phenotype.^194^ The release of extracellular adenosine triphosphate (ATP) by dying and inflammatory cells impairs the suppressive function and stability of Tregs by activating purinergic P2X receptors. Inhibition of the extracellular ATP-P2X signaling pathway enhances Treg function in the muscle inflammatory response and slows the progression of the muscular dystrophy phenotype.^195–197^

### New molecular mechanisms

The molecular mechanisms regulating Foxp3 expression and function have been extensively studied. Recent studies have highlighted the critical role of Blimp1 in preserving Treg identity, particularly under inflammatory conditions. IL-6 signaling activates Dnmt3a, a DNA methylase that, in the absence of Blimp1, targets distinct DNA sites, leading to CNS2 methylation and Foxp3 downregulation. Conversely, Blimp1 suppresses Dnmt3a upregulation, preventing CNS2 methylation at the *Foxp3* locus and thereby maintaining Treg stability and function. Consequently, Blimp1 deficiency in Tregs under inflammatory conditions triggers CNS2 methylation, leads to *Foxp3* downregulation, and drives a shift toward a pro-inflammatory T cell phenotype.^198^ SET domain-containing 2 (SETD2) is a type of histone methyltransferase that can catalyze lysine 36 trimethylation on histone 3 (H3K36me3)^199^ and has been recently reported to play an important role in the generation and function of different types of cells.^200–203^ An animal study demonstrated that SETD2 sustains GATA3⁺ST2⁺ intestinal thymic-derived Treg (tTreg) cells by promoting GATA3 and ST2 expression and maintaining their reciprocal interaction. The SETD2-mediated regulation of GATA3 expression in Tregs is evolutionarily conserved between mice and humans and is crucial for the selective suppression of Th2 responses. Additionally, Setd2 modulates Treg cell promoters and intragenic enhancers, epigenetically regulating target gene transcription via H3K36me3-marked enhancers. However, the precise molecular mechanisms underlying this regulation remain elusive.^204^ Autoimmune diseases are prone to occur during Treg cell depletion therapy for tumors. Therefore, targeted identification of Treg cells in tumors is particularly important. Recently, a report reported that JMJD1C is a histone demethylase in the tumor microenvironment that regulates the adaptability of Treg cells in tumors but does not affect systemic immune homeostasis. JMJD1C deficiency enhances AKT and STAT3 signaling, leading to massive production of interferon-γ and a reduction in the number of tumor Treg cells.^50^ In addition to epigenetic regulation, other factors, including costimulatory signals, splicing factors, and ubiquitination modifications, also influence Foxp3 expression and function. Steroid nuclear receptor coactivator 2 (SRC2), a transcription coactivator, is crucial for Treg differentiation. SRC2 promotes Treg induction by upregulating Nr4a2, thereby facilitating immune tolerance.^156,205^ Recurrent somatic mutations in spliceosome factor 3b subunit 1 (SF3B1), particularly SF3B1-K700E, are frequently found in hematopoietic malignancies. Treg-specific expression of SF3B1-K700E leads to spontaneous autoimmune phenotypes. Mechanistically, SF3B1-K700E disrupts splicing, leading to reduced expression of Anapc13, a key regulator of cell proliferation. Restoring Anapc13 expression rescues Treg differentiation and restores their ability to prevent adoptive transfer-induced colitis.^206^ The E3 ubiquitin ligase ZFP91 promotes autophagy activation in Treg cells, supporting their metabolic programming and function. Mechanistically, upon TCR stimulation, ZFP91 quickly moves from the nucleus to the cytoplasm, where it mediates BECN1 ubiquitination, facilitating BECN1-PIK3C3 complex formation and enhancing TCR-induced autophagy activation and related downstream pathways.^207^ Moreover, Treg-specific loss of Rbx1, an E3 ligase with dual roles in neddylation and ubiquitylation through Cullin-RING ligases, induces severe inflammation by impairing key effector Treg functions and regulatory pathways. Similarly, Ube2m deletion in Treg cells, another neddylation-related enzyme, causes milder inflammation. In contrast, the loss of Rbx2/Sag or Ube2f, key components of the neddylation-CRL complex, has no significant impact. This finding underscores the distinct function of the Ube2m-Rbx1 axis in Treg regulation and suggests additional functions of Rbx1 beyond its interaction with Ube2m.^208^ RNF213, a RING finger protein, facilitates Treg differentiation and attenuates autoimmune disease via Foxo1 signaling. Mechanistically, it interacts with Foxo1, facilitating its nuclear translocation through K63-linked ubiquitination.^209^ Tumor microenvironment stressors induce the deubiquitinases Usp22 and Usp21, which are crucial for Treg cell fitness. Their deletion impairs Treg function and enhances antitumor immunity, highlighting Usp22 as a potential target for cancer therapy.^210^ The transcription factor BACH2 is expressed at elevated levels in resting Tregs, whereas its expression is diminished in activated Treg cells and during inflammatory responses. Within resting Tregs, BACH2 binds to the gene enhancer region that regulates activated Treg cell differentiation and inhibits TCR-driven activation of these genes by competing with AP-1 transcription factors for DNA-binding sites. This action of BACH2 helps maintain the resting state of resting Tregs and their long-term survival, which is necessary to maintain immune balance and achieve durable immunosuppression in cancer.^211^

## Role of Tregs in homeostasis and diseases

**Fig. 5:**
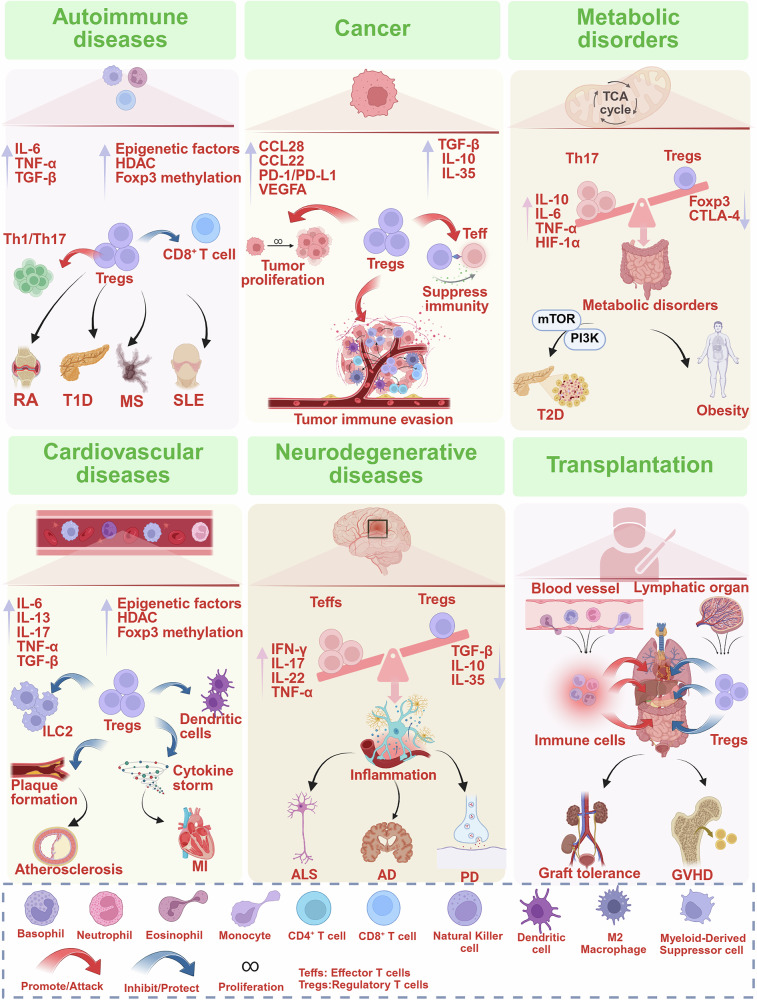
Role of Tregs in homeostasis and diseases. Tregs are dysfunctional or reduced in number, leading to decreased immune tolerance and the onset of autoimmune diseases. Tregs inhibit antitumor immunity, promote tumor progression, and facilitate immune evasion in cancer immunotherapy. Treg dysfunction drives chronic inflammation and insulin resistance, accelerating the development of T2D and obesity. Tregs secrete anti-inflammatory factors and suppress pro-inflammatory cells, oxidative stress, and plaque progression, thereby limiting immune activation in atherosclerosis and myocardial infarction. Tregs dysfunction or depletion in neurological diseases disrupts the Th17/Treg balance, driving neuroinflammation and accelerating disease progression. Tregs mitigate immune rejection during transplantation by suppressing rejection responses and minimizing adverse effects. This figure was created with Biorender.com

Tregs are essential for maintaining immune balance, and their dysfunction or dysregulation can lead to a range of immune-related diseases. Understanding the role of Tregs in homeostasis and human diseases is critical for developing Treg-centered therapeutic strategies (Fig. 5).

### Treg cells in autoimmune diseases

Within the spectrum of prevalent autoimmune conditions, rheumatoid arthritis (RA), type 1 diabetes (T1D), multiple sclerosis (MS), and systemic lupus erythematosus (SLE), Tregs are often dysfunctional or reduced in number, contributing to the breakdown of immune tolerance and the onset of disease. Understanding the complex interplay between Tregs and autoimmune diseases could elucidate ‌druggable targets‌ for novel therapeutic interventions for these immune dysregulation syndromes.

#### Rheumatoid arthritis

In the context of RA, Tregs function as guardians of immune tolerance, suppressing excessive immune activation and inflammation.^212^ The imbalance between effector T cells and Tregs is considered crucial in RA pathogenesis.^213–215^ Recent studies have demonstrated that CD8^+^ Tregs expressing Foxp3 can be stably generated in vitro via the use of TGF-β1 and rapamycin (RAPA). These TGF-β1/RAPA-induced human CD8⁺ Tregs exhibit robust suppressive function, characterized by high expression of Foxp3 and CD103. Notably, they significantly ameliorated collagen-induced arthritis (CIA) in mice, as evidenced by reduced clinical scores, decreased anti-collagen IgG levels, and diminished cartilage destruction.^216^ Additionally, Foxp3⁻CD8⁺ Tregs play non-redundant roles in autoimmune and infectious diseases by suppressing autoreactive CD4^+^ T cells, limiting tissue damage, and promoting the resolution of inflammation.^217,218^ Emerging evidence suggests that the function and number of Tregs are compromised in patients with RA.^212,219^ Studies have shown that RA patients exhibit reduced Treg frequencies, impaired suppressive function, and decreased CTLA-4 expression in peripheral blood and synovial fluid.^220,221^ Epigenetic modifications, including DNA methylation and histone deacetylation, further influence Treg differentiation and function in RA.^112,115,213,222^ Clinical trials are assessing the effectiveness of adoptive transfer of ex vivo-expanded functional Tregs and Treg-stimulating agents such as IL-2 analogs and novel small molecules.^223^ A recent study explored the impact of inhibiting IL-6 signaling on various subpopulations of Tregs in RA, potentially improving patient prognosis.^224^ These innovative approaches seek to leverage the inherent regulatory capabilities of Tregs to restore immune homeostasis, alleviate RA symptoms, and potentially arrest the progression of the disease in human patients.

#### Type 1 diabetes

In individuals with T1D, Tregs are often impaired or reduced in number, allowing autoreactive immune cells to attack and destroy β-cells.^225^ Recent studies highlight the crucial role of Tregs in T1D pathogenesis, with genome-wide association studies linking genetic susceptibility to immune cell function, particularly genes involved in Treg development and function.^226,227^ Functional studies in mouse models and peripheral blood analyses of T1D patients provide strong evidence of Treg dysfunction.^228^ Moreover, enhancing Treg function or increasing their population has been revealed to suppress or delay T1D onset, preserve β-cell mass, and maintain normoglycemia.^229^ These findings support the ongoing exploration of Treg-based therapies for T1D.

#### Multiple sclerosis

The pathological hallmarks of MS include inflammation, demyelination, axonal damage, and gliosis. Self-reactive CD4⁺ T cells, especially Th1 and Th17 subsets, play crucial roles in triggering and maintaining the inflammatory cascade in MS.^230^ These T cells infiltrate the CNS and interact with resident immune cells, such as microglia and astrocytes, to promote demyelination and neurodegeneration.^231^ Additionally, the E3 ubiquitin ligase RNF213 is known to facilitate K63-type ubiquitination and nuclear import of Foxo1, thereby enhancing Treg cell development and mitigating autoimmune diseases such as MS.^209^ Current therapeutic strategies for MS aim to modify the disease course and reduce relapses. The most widely used drugs include interferon-β, glatiramer acetate, natalizumab, fingolimod, dimethyl fumarate, and teriflunomide. Emerging therapies targeting the immune system, such as B-cell depletion (ocrelizumab) and IL-2 receptor blockade (daclizumab), have shown promise in reducing relapses and slowing disease progression in some patients.^232–234^ Ongoing research into the molecular mechanisms underlying MS pathogenesis, combined with advances in immunotherapies, shows potential for the development of innovative therapeutic approaches.

#### Systemic lupus erythematosus

Tregs play a pivotal role in maintaining immune tolerance and preventing autoreactive responses in SLE.^235^ Recent research on SLE has focused on understanding the underlying immunopathogenesis and developing novel therapies. For instance, CAR-T cell therapies targeting B cells, particularly anti-CD19 CAR-T cells, have shown promise in refractory SLE patients, as they result in B cell depletion and disease improvement.^236,237^ Moreover, the ability of IL-2 immunotherapy to selectively expand Tregs is being explored, and low-dose IL-2 can improve disease activity in SLE patients by expanding functional Treg subsets, which play crucial roles in maintaining immune tolerance.^238^ These therapies aim to restore immune homeostasis, reduce systemic inflammation, and improve the clinical outcomes of patients with SLE. Future research in SLE will likely focus on refining and optimizing CAR-Treg therapies.

### Treg cells in cancer

#### Solid tumors

Compared with those found in peripheral blood and other tissues, the characteristics of Tregs in the TME are distinct. In blood and lymphoid tissues, Tregs typically maintain a relatively stable frequency, ranging from 5% to 10%. However, within tumors, they tend to accumulate and can account for more than 50% of all T cells.^239,240^ Many studies have shown that tumor-infiltrating Treg cells usually indicate a poor prognosis in most solid tumors, whereas in colorectal and endometrial cancers, the presence of Treg cells is linked to a better prognosis.^241^ However, several reports have suggested that increased Foxp3^+^ expression in colorectal cancer is associated with poor prognosis.^242,243^ These conflicting findings suggest that the role of Treg cells in colorectal cancer is context dependent and may vary on the basis of factors such as tumor stage, localization within the tumor microenvironment, or the presence of specific microbial or inflammatory signals.

Tregs influence tumor growth, invasion, and metastasis in the TME. They express chemokine receptors (e.g., CCR4, CCR8, and CCR10), facilitating their recruitment to tumors.^244,245^ First, Tregs attenuate the antitumor activity of CD8^+^ T cells by competitively inhibiting CD28 co-stimulatory signaling through CTLA-4, depleting IL-2, and releasing adenosine.^70,240^ Second, they secrete immunosuppressive cytokines (IL-10 and TGF-β) and modulate the local immune state via metabolic pathways such as IDO, thereby creating an immunosuppressive environment.^246,247^ Third, Tregs also regulate the tumor immune microenvironment by suppressing various immune cells (conventional T cells, Teffs, B cells, NK cells, NKT cells, and monocytes)^248,249^ and promoting the accumulation of immunosuppressive cells such as M2 macrophages and myeloid-derived suppressor cells.^250,251^ The stability of Tregs is also influenced by the local environment. In the TME, factors such as hypoxia, metabolic changes (e.g., lactate accumulation), and pro-inflammatory or immunosuppressive cytokines (e.g., TGF-β and IL-10) enhance the immunosuppressive function of Tregs, promote their expansion, and maintain their stability. Additionally, tumor-associated macrophages (TAMs) in the TME facilitate Treg enrichment and the conversion of CD4⁺ T cells into Tregs by expressing molecules such as PD-L1 and IDO1. However, the plasticity of Tregs can also change under certain conditions. For example, under specific inflammatory conditions, some Tregs may lose their suppressive function and convert into effector T cells.^252^ Furthermore, VEGF and vascular regulatory mechanisms may affect the distribution and function of Tregs in the TME; therefore, TME-targeted strategies (such as IL-2 variants) are being explored to selectively enhance T effector cell function without activating Tregs.

#### Hematologic tumors

Tregs in hematological malignancies exhibit distinct characteristics and prognostic roles. Higher Treg levels indicate better outcomes in follicular lymphoma (FL) and diffuse large B-cell lymphoma (DLBCL) but worse prognoses in chronic lymphocytic leukemia (CLL), “activated B cell” (ABC) DLBCL, and “non-specific” (NOS) DLBCL.^253^ In CLL, increased Tregs correlate with advanced disease stage, high CD38 expression, and a shorter time to first treatment.^254^ Multiplex sequencing identified three Treg clusters, including an unconventional Foxp3-negative subset (high LAG3, CTLA4, and KLRB1). Activated Tregs have distinct gene expression patterns, including those of the TNFRSF4, TNFRSF18, and NF-κB pathway genes.^255^ Tregs are more abundant in the newly diagnosed and blast crisis phases of CML, potentially driving progression through immunomodulation.^147^ In the blood tumor microenvironment, Treg-derived IL-21 suppresses effector T cells and NK cells^256^ and regulates B cells,^257^ promoting immunosuppression. In AML, IL-21 reduces leukemia stem cell (LSC) self-renewal, promotes asymmetric division via p38-MAPK signaling, decreases LSC numbers, and improves survival in mice. It also serves as a prognostic biomarker and enhances treatment efficacy in vitro, while low-dose IL-21 prolongs AML survival.^258^ In mantle cell lymphoma (MCL), SOX11 expression is correlated with Treg infiltration and an immunosuppressive environment, potentially driving tumor progression.^259^ Tregs also express CD39/CD73, depleting ATP and generating adenosine, which exhausts effector T cells.^260,261^

In summary, Tregs exhibit functional differences between solid and hematological tumors. In solid tumors, Tregs are primarily recruited through chemokine axes such as CCL28‒CCR10 and CCL22‒CCR4, whereas in hematologic malignancies, the CXCR4‒SDF1 axis facilitates Treg accumulation in the bone marrow or lymphoid tissues.^262^ Phenotypically, Tregs in solid tumors exhibit a tissue-resident profile characterized by high expression of Foxp3, CTLA-4, and CD39, whereas hematologic malignancies predominantly harbor circulating Tregs (Foxp3^+^CD25^+^), with some subsets exerting functions within the bone marrow microenvironment. The immunosuppressive strategies also differ, with solid tumor Tregs relying on cytokine-mediated suppression via TGF-β, IL-10, and IDO, whereas in hematologic malignancies, Tregs can directly inhibit B cells and NK cells or modulate myeloid cell differentiation. Functionally, solid tumor Tregs primarily contribute to immune evasion and the suppression of antitumor immunity, whereas in hematologic malignancies, they may influence the bone marrow microenvironment and promote leukemia stem cell survival.

### Treg cells in metabolic disorders

#### Type 2 diabetes

Newly discovered evidence indicates that Tregs are intricately involved in the development of T2D.^263^ Yuan et al. reported that the number of circulating Tregs was negatively correlated with fasting blood glucose concentrations in individuals with impaired glucose tolerance.^264^ The pro-inflammatory milieu in T2D is hypothesized to disrupt the Th17/Treg balance, favoring Th17 expansion and promoting chronic inflammation and metabolic dysfunction.^265^ Tregs are crucial regulators of glucose metabolism and insulin sensitivity.^266,267^ Notably, T2D patients exhibit reduced CD3⁺ T cells, suggesting a weakened immune response.^268^ Given their important role in immune regulation and metabolism, Tregs have emerged as promising treatment options for T2D. Research conducted by Liu et al. revealed that the infusion of Tregs enhanced insulin responsiveness and glucose tolerance in ob/ob mice, a commonly used model of T2D.^269^ Similarly, other studies have reported that treatment with the IL-2/anti-IL-2 complex, which expands Tregs in vivo, can ameliorate insulin resistance and glucose intolerance in animal models of T2D.^270^ These findings suggest that enhancing Treg function may represent a novel therapeutic approach for T2D.

#### Obesity and other metabolic syndromes

Chronic low-grade inflammation in adipose tissue, driven by immune cell infiltration, is a key driver of obesity-related metabolic disorders.^271^ The intricate interplay between the immune system and metabolic pathways under these conditions has garnered significant attention, particularly with respect to the role of Tregs.^266^ VAT-resident Treg cells have been shown to have a unique transcriptional profile, which is driven primarily by the nuclear receptor family member PPARγ.^272^ The accumulation of plasmacytoid dendritic cells (pDCs) and the subsequent production of IFN-α during obesity lead to the depletion of PPARγ^+^ VAT-Treg cells. Blocking the IFN-α pathway in obese mice was found to restore VAT-Treg cell levels and enhance insulin sensitivity, indicating a potential therapeutic target.^273^ In addition to their role in obesity, Treg cells also play a vital role in other metabolic tissues, including the liver and skeletal muscle. In the liver, Treg-exclusive long non-coding RNA (lncRNA) Altre preserves the immune–metabolic balance by regulating mitochondrial function and Treg survival.^274^ In skeletal muscle, exercise has been shown to activate Treg cells, contributing to muscle regeneration and repair. Muscle-resident Treg cells exhibit unique transcriptional profiles and express factors such as Foxp3 and amphiregulin, which promote tissue growth.^275^

### Treg cells in cardiovascular diseases

Cardiovascular diseases (CVDs), encompassing conditions such as atherosclerosis and myocardial infarction, constitute the primary cause of death globally.^276^ The intricate interplay between immune system components and the cardiovascular system is crucial in the development of these conditions.^277^ Among immune cells, Tregs have emerged as critical modulators of immune homeostasis and inflammation, thereby influencing the progression and outcomes of CVDs.^278^

#### Atherosclerosis

The role of the immune system, particularly Tregs, in the development and progression of atherosclerosis has been the subject of intensive research.^279^ Recent studies have shown that the number and function of Tregs are reduced in patients with atherosclerosis, suggesting their involvement in disease pathology.^280^ Reports indicate that the production of IL-17 by CD4^+^CD45RO^+^Foxp3^+^ T cells in the bloodstream is linked to atherosclerosis,^281^ suggesting that certain Treg subsets might exhibit pro-inflammatory properties under specific conditions, contributing to the complexity of Treg function in this disease. Tregs are emerging as promising therapeutic targets in atherosclerosis because of their ability to modulate immune-inflammatory pathways and stabilize plaques. Key molecules such as IL-10, TGF-β, and CTLA-4 mediate Treg-mediated suppression of pro-inflammatory cells, oxidative stress, and plaque progression. IL-35-producing Tregs further inhibit proatherogenic immune activation. By enhancing Treg function or numbers, novel therapies could attenuate vascular inflammation, promote tissue repair, and reduce atherosclerosis severity, offering a strategic shift toward immune regulation in cardiovascular disease management.^282,283^

#### Myocardial infarction

Myocardial infarction (MI), the most common form of acute heart injury, is a leading cause of mortality worldwide.^284^ Tregs migrate to infarcted hearts post-MI, suppressing inflammation and fibrosis by promoting macrophage repair phenotypes via anti-inflammatory factors (e.g., IL-10 and nidogen-1) and inhibiting damage to CD8^+^ T cells.^285–287^ Preclinical studies have shown that exogenous Treg administration reduces cardiomyocyte death and fibrosis, improving cardiac function. Gene-modified Tregs may increase therapeutic stability, addressing challenges in post-MI repair.^288,289^ In addition, local treatment with Tregs significantly enhances tissue regeneration and wound healing in mouse models of skeletal, muscle, and skin injury. Tregs promote the switch of monocytes and macrophages to an anti-inflammatory state, particularly through the secretion of IL-10.^290^ Moreover, genetically modified Tregs are promising for addressing the challenges faced by conventional Treg cell therapies, such as maintaining long-term stability and efficacy, which are crucial for their application in post-MI heart repair.^291^ These results emphasize the importance of Tregs in facilitating heart regeneration and suggest their possible application as an innovative immunotherapy approach for MI.

### Treg cells in neurodegenerative diseases

#### Amyotrophic lateral sclerosis

Amyotrophic lateral sclerosis (ALS) is a progressive neurodegenerative disease characterized by motor neuron degeneration and inflammation, leading to respiratory paralysis and death.^292,293^ Treg cells are crucial in the pathogenesis of ALS.^294^ Tregs can suppress the pro-inflammatory responses of Teff cells and myeloid-derived cells, thereby providing protective effects in ALS patients.^295,296^ Consequently, numerous reports have proposed the use of Treg cells as a potential therapeutic strategy for ALS, and their neuroprotective effects have been further investigated in clinical studies. Solmaz et al. reported that increased effector T cells in the blood and cerebrospinal fluid of ALS patients correlate with poorer survival, whereas higher activated Treg levels and a greater activated-to-resting Treg ratio are linked to improved survival.^297^ Beers et al. demonstrated that the adoptive transfer of endogenous Tregs from early-stage mSOD1 mice into late-stage ALS disease model mice helped sustain the M2 microglial phenotype, thereby extending disease duration and improving survival outcomes in the recipients.^298^ Sheean et al. provided compelling evidence that the immune system regulates disease progression in ALS patients and highlighted the crucial role of Tregs in disease pathobiology.^299^ This study suggested that expanding peripheral Treg cells may have a beneficial effect on suppressing toxic neuroinflammation within the brains of ALS patients. IL-2 is essential for the survival and function of Tregs,^300–302^ and Tregs are highly sensitive to low environmental levels of IL-2.^303–306^ IL-2 therapy has been shown to restore the immunosuppressive function of Tregs in vitro.^300,307^ A clinical study (NCT04055623) reported the safety profile and protective role of combined Treg/IL2 treatments in ALS patients,^308^ and IL-2 administration has been proposed as a novel therapeutic strategy. The IMODALS study (NCT02059759) reported the pharmacodynamics and safety of low-dose (ld) IL-2, which demonstrated a significant increase in Treg cell numbers without any serious adverse events. However, researchers suspect that the molecular benefits linked to changes in Treg numbers merit further investigation.^309^ Notably, Giovannelli et al. conducted a subgroup analysis within the same IMODALS cohort via microarray gene expression profiling, identifying transcripts related to lipid metabolism that were associated with drug response.^310^ Overall, these data provide new evidence for the role of Treg cells in ALS disease progression and may offer a promising treatment option for patients with this disease.

#### Alzheimer’s disease

Alzheimer’s disease (AD) is a progressive neurodegenerative disorder and the leading cause of dementia.^311,312^ Effector T cells (Th1 and Th17) and Treg cells have been associated with AD.^313–315^ CD4^+^ effector T cells expedite AD progression in APP/PS1 mice.^316^ Furthermore, early depletion of Tregs is linked to accelerated cognitive impairment, while restoring Tregs has been shown to improve cognitive function.^317^ Treg-mediated neuroprotection is widely recognized, but its therapeutic potential is limited by a lack of specific antigen targeting. Recently, researchers have engineered Tregs with Aβ-specific T cell receptors (TCR Aβ). These TCR Aβ-Tregs help clear Aβ deposits, shift effector cells toward a regulatory phenotype, and reduce neurotoxicity, showing promise in AD models.^43,44^ Scientists have created a lipid nanoparticle nanovaccine with Aβ peptides and rapamycin, effectively targeting dendritic cells to produce anti-Aβ antibodies and Aβ-specific Tregs, clear Aβ plaques in the brain, reduce neuroinflammation, and prevent cognitive decline in mice.^318^ These observations suggest that targeting Tregs is a promising therapeutic strategy for AD.

#### Parkinson’s disease

Parkinson’s disease (PD) is the second most common neurodegenerative disease after AD and is expected to affect more than 1.2 million people by 2030.^319–321^ Treg cells are crucial for limiting neurodegeneration in PD, as shown in animal models. The depletion of Tregs has been shown to exacerbate neuroinflammation in a 1-methyl-4-phenyl-1,2,3,6-tetrahydropyridine (MPTP) mouse model of PD.^322,323^ Furthermore, Treg enhancement, induction, or transfer has been demonstrated to promote neuronal survival and reduce neuroinflammation in a PD mouse model,^324,325^ highlighting their direct role in neuroprotection. Moreover, expanding dysfunctional Tregs isolated from PD patients, both in vivo and in vitro, successfully restored their suppressive function.^326–328^ Tregs from patients with PD exhibit impaired abilities to suppress effector T cell functions.^329^ Moreover, adoptive transfer of Tregs has been demonstrated to confer greater than 90% protection to the nigrostriatal system in patients with PD.^330^ Tregs mitigate effector immune responses, uphold central and peripheral tolerance, and reduce neuroinflammation.^331^ In addition to being suppressed, they also redirect neurodestructive Th1 and Th17 responses toward neuroprotection.^330^ The loss of midbrain dopamine neurons (mDANs) leads to motor dysfunction in PD, making cell replacement a promising therapy.^332–334^ However, the poor graft survival of mDANs hinders clinical success.^335,336^ Recent studies suggest that co-transplantation of mDAPs and Tregs modulates the host immune response triggered by surgery and significantly protects mDANs while inhibiting the proliferation of TH cells. This approach may enhance the effectiveness and safety of cell replacement therapy for PD and other neurodegenerative diseases.^337^ Taken together, these findings suggest that Treg cells may constitute a novel therapeutic option for patients with PD.

### Treg cells in transplantation

#### Organ transplant

Solid organ transplantation is crucial for end-stage organ failure, but immune rejection hinders graft success and long-term survival. Tregs can improve outcomes by reducing rejection and adverse effects.^338^ In kidney,^339^ liver,^340^ and heart^341^ transplants, increasing Treg quantity and function post-surgery is linked to lower rejection rates and better graft survival, highlighting their role in maintaining graft stability. Tregs reduce graft rejection in organ transplantation by inhibiting effector T cell activation, promoting immune tolerance, and reducing reliance on long-term immunosuppressants while limiting the expansion of allogeneic-specific T and B cells.^342,343^ Tregs create an immunosuppressive environment by reducing IL-2 consumption and side effects. Short-term IL-2/anti-IL-2 antibody pre-conditioning promotes long-term acceptance of mismatched lung allografts by inducing Tregs to form protective clusters, eliminating the need for continuous immunosuppression.^344^ CAR-Treg therapy, especially when combined with rapamycin, prolongs survival in heart transplants and reduces the need for long-term immunosuppression.^345^ Hence, Treg-based therapies, including ex vivo expansion and reinfusion, are under investigation.^346^ However, successful Treg engraftment requires creating a suitable environment, overcoming host Treg competition, and ensuring IL-2 and antigen availability.^347^ Future research with larger sample sizes and standardized methods is needed to fully evaluate the efficacy and safety of Tregs in solid organ transplantation and establish consistent clinical guidelines.

#### Hematopoietic stem cell transplantation

Allogeneic hematopoietic stem cell transplantation (allo-HSCT) is an effective treatment option for numerous types of cancers. Tregs can suppress GVHD without affecting the effect of graft-versus-leukemia (GVL), and a decrease in the number of Tregs is associated with an increase in the severity of GVHD.^348,349^ Interventions such as ultra-low doses of IL-2 or adoptive transfer of Tregs can increase the Treg-to-effector T cell ratio and improve GVHD outcomes in animal models. These methods have been evaluated in phase I clinical trials.^350,351^ Research shows that Tregs limit the expansion of Tconvs involved in GVHD by shifting their metabolism from glycolysis to oxidative phosphorylation, reducing GVHD severity without impacting the graft-versus-tumor effect. Careful attention to timing and the Treg-to-Tcon ratio is crucial in infusion therapy.^352^ In an immunodeficient non-obese diabetic/severe combined immunodeficient (NSG) mouse model of allogeneic hematopoietic stem cell transplantation, Allan Thiolat et al. studied two IL-2/anti-IL-2 complexes (IL-2Cxs) and their effects on GVHD, tumor recurrence, and immune cell function. Both complexes prevent GVHD and promote Treg expansion, but only IL-2/anti-IL-2Cx, which increases Tregs, reduces CD8⁺ T cell exhaustion and has strong antitumor effects.^353^ Furthermore, metabolism plays a pivotal and fundamental role in the differentiation processes of both T cells and Tregs: glycolysis drives GVHD in allogeneic T cells, whereas oxidative phosphorylation regulates Treg inhibitory function.^354^

## Immunotherapy strategies targeting Tregs in human diseases

### Treg cell therapy

As of March 2025, ClinicalTrials.gov has witnessed the registration of a significant number of clinical trials leveraging Treg cells as an intervention, with more than 260 items in total. These trials included all study statuses and span a diverse range of human diseases, highlighting the growing interest in exploring the potential of Treg cell therapy. Among them, a total of 84 clinical trials are related to transplantation, 74 are associated with tumor specificity, 60 are targeted at autoimmune diseases, 31 concern metabolic diseases, 19 are related to cardiovascular diseases, and 12 are associated with neurodegenerative diseases (Table 2). Furthermore, most clinical trials employing Treg cell therapy have opted for ex vivo-expanded polyclonal Tregs. In contrast, antigen-specific Tregs are preferentially located at antigen-presenting sites and exhibit stronger immunosuppressive effects than polyclonal T cells.^355^ Therefore, efforts have focused on the use of genetic engineering techniques to express recombinant TCRs or synthetic receptors to manipulate the antigen specificity of Tregs, which appear to be more promising and feasible approaches.^356^ The purpose of Treg cell therapy in autoimmune diseases is to restore immune tolerance and mitigate pathological immune responses.^357^ Transplantation aims to prevent graft rejection and manage GVHD.^358^ In cancer, the primary goal is to modulate the immunosuppressive microenvironment and enhance antitumor immunity.^359^ Overall, Treg cell therapy represents a fascinating and rapidly evolving area of research, with the potential to revolutionize the treatment of numerous human diseases by capitalizing on the unique properties of these regulatory immune cells.

**Table 2:** Ongoing clinical trials of Treg cells and/or therapeutic agents are being conducted for human diseases. (Selected examples)

Intervention/Treatment	Disease types	Purpose	Identifier	Trial phase
Umbilical Cord Blood (UCB)-Treg + Liraglutide	Autoimmune Diabetes	The purpose of this study is to examine the safety as well as the therapeutic effect of ex-vivo expanded umbilical cord blood regulatory T cells when used in combination with Liraglutide in the treatment of autoimmune diabetes	NCT03011021	I/II
CD4^LVFOXP3	Immune dysregulation, Poly endocrinopathy, Enteropathy, X-linked (IPEX)	To test the feasibility of manufacturing and the safety of administering CD4^LVFOXP3 in up to 30 evaluable human participants with IPEX, and to evaluate the impact of CD4^LVFOXP3 infusion on the disease	NCT05241444	I
UCB-Treg	Autoimmune Diabetes	The purpose of this study is to examine the safety and the therapeutic effect of regulatory T cells from ex-vivo expanded umbilical cord blood in the treatment of autoimmune diabetes	NCT02932826	I/II
Infusion of autologous human polyclonal Treg cell	Amyotrophic lateral sclerosis, Multiple System Atrophy (MSA), Alzheimer’s Disease	To evaluate the safety and efficacy of injecting autologous human polyclonal regulatory T cells (NP001 cell injection) in patients with neurodegenerative diseases such as ALS, MSA, and AD	NCT06671236	I
RAPA-501 autologous T cells	Amyotrophic Lateral Sclerosis	The purpose of the RAPA-501-ALS study is to conduct a phase 2/3 expansion cohort study to evaluate the RAPA-501 autologous hybrid TREG/Th2 cells in patients with amyotrophic lateral sclerosis (pwALS)	NCT04220190	II/III
Infusion of TRK-001	Kidney transplantation	To determine the safety and efficacy of administering expanded regulatory T cells (TRK-001) to prevent allograft rejection in living donor renal transplant recipients	NCT06552169	II
Infusion of Treg cell	Heart transplantation	To develop a protocol for isolating Treg cells (thyTreg) from discarded thymic tissue in pediatric cardiac surgeries and then conduct a phase I/II clinical trial after pre-clinical studies to test the safety and efficacy of adoptively transferring autologous thyTreg for preventing rejection in children undergoing heart transplants	NCT04924491	I/II
Infusion of recipient Treg cell	Islet transplantation	The purpose of this clinical trial is to evaluate whether patients with brittle type 1 diabetes can achieve better blood sugar control after islet transplantation when receiving one of two types of immune cells (either their own regulatory T cells or immune cells from the islet donor’s bone marrow) along with the islet transplant. It also aims to assess the feasibility and safety of combining an islet transplant with the recipient’s Tregs or deceased donor bone marrow cells in these patients	NCT05973734	I
Infusion of Treg cell	Advanced Hematologic Cancer	Studying the side effects and best dose of donor Treg cells after an umbilical cord blood transplant in treating patients with advanced hematologic cancer or other disorder	NCT00602693	I
DLI-Boost	Hematological Malignancies	Show the advantage of Treg-depleted donor lymphocyte infusion (DLI) over standard DLI for treating relapse in blood cancers after HSCT	NCT03236129	III
Infusion of Treg cell	Relapsed/refractory and Ultra-high-risk AML and/or MDS	Whether the IS-free Treg-cell graft-engineered haplo HSCT approach will reduce risk of relapse while preventing usual toxicities related to stem cell transplants	NCT04678401	I
ILDTreg2	Hematological Malignancies	To enhance anti-tumor immune responses by selectively depleting Treg using CD127 as a marker in the context of DLI for relapsing hematological malignancies after allogeneic HSCT	NCT06180499	I/II
Infusion of depleted Treg cell	Relapsed Hematologic Malignancies	Evaluate the safety and efficacy of CD25^+^Treg depletion in DLI products using CliniMACS for optimal dosing in post-transplant relapsed hematologic malignancy patients	NCT00675831	I
Infusion of Treg cell	Myeloid Leukemia, Lymphoma, MDS	Assess the efficacy, safety, and feasibility of various Tcon and Treg dose combinations in HCT patients with HLA-matched donors using T cell-depleted grafts	NCT01660607	I/II
High dose irradiation conditioning + Treg/Tcon	Acute Myeloid Leukemia Acute Lymphoid Leukemia	Determine if hyper-fractionated TBI or TMLI combined with Treg/Tcon immunotherapy enhances cGvHD/disease-free survival post-HSCT in high-risk acute leukemia or other hematologic malignancy patients	NCT03977103	II
Infusion of Treg cell	Hematologic Malignancies	Estimate the rate of grade II-IV acute GVHD after infusing tTreg cells at a fixed ratio to the total CD3^+^ cell count from two UCB grafts in double UCB transplant recipients	NCT02118311	II
Infusion of Treg cell	aGVHD after UCB HSCT for Heme Malignancies	Adoptive transfer of T regulatory cell for suppression of acute graft-vs-host-disease after an umbilical cord blood transplant for hematologic malignancies	NCT02991898	II
Selectively depleting Treg cell	Advanced Metastatic Colorectal Cancer	Controlled and selective depletion of regulatory T-cell for cancer treatment, efficacy and safety study	NCT00986518	I/II
Infusion of Treg cell	Hematologic Malignancies	Determine the optimal doses of UCB Treg and CD3^+^ Teff cells to be infused, with the objective of maximizing the number of CD3^+^ Teff cells while avoiding grade II-IV acute GVHD	NCT01163201	I/II
Infusion of Treg cell	High-Risk Leukemia or other Hematologic Diseases	To evaluate the safety and determine the optimal dose of umbilical cord blood T-regulatory cell infusion followed by donor umbilical cord blood transplant in patients with high-risk leukemia or other hematologic diseases	NCT00376519	I
CAR19-tTreg	Lymphoma Leukemia	To evaluate the safety and potential effectiveness of allogeneic CAR19-tTreg in adults with relapsed/refractory (R/R) CD19^+^ B Acute Lymphocytic Leukemia (B-ALL), specifically to manage immune-related side effects from CD19-targeted therapies like CAR T-cell therapy, by modulating immune responses that may cause cytokine release syndrome or other toxicities	NCT05114837	I/II
Recombinant WT1ASCI+ Infusion of Treg depleted T Cells	Adult WT1 Acute Myeloid Leukemia	To evaluate the safety and the efficacy of combined treatment strategy of WT1ASCI, infusion of ex vivo regulatory T cells depleted T lymphocytes and in vivo regulatory T cells depletion as post-consolidation therapy in patients with WT1-positive Acute Myeloid Leukemia	NCT01513109	I
Infusion of Treg cell	Hematologic Malignancies	A feasibility trial of post-transplant Infusion of allogeneic regulatory t cells and allogeneic conventional T cells in patients with hematologic malignancies undergoing allogeneic myeloablative hematopoietic cell transplantation from haploidentical-related donors	NCT01050764	I/II
CD19/CD22-CAR-T	Adults With B-cell Acute Lymphoblastic Leukemia	To assess the safety of administering allogenic, donor-derived CD19/CD22-CAR T cells that meet established release specifications in adults with B-cell ALL following a myeloablative conditioning regimen and Orca-T to determine if this will augment graft versus leukemia without increasing acute GVHD or graft failure	NCT05507827	I
Infusion of Treg cell	Hematologic Malignancies	Compare Orca-T (“Orca-T”, a T-cell-depleted graft with additional infusion of conventional T cells and regulatory T cells) to standard care in patients undergoing MA-alloHCT for blood cancers, focusing on safety and efficacy	NCT05316701	III
Infusion of Treg cell	Hematologic Malignancies	Assess the safety, tolerability, and effectiveness of Orca-T, an engineered donor graft with added T cells, in patients receiving allogeneic stem cell transplants for blood cancers	NCT04013685	I

### Targeting Treg suppressor molecules

**Fig. 6:**
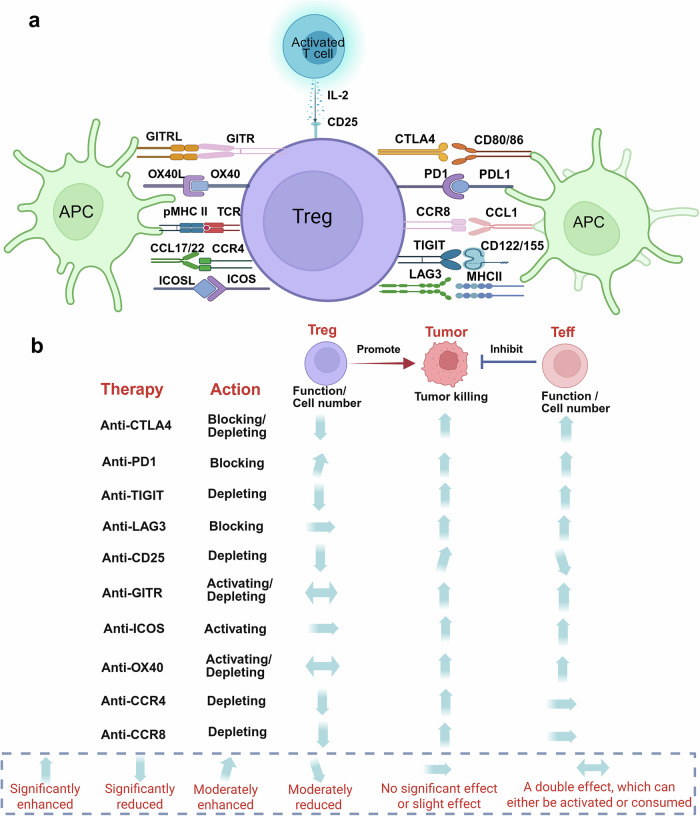
Targeting Treg suppressor molecules. **a** Listed are inhibitory and stimulatory antibodies that target Tregs. **b** Monoclonal antibody molecules that target Tregs to improve antitumor efficacy. This figure was created with Biorender.com

Blocking CD28/CTLA-4, PD-1, TIGIT and LAG-3 with monoclonal antibodies or activating CD25, GITR, ICOS, and OX40 can diminish the immunosuppressive function of Treg cells (Fig. 6). These approaches not only enhance antitumor immune responses but also hold therapeutic potential for managing autoimmune diseases,^360^ combating chronic viral infections,^361^ alleviating inflammatory conditions,^362^ and promoting anti-graft immune responses in transplantation settings.^363^ In autoimmune diseases, Treg cells often present functional abnormalities, and the immune balance is disrupted. Blocking the relevant targets mentioned above with monoclonal antibodies can weaken the abnormal immunosuppression of Tregs and appropriately regulate the function of effector T cells, which helps to rebalance the body’s immune state, reduce inflammation and damage, and relieve the condition of the disease.

#### CTLA-4 and PD-1

Tregs express immune checkpoints such as CTLA-4 and PD-1, suppressing immunity via ligand interactions with effector T cells.^364^ CTLA-4 blockade induces CD28-dependent hyperproliferation of Tregs in the TME, necessitating simultaneous Treg inactivation for tumor rejection. Disruption of this CTLA-4/CD28-dependent loop may reduce therapeutic benefits.^365^ Tregs also suppress T cells via CTLA-4-mediated trogocytosis, reducing CD80/CD86 expression. Combining CTLA-4 and PD-1/PD-L1 blockade enhances antitumor immunity.^366^ CTLA-4 also inhibits Treg glycolysis, and its blockade promotes glucose metabolism in tumors with low glycolytic activity.^367^ In mice, anti-CTLA-4 antibodies deplete TI-Tregs via Fc receptor interactions,^368^ although human Treg depletion remains debated.^369^ Ipilimumab depletes Tregs, whereas tremelimumab increases effector T cells without altering Treg levels. Both enhance intratumoral CD4^+^ and CD8^+^ infiltration, and Fc modifications may improve Treg depletion.^370,371^ Targeting OX40, CTLA-4, and GITR preferentially modulates intratumoral Tregs, especially in mouse models.^372^ Blocking CD28/CTLA-4 shows promise in autoimmune diseases,^373^ chronic viral infections,^374^ and transplantation.^375^ CTLA-4 regulates immune responses in MS and SLE.^376^ Abatacept, a CTLA-4 fusion protein, inhibits CD80/CD86-CD28 binding, reducing T cell activation in refractory rheumatoid arthritis,^377^ whereas belatacept improves kidney graft survival by blocking CD28 co-stimulation.^378^

PD-1 blockade can increase the number of suppressive Tregs in the TME, promoting tumor progression.^379^ In advanced gastric cancer, it enhances Treg function and is correlated with rapid progression.^380^ While PD-1 blockade boosts CD8^+^ T cell proliferation and cytokine production, its effectiveness depends on the CD8^+^ Teff-to-Treg balance.^381^ In highly glycolytic tumors such as MYC-amplified and liver cancers, Tregs show increased PD-1 expression. In low-glucose TMEs, Tregs absorb lactic acid via MCT1, which upregulates PD-1 by promoting NFAT1 translocation.^382^ αPD-1/PD-L1 treatment affects Tregs systemically, with tumor-draining lymph nodes crucial for response. Increased Treg activity post-treatment is linked to hyperprogression, and the pre-treatment PD-1 balance between CD8^+^ T cells and Tregs predicts efficacy.^383^ The PD-1 pathway plays diverse roles in Treg function, suggesting its therapeutic potential in autoimmune diseases.^384^ In transplantation, PD-1 maintains tolerance by limiting Teff activity and promoting Tregs, while its blockade may cause rejection. In GVHD, PD-1 inhibits donor T cells to reduce damage, but its blockade may exacerbate GVHD while enhancing antitumor immunity.^385,386^

However, the mechanisms of these antibodies remain unclear. Differences in their effects may stem from differences in Treg numbers, genetic backgrounds, and immunotherapy timing. Overall, the effects of ICBs on Tregs are influenced by metabolic substrates, co-stimulatory signals, Fc receptor-expressing phagocytes, and the timing of analysis.^387,388^

#### TIGIT

TIGIT has gained recognition as a potential therapeutic target in both autoimmune diseases and cancer.^389^ In conditions such as RA and SLE, where the immune system attacks the body, TIGIT regulates immune balance. Mouse models have shown that TIGIT deficiency or blockade leads to excessive T cell activation, inflammation, and increased autoimmune susceptibility, while activating TIGIT alleviates disease symptoms.^16,390^ Additionally, dexamethasone-loaded nanoparticles (IM-MNPs/DXM) targeting CD4^+^ T cells can regulate PD-1/TIGIT signals, suppress effector T cells, enhance Tregs, and reduce cytokine production.^391^ These nanoparticles show significant efficacy in treating lupus nephritis with few side effects, suggesting that they are promising treatment platforms.

TIGIT is highly expressed on TI-Tregs, and targeting it with antibodies depletes TI-Tregs without affecting Tconvs, making it a promising immunotherapy target.^392^ In melanoma patients, Tregs exhibit increased TIGIT expression and a higher TIGIT/CD226 ratio, which is associated with a greater Treg frequency in tumors and a poor response to ICB therapy.^393^ Targeting both TIGIT and CD226 activation may counteract Treg suppression in solid tumors.^394^ TIGIT^+^ Tregs suppress Th1 and Th17 cells, whereas TIGIT-deficient Tregs enhance antitumor immunity.^395^ Additionally, the balance between PD-1 and TIGIT in Tconv cells is crucial for antitumor function.^396^ Ongoing clinical trials, including one combining tiragolumab (anti-TIGIT) with atezolizumab (anti-PD-L1) for non-small cell lung cancer, highlight the therapeutic potential of TIGIT.^397^ However, the failure of the Skyscraper 06 study (Link: Genentech: Press Releases) means that the combination therapy did not demonstrate the expected advantage in extending survival in individuals afflicted with non-squamous non-small cell lung cancer, although the overall safety profile was in line with that of previous studies. In addition to the failure of the Skyscraper 06 study, Roche’s other important study, the Skyscraper 02 of Tilacumab, a phase 3 clinical trial in extensive-stage small cell lung cancer (ES-SCLC), also failed to meet the primary endpoints of PFS and OS.^398^ These setbacks undoubtedly cast a shadow on the prospects for the development of TIGIT monoclonal antibodies. Nevertheless, other TIGIT monoclonal antibodies are still being evaluated in clinical trials for the treatment of refractory solid tumors, either alone or in combination with other drugs (such as those in trials with NCT numbers: NCT05706207, NCT05390528, NCT05102214, NCT05060432, NCT05329766, NCT05390528, and NCT04354246).

#### LAG-3

LAG-3 and Treg cells form a complex, powerful immunosuppressive mechanism that aids tumor immune evasion and is associated with various diseases beyond cancer.^399,400^ LAG-3 is expressed primarily on B cells, NK cells, DCs, activated T cells, and Tregs.^401^ LAG-3 also suppresses Teff cell proliferation and cytokine production while promoting the expression of immunosuppressive factors such as IL-10 and TGF-β in Tregs.^102,402^ Blocking LAG-3 with monoclonal antibodies, such as relatlimab (combined with nivolumab as Opdualag), reduces Treg immunosuppressive functions and enhances T cell activation (NCT03470922). Additional clinical trials, such as NCT05352672, NCT01968109, NCT03459222, NCT05002569, and NCT04140500, are currently assessing the efficacy of anti-LAG-3 monoclonal antibodies in cancer treatment. Furthermore, combining LAG-3 blockade with PD-1/PD-L1 and CTLA-4 inhibition has shown promise in enhancing antitumor responses.^403^

Current research focuses on the role of LAG-3 in the tumor microenvironment, but its relationship with autoimmune diseases remains unclear. LAG-3 is critical for maintaining immune tolerance and may become a therapeutic target for autoimmune disorders.^404^ It plays a role in diseases such as T1D^405^ and MS,^406^ where it helps regulate Treg cells and prevents excessive immune activation. Reduced LAG-3 expression in autoimmune conditions such as relapsing-remitting MS and type 1 diabetes leads to dysregulated T cell responses, and enhancing LAG-3 activity could offer therapeutic potential.^405^ A GWAS meta-analysis linked 290 sequence variants to autoimmune thyroid disease (AITD), including a rare LAG3 variant (rs781745126-T) that reduces LAG-3 expression and is associated with AITD, vitiligo, and other T-cell-mediated diseases.^407^ This study suggested that LAG-3 deficiency or blockade enhances T cell activation and plaque inflammation in atherosclerosis, although its impact on lesion size needs further investigation in patients treated with LAG-3/PD-1 blocking antibodies for cardiovascular events.^400^

#### CD25

CD25, a component of IL-2R, is crucial for immune cell activation and is highly expressed in hematological malignancies such as leukemia and lymphoma. Targeting CD25 depletes Tregs, enhancing antitumor immunity. Modified IL-2 improves vascular leakage syndrome treatment by expanding cytotoxic T cells while reducing Tregs.^15^ Low-dose IL-2/CD25 in female NOD mice prevents the progression of pre-T1D and improves β-cell function after treatment cessation.^408^ It also enhances antitumor immunity by activating Tregs and increasing the number of effector T and NK cells.^409^ Additionally, mutant IL-2 and receptor-biased IL-2 are being explored for the treatment of autoimmune diseases and tumors in clinical trials.^410^ Roche’s RG6292, a non-blocking anti-CD25 antibody, selectively eliminates Tregs without affecting IL-2 signaling in Teff cells (NCT04158583), resulting in limited toxicity in animal models. Antibody‒drug conjugates such as ADCT-301, which target CD25, show enhanced safety and efficacy in treating CD25^+^ lymphoma, suggesting potential for clinical application. Further research should focus on targeted antibody activity at tumor sites and engineering approaches to improve efficacy and reduce toxicity.

Targeting CD25 for cancer immunotherapy focuses on depleting Tregs, but its lower expression in solid tumors limits its effectiveness.^411^ Additionally, the therapeutic window for CD25-targeted immunotherapy is narrow because CD25 is not only a marker for Tregs but also a molecule upregulated on activated Teffs. Studying CD25-targeted drugs in CD25^+^ tumors requires a balance of binding affinities to both Tregs and Teffs for effective treatment, as Treg clearance is accompanied by Teff clearance. The dosage and timing of these drugs are also critical to treatment outcomes.^412,413^ Moreover, combining Fc-optimized anti-CD25 antibodies with anti-PD-1/PD-L1 antibodies has synergistic effects in the treatment of mouse tumor models.^414^

#### GITR

GITR, a co-stimulatory molecule on activated CD4^+^ and CD8^+^ T cells and Tregs, is a promising immunotherapy target because of its ability to enhance Teff function and counteract Treg suppression.^415^ In autoimmune diseases, it can suppress effector T cell activation and enhance Treg-mediated immune tolerance to reduce inflammation in diseases such as systemic lupus erythematosus and multiple sclerosis.^416,417^ However, in specific autoimmune disease models, such as EAT^418^ and EAE,^419^ anti-GITR antibodies can worsen disease.

GITR activation increases IL-2, CD25, and interferon gamma production, leading to antitumor effects by reducing Foxp3^+^ Treg functions. The GITR agonist DTA-1 can reduce intra-tumoral Tregs by more than 50%.^420,421^ A phase 1 trial of the GITR agonist TRX518 (NCT01239134) revealed safe and immune effects in advanced cancer patients, reducing Tregs both systemically and within tumors but with limited clinical response. Preclinical studies suggest that combining GITR agonism with PD-1 blockade may rejuvenate T cells.^422^ This has prompted research on combining TRX518 with PD-1 pathway blockade for treating patients with advanced refractory tumors (NCT02628574). GITR has shown efficacy in mouse models but not in clinical trials, potentially owing to differences in GITR expression between humans and mice.^423^ GITR^+^CD4 T cells, including Tregs and Tconv cells, contribute to the immunosuppressive microenvironment in gastric cancer (GC), and their enrichment is linked to poorer prognosis. In addition, the cellular distribution of the GITR protein in Treg cells depends on the microenvironment.^424^ Preclinical studies have shown that combining GITR agonism with Fc-γ receptor-mediated Treg depletion increases the Teff: Treg ratio, leading to tumor shrinkage.^425^ Targeting GITR in Tregs with an αGITR antibody promotes their differentiation into effector T cells, reducing Treg-mediated suppression and boosting antitumor immunity in glioblastoma. Combined with αPD1 antibodies, this approach improves survival and may lead to complete tumor eradication, offering a promising treatment strategy.^100^

#### OX40

OX40 is a co-stimulatory molecule in the TNF superfamily that facilitates T cell activation via TCR stimulation^426^ and is expressed in multiple T cell subsets, including effector T cell and Tregs.^427^ Binding OX40 to its ligand increases the survival and expansion of Teff and memory T cells while reducing the immunosuppressive activity of Tregs. OX40 signaling is involved in the activity or recurrence of multiple autoimmune and inflammatory diseases, such as rheumatoid arthritis and myasthenia gravis.^428,429^ The OX40/OX40L axis also contributes to skin inflammation in diseases, including atopic dermatitis (AD), psoriasis, alopecia areata (AA), and pemphigus,^430^ making it a promising therapeutic target. The OX40 agonist OX86 induces Treg depletion in the TME and increases CD8^+^ Teff infiltration in a B16F10 melanoma model. OX40 co-stimulation counteracts Treg suppression and enhances T cell survival, proliferation, and effector functions.^431^ MEDI6383, a recombinant OX40L IgG4P Fc fusion protein, shows potent agonist activity, counters Treg suppression, enhances tumor-reactive T cell cytolytic activity, and reduces tumor growth in mice.^432^ OX40 agonists have demonstrated enhanced antitumor immune capabilities in various tumor models and are currently being explored as novel targets for cancer immunotherapy in clinical trials. The primary OX40 agonist antibodies used in clinical research include INCAGN01949, MEDI6469, BAT6026, PF04518600, and SL279252.^433–436^ An innovative approach using nanoparticles loaded with an OX40L plasmid reprograms tumor cells into antigen-presenting cells, triggering T cell proliferation and antitumor responses without significant toxicity.^437^ While OX40 therapy shows promise in preclinical models, its clinical efficacy alone is moderate, but combining OX40 therapy with anti-PD-1/PD-L1 inhibitors has strong potential.^438^

#### ICOS

ICOS belongs to the CTLA-4 family and is widely expressed in activated CD4^+^ T cells and CD4^+^ iTregs. It enhances T cell activation and proliferation by interacting with its ligand, ICOSL. ICOS expression is elevated in various autoimmune disorders, allergic conditions, and cancers. For example, ICOS⁺ Tregs are the key cells for preventing diabetes in pancreatic islets,^439^ and expanding these anti-inflammatory cells can effectively treat diabetes. ICOS⁺ regulatory T cells but not ICOS-deficient T cells can improve active colitis.^440^ Emerging evidence suggests that IL-35 secreted by ICOS⁺ Tregs can inhibit IL-17-related airway hyperresponsiveness, which acts synergistically in allergic asthma.^441^ However, intervention in the ICOS pathway at different time points during the course of asthma can lead to different outcomes. When drugs or other methods are used to disrupt ICOS signaling for treatment, attention should be given to the intervention time and a comprehensive understanding of the specific disease.^442^ Activation of the ICOS signaling pathway during tumor immunotherapy can enhance antitumor immune responses.^443^ The ICOS regulates the function of effector T cells, particularly by promoting the differentiation of Th1 and Th17 cells, thereby enhancing their ability to attack tumor cells.^444^ Research indicates that GC patients with a greater proportion of ICOS^+^ Tregs have a poorer prognosis, whereas patients with a lower proportion of ICOS^+^ Tregs tend to have a better prognosis.^445^ Moreover, studies have reported that ICOS^+^ Tregs are the primary immunosuppressive cells in the liver cancer microenvironment.^368^ Therapies that target ICOS, such as JTX-2011, KY1044, and GSK609 (NCT02904226, NCT03829501, and NCT02723955), are approved by the FDA or are being evaluated in clinical trials. Other studies have shown that the antagonist MEDI-570 and agonist JTX-2011 (vopratelimab) can reduce the frequency of intra-tumoral Tregs in mice without affecting the frequency of pTregs.^446^

### Inhibiting the infiltration of Treg cells

Tumor and stromal cells promote the accumulation of Tregs, including CCL22-CCR4, CCL1-CCR8, and CCL28-CCR10, through chemokine‒receptor interactions. These interactions drive Treg migration in the tumor microenvironment. Antibody therapy is a strategy to prevent Treg infiltration. CCR4 is key for Treg homing to tumors and is influenced by BRAFV600E signaling in melanocytes.^447^ Mogamulizumab, an anti-CCR4 antibody, reduces CCR4^+^ Tregs, enhancing antitumor responses in cutaneous T-cell lymphoma.^448^ In the tumor microenvironment, CCL17 and CCL22 recruit Tregs via CCR4, promoting immune escape. Targeting CCR4 with an antagonist in Pan02 tumor-bearing mice blocked Treg invasion and boosted tumor immunity.^449,450^ Notably, tumors with low initial CCL17 and CCL22 levels presented increased expression of these chemokines and CCR4^+^ TI-Tregs when treated with anti-CTLA-4, and combined blockade of CTLA-4 and CCR4 significantly prevented tumor development.^244^ Monitoring CCL17/CCL22 levels is crucial to avoid the counterproductive effects of immunotherapies such as anti-CTLA-4. For example, blocking CCL1, CCL22, CCR4, and CCR8 can prevent Treg migration.

CCR8 appears to be non-essential for TI-Treg migration, as CCR8-deficient Tregs have similar tumor invasion rates to wild-type Tregs. Although there is no immunosuppressive effect, CCR8 binds to CCL1 to promote Foxp3 transcription and enhances the stability of TI-Tregs.^451^ Anti-CCR8 combined with anti-PD-1 therapy has synergistic effects, although caution is needed, especially in atherosclerotic plaques. In breast cancer, tumor-resident Tregs upregulate CCR8, making CCR8 targeting a promising immunotherapy strategy.^452^ IPG7236, a selective CCR8 antagonist, has strong anticancer effects, modulates Tregs and cytotoxic T cells, and significantly suppresses tumors in a breast cancer mouse model, both alone and with anti-PD-1.^453^ The CCL28-CCR10 system is less prominent in TI-Tregs but plays a significant role in ovarian cancer, where CCR10^+^ TI-Tregs respond to hypoxia-induced CCL28 and secrete VEGFA to increase immune tolerance and angiogenesis.^454^ In colorectal cancer, CCL28 overexpression is correlated with poor prognosis, as its interaction with CCR10 recruits Tregs, promoting tumor progression, indicating that removing CCR10^+^ Tregs from the TME could be a potential treatment for colorectal cancer.^455^

### Epigenetic drugs

Epigenetic modifications such as histone and DNA methylation, acetylation, and ubiquitination regulate Foxp3 expression and the transcriptional profile of Tregs.^456^ Researchers are investigating epigenetic modifications to selectively modulate Treg function, converting them into cells with lower suppressive or even pro-inflammatory properties rather than depleting Tregs. This approach aims to harness the immunoregulatory properties of Tregs while mitigating their negative effects on antitumor immune responses.^115^

UHRF1, a DNMT adapter protein, is a potential therapeutic target; its deficiency enhances iTreg function in colitis,^457^ whereas its overexpression may impair Tfh cell differentiation, potentially contributing to systemic lupus erythematosus.^458^ EZH2, a PRC2 catalytic subunit with H3K27me3 activity, is upregulated in Tregs upon CD28 costimulation and is essential for immune tolerance.^459^ Its inhibition suppresses Tregs, enhancing antitumor immunity, whereas its activation promotes tolerance in autoimmune and transplant settings.^460,461^ Disruption of EZH2 shifts TI-Tregs to a pro-inflammatory state, increasing Teff activity and remodeling the TME.^462^ In combination with CTLA-4 blockade, EZH2 inhibitors enhance antitumor responses.^463^ Clinical trials are exploring the combination of the EZH2 inhibitor tazemetostat with ICB for lymphomas and solid tumors (NCT05152459, NCT04762160). The 18 types of histone deacetylases (HDACs), which are classified into five classes, regulate Foxp3 expression. HDAC9 is crucial for Foxp3 inhibition, whereas class I HDAC inhibitors (HDAC1, HDAC3, and HDAC8) modulate Treg function.^464,465^ For example, entinostat reduces Foxp3 expression, suppresses Treg activity, and enhances anticancer therapy via STAT3 activation.^466^ Inhibiting HDAC11 increases OX40L expression in Hodgkin lymphoma cells, shifting cytokine secretion to promote Th1/Th17 responses.^467^ Class IIa HDACs act primarily through protein interactions and can be targeted to disrupt Foxp3^+^ Treg function, enhancing anticancer immunity.^468^ The HDACi STR-V-53 shows promise in hepatocellular cancer treatment when combined with sorafenib or anti-PD1 therapy.^469^ HDAC inhibitors can also promote Treg production and tolerance, suggesting therapeutic potential for inflammatory diseases and organ transplantation.^470^ CBP/p300 regulates Treg differentiation by modulating pro-inflammatory cytokine secretion. During pTreg development, p300 inhibition blocks TGF-β-mediated CNS1 acetylation without affecting *Foxp3* promoter acetylation.^471^ In murine models of lung cancer and mesothelioma, p300 inhibition reduces the number of intra-tumoral Tregs and enhances T cell infiltration, promoting anticancer immunity.^472^ GNAT family members (GCN5 and PCAF) also influence Treg function and antitumor immunity through partially unique and partially overlapping mechanisms. Additionally, histone acetyltransferases from the GNAT family differ from previously described Treg histone acetyltransferases (such as CBP, Tip60, and p300) in regulating Treg function and may be valuable therapeutic targets in immunooncology.^468^ Selective demethylation of the Treg-specific region stabilizes Foxp3 expression, and CNS2/STAT5 signaling promotes Treg stability, ensuring lineage identity in response to IL-2.^58^ CNS3, as an epigenetic switch, regulates the epigenetic state of the *Foxp3* promoter, ensuring that precursor cells differentiate into Tregs in response to TCR stimulation.^473^

### CAR-Tregs

Currently, promising approaches in Treg cell research include redirecting T cells through chimeric antigen receptor (CAR) constructs for reprogramming and utilizing CRISPR-Cas9 technology to edit Treg genes.^474,475^ Future Treg cell therapies will benefit from advancements in immuno-oncology, especially CAR T-cell therapy, with over 1,520 clinical trials conducted globally (ClinicalTrials.gov). Unlike TCR Tregs, CAR-Tregs function independently of MHC and have a reduced reliance on IL-2, maintaining their phenotype while exhibiting stronger suppressive efficacy and enhanced tissue-homing capabilities.^476,477^ The key advantage of CAR-Tregs over CAR-T cells lies in their immunomodulatory effects, particularly in controlling overactive immune responses, thus offering safer treatment by reducing immunosuppression and inflammation, such as cytokine release syndrome.^478^ CAR-Treg cells expressing Foxp^3+^CD25^High^ maintain a Treg-specific gene expression program with low cytotoxic potential and expand inversely to CAR-T cell proliferation in vivo, independent of tumor burden.^479^ Increased CAR-Treg levels in patients who fail CAR T-cell therapy hinder CAR-T cell expansion and contribute to late relapse.^480^ Modulating CAR-Treg cells may enhance antitumor effects or reduce neurotoxicity. New applications for CAR-Tregs are emerging, including B cell-targeting antibody receptor Tregs (BAR Tregs), which regulate FVIII-specific B cells to prevent or reduce anti-FVIII antibody production.^481^ Additionally, alloantigen-specific CAR Tregs targeting HLA-A2 significantly prolong heart transplant survival in mice, with enhanced efficacy when combined with rapamycin, suggesting the potential for reducing long-term immunosuppression in organ transplantation.^345^

The next generation of Treg cell engineering utilizes synthetic biology, offering systems such as T-cell antigen couplers, optimized CARs for soluble ligands, and the SUPRA CAR system to enhance specificity and address challenges such as tonic signaling and exhaustion. The SUPRA CAR requires multiple targets for activation, making it ideal for targeting tissue-specific antigens and solid tumors.^388^ In addition to oncology, CAR-Treg therapy shows promise for treating autoimmune^21,482^ and chronic inflammatory diseases, such as asthma,^483^ T1D,^484^ and MS.^485^ By refining CAR design for specificity, persistence, and safety, CAR-Treg therapy is advancing precision immunotherapy. In the future, CAR-Tregs are being developed with three functional domains, namely, sensing, activation, and effector functions, enhancing their capabilities beyond those of classical Tregs.^356^ Optimizing Treg cell engineering involves identifying signaling pathways that, when integrated into CAR-like receptors, ensure the stability, survival, and suppressive function of antigen-specific Tregs. Progress also depends on selecting the best targets, such as inflamed tissues, pathogenic T cells, or antigen-presenting cells. Strategies that target soluble antigens and combine CAR-Tregs with other therapies or chemotherapies should also be explored for enhanced efficacy.

### CRISPR-Cas9 editing

CRISPR-Cas9 enhances T cell function and enables precise therapeutic interventions by targeting oncogenes, tumor suppressors, and Tregs.^486^ It restores Treg accumulation in obesity by modifying IFNαr1 signaling^273^ and increases the number of Tregs to reduce GVHD in leukemia transplants.^487^ In cancer, CRISPR suppresses Treg-mediated inhibition, boosting immunity, whereas in autoimmune diseases, it enhances Treg activity to curb inflammation.^488^

CRISPR-Cas9 can be used in several ways to edit Treg cells for various diseases. First, gene knockout, in which specific genes (*e.g., Foxp3, IL-34, HO-1, BATF*)^488–490^ are knocked out in Treg cells, enhances tumor suppression, increases inflammatory sensitivity, and promotes cell death under oxidative stress. For example, knocking out *TGFBR2* in Mesothelin CAR-T cells increases IFN-γ and IL-2 production, inhibits Foxp3 upregulation, and improves tumor elimination in vivo.^491,492^ Knocking out *Rag-2* in mouse Tregs enhances the antitumor effect of PD-1 inhibitors in triple-negative breast cancer.^493^ Second, gene modification: CRISPR-Cas9 can modify genes in Tregs to enhance antitumor function. This includes altering Treg recognition of tumor antigens or engineering them to secrete cytokines such as IL-2 to support effector T cells.^494^ Third, genome editing: CRISPR-Cas9 can edit the entire genome of Tregs to modulate their immunosuppressive functions and insert specific antigen receptors to enhance antitumor responses.^495^ Engineered Tregs, combined with advancements in hypoimmunogenic human pluripotent stem cells, show significant antitumor effects and have the potential to revolutionize Treg cell therapy.

Efforts to improve CRISPR-Cas9 technology focus on reducing off-target effects and genomic alterations by engineering high-fidelity Cas9 versions and exploring alternative CRISPR‒Cas systems. CRISPR now extends beyond genome editing to include gene activation, DNA methylation, and RNA editing. This technology enables precise knock-in of antigen receptors and editing of genes regulating Treg function.^496,497^ Recent advancements, such as the use of polymer-stabilized Cas9 nanoparticles and modified repair templates, have improved editing efficiency and reduced toxicity, increasing success across various cell types.^498,499^ CRISPR screens are also used to identify gene pathways that regulate Foxp3 expression in Tregs. Combining CRISPR–Cas9 with high-throughput technologies and bioinformatics is crucial for optimizing Treg cell therapies and achieving immune tolerance in autoimmune diseases.

### IL-2

IL-2 is a pleiotropic cytokine that regulates immune homeostasis by balancing Teffs, NK cells, and Tregs. High-dose IL-2 enhances antitumor immunity but induces inflammation, whereas low-dose IL-2 selectively expands Tregs, promoting immune tolerance.^500^ To balance these responses, low-dose IL-2 and IL-2 derivatives have been developed to preferentially expand Tregs, promoting immune tolerance while minimizing off-target immune activation. Owing to the high-affinity expression of IL-2 receptors (CD25) on Tregs, low-dose IL-2 can selectively expand Tregs, showing promising potential in the treatment of various diseases, including autoimmune diseases (such as SLE,^501^ T1D,^502^ primary biliary cholangitis,^503^ and ulcerative colitis^504^), as well as certain skin conditions (such as alopecia areata^505^ and psoriatic arthritis^506^), and improving clinical symptoms. However, the efficacy and safety of this treatment depend on precise dose adjustments to avoid adverse effects on effector T and NK cells; furthermore, its use in solid organ transplants has not been well studied.^507^ IL-2 derivatives are designed to optimize bioactivity, half-life, selectivity, and safety, primarily for Treg expansion, Teff suppression, and improved pharmacokinetics. Bempegaldesleukin (NKTR-214), a PEGylated IL-2 derivative, preferentially activates CD122, promoting CD8^+^ T and NK cell expansion while limiting intratumoral Tregs, making it promising for tumor microenvironment modulation.^508,509^ IL-2/JES6 complexes and the single-chain hIL-2/F5111 fusion protein (F5111 IC) selectively expand Tregs, enhancing allograft tolerance and autoimmune therapy.^510^ Additionally, the mRNA-encoded IL-2 mutein (HSA-IL2m) expands Tregs and mitigates autoimmune disease in preclinical models, highlighting the potential of mRNA-based IL-2 immunotherapy.^511^

Moreover, the combination of nanocarriers and IL-2 provides a precise and efficient regulatory strategy for autoimmune diseases, gene therapy, and immune tolerance induction. Encapsulating IL-2 in nanoparticles or conjugating it to Treg-targeting molecules enhances its selective delivery, promoting Treg expansion, stability, and function, thereby improving immune regulation. This strategy has been validated across various nanoparticle platforms, including PLGA nanoparticles,^512^ ImmTOR nanoparticles,^513^ and AuNRs.^514^ These nanomaterials enable sustained IL-2 release, prevent non-specific activation of effector T cells, and enhance antigen-specific Treg expansion and functional durability while reducing dosage requirements and minimizing side effects.

### Effects of the gut microbiome on Treg function

The gut microbiome influences the function of Tregs, thereby affecting various immune-related diseases. By modifying the gut microbiota through dietary changes or fecal microbiota transplantation, Treg activity can be indirectly enhanced. This not only boosts antitumor immunity but also may improve the immune balance in autoimmune diseases, metabolic disorders, allergic reactions, cardiovascular diseases, etc.^515–518^ Studies have shown that alterations in the gut microbiota affect the Th17/Treg balance, influencing inflammatory bowel disease and myasthenia gravis.^519,520^ Modulating the gut microbiome enhances anti-PD-L1 efficacy by influencing Treg activity. Sivan et al. ^521^ reported that Bifidobacterium strains increase antitumor immunity and improve the anti-PD-L1 response. Specific gut bacteria also impact anti-PD-1 therapy in metastatic melanoma by regulating Treg function.^522^

### Approaches to expand Treg function during cancer immunotherapy

**Fig. 7:**
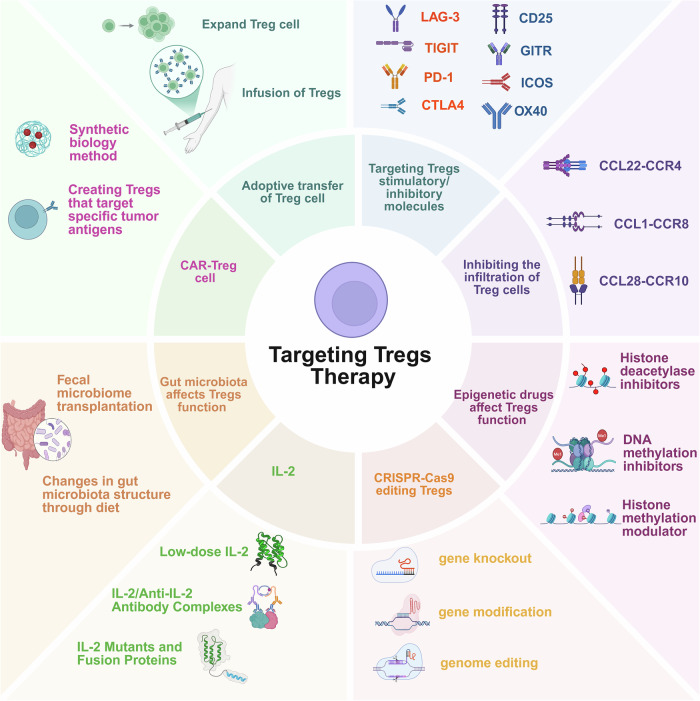
Treg cells exhibit a variety of targeting strategies in human diseases. The figure demonstrates the application of Treg cell therapies, such as the infusion of Tregs in diseases. Moreover, inhibitory molecules such as CTLA4 and stimulatory molecules such as GITR can enhance antitumor efficacy, and they have demonstrated potential application prospects in a variety of other diseases. Targeted modulation is achieved through pathways such as CCL22-CCR4, CCL1-CCR8, and CCL28-CCR10, as well as strategies for promoting the proliferation and function of Treg cells via the use of low doses of IL-2 and IL-2 derivatives. Additionally, the effects of the gut microbiome, epigenetic/histone deacetylase inhibitors, fecal microbiota transplantation, CRISPR–Cas9 gene editing, synthetic biology methods, and dietary changes that alter the structure of the gut microbiota on Treg function are also discussed. This figure was created with Biorender.com

Cancer immunotherapy approaches, including CTLA-4 and PD-1 blockade, often lead to the depletion of Treg cells throughout the body, accompanied by a series of side effects, such as significant autoimmune complications.^523–525^ Enhancing Treg function may be a therapeutic strategy to address this issue. As regulators of the immune system, Treg cells suppress excessive immune responses and contribute to the maintenance of immune homeostasis. Several approaches can be taken to enhance Treg cell function: (1) First, through specific drugs or treatment methods, Treg cells can be selectively activated or expanded, enabling them to exert stronger immune regulatory effects in the TME. Drugs, including the mTOR inhibitor rapamycin, as well as biological agents, such as IL-10, low-dose IL-2, and peptide, have been investigated for their potential in this regard.^526–528^ (2) Modulating the signaling pathways of Treg cells: By conducting in-depth research into the signal transduction mechanisms of Treg cells, we can identify key molecules or pathways that affect their function.^525^ By regulating these signaling pathways, we can precisely control the function of Treg cells and enable them to play a more active role in tumor immunotherapy. (3) In combination with other immunotherapy methods, by combining Treg cell enhancement strategies with other immunotherapy methods, such as antibody and cell therapies, synergistic effects can be achieved, improving treatment outcomes.^529^ In Treg cell therapy, the administration of Treg cells can restore immune tolerance by rectifying the Treg imbalance and directly increasing the number of Treg cells, thereby enhancing immune suppression. Overall, it is crucial to closely monitor potential side effects and complications during the treatment process and make timely adjustments and interventions. With further research and technological advancements, we believe that more effective treatment strategies will emerge in the future, resulting in better treatment outcomes and an improved quality of life for patients with cancer (Fig. 7).

## Conclusion and future perspectives

Typically, Treg cells are crucial for preserving immune tolerance and ensuring overall immune homeostasis. This overview systematically summarizes the characteristics, classification, function, and mechanism of Treg cells as well as Treg-targeting approaches in cancer immunotherapy. Tregs possess unique characteristics and functions to regulate immune responses through a series of complex mechanisms, including direct contact with other immune cells, secretion of inhibitory cytokines, and modulation of antigen presentation.^530,531^ These features endow Tregs with tremendous potential for the treatment of various diseases, especially in cancer immunotherapy.^529^ Currently, Treg cell research is exploring promising avenues, such as the following: (1) Treg cell therapies, such as engineered IL-2 formulations, improving the specificity of Treg-targeted interventions, which are being explored to treat autoimmune diseases, transplant rejection, and certain cancers by modulating immune tolerance and preventing excessive immune responses. These therapies aim to enhance graft survival and reduce inflammation without compromising immune defense; (2) Reprogramming T cells through CAR constructs, which involves redirecting them and editing the genes of Tregs via CRISPR-Cas9 technology; (3) Deleting or reducing Tregs in the tumor microenvironment can boost antitumor immunity, which can be achieved via monoclonal antibodies targeting Treg markers such as CTLA-4, PD-1, TIGIT, and CD25, and the immune checkpoint molecules OX40, GITR, and ICOS; and (4) Tumor and stromal cells secrete chemokines, promoting Treg accumulation at the tumor site via interactions such as CCL22‒CCR4, CCL1‒CCR8, and CCL28‒CCR10. Antibody therapy is a strategy to prevent Treg infiltration in the tumor microenvironment. (5) Epigenetic processes, including histone methylation, acetylation, ubiquitination, and DNA methylation, also target Treg function by controlling the expression of Foxp3. (6) Other pathways, such as lipid oxidation or the gut microbiome, can affect Treg activity and enhance antitumor immunity. With respect to their functions and mechanisms, Tregs perform mainly immunomodulatory functions by inhibiting the stimulation and proliferation of effector T cells and increasing the production of anti-inflammatory factors. Tregs perform these functions through various mechanisms, including cell‒cell contact inhibition, cytokine‒mediated inhibition, and metabolic intervention.^532,533^ These mechanisms collectively ensure the crucial role of Tregs in maintaining immune balance and preventing autoimmune diseases. Importantly, the role of Tregs in tumor immunotherapy is complex and context dependent. The analysis and identification of specific Treg subsets related to immunosuppression and therapeutic responses can guide personalized immunotherapy strategies. Understanding the heterogeneity and functional characteristics of Tregs in individual patients may help tailor treatment plans to improve therapeutic outcomes.

Although Treg cell therapy has immense potential, it has certain limitations. First, alterations in the number and functionality of Treg cells can result in the development of autoimmune diseases. In patients with untreated multiple sclerosis, active systemic lupus erythematosus, and active Crohn’s disease, the number of Treg cells is notably decreased, and their functionality is compromised. This reduces the efficiency of Treg cells in regulating immune responses, potentially contributing to excessive immune reactions and immune escape, which in turn affects treatment outcomes.^524^ Second, the simple infusion of autologous Treg cells often fails to achieve the desired therapeutic effect. Although Treg cells have immunosuppressive properties and can regulate immune responses, in some cases, functional disorders of Treg cells in patients may already occur, rendering them unable to effectively exert their effects.^534^ Additionally, although stimulating polyclonal Treg cells with antigen-presenting cells to obtain disease-related antigen-specific Treg cells is feasible, it is expensive, complex, and may yield insufficient cell numbers. Furthermore, in tumor therapy, Treg cells may increase the degree of immune evasion of tumor cells.^535^ Hence, the management and surveillance of Treg cell function in tumor therapy has emerged as a pivotal concern. To overcome these limitations, further research is needed to delve deeper into the biological characteristics and functional mechanisms of Treg cells, as well as to optimize treatment methods to improve therapeutic outcomes and reduce side effects.

We envision several exciting avenues for future research. For instance, gaining profound insights into the mechanisms governing Treg cell function and malfunction in autoimmune diseases is essential. Moreover, the optimization of Treg cell isolation, expansion, and delivery methods is essential. Advances in cell culture techniques and biomanufacturing processes can help overcome the challenges associated with obtaining sufficient numbers of functional Treg cells. Additionally, the development of novel delivery systems, such as encapsulation in biodegradable scaffolds or the use of cell-penetrating peptides, can enhance the efficiency of Treg cell engraftment and function in vivo. Future studies should concentrate on identifying the elements that control Treg cell count and activity, facilitating the development of specific approaches to rejuvenate or boost their inhibitory functions. In conclusion, despite these challenges, Treg cell therapy presents a promising future with the potential to significantly improve treatment effectiveness and safety.

## References

[1] Horwitz, D. A., Fahmy, T. M., Piccirillo, C. A. & La Cava, A. Rebalancing immune homeostasis to treat autoimmune diseases. *Trends Immunol.* **40**, 888–908 (2019). 31601519 10.1016/j.it.2019.08.003 PMC7136015

[2] Fontenot, J. D., Gavin, M. A. & Rudensky, A. Y. Foxp3 programs the development and function of CD4+CD25+ regulatory T cells. *Nat. Immunol.* **4**, 330–336 (2003). 28115587

[3] Hori, S., Nomura, T. & Sakaguchi, S. Control of regulatory T cell development by the transcription factor Foxp3. *Science* **299**, 1057–1061 (2003). 28115586

[4] Ichiyama, K. et al. Transcription factor Ikzf1 associates with Foxp3 to repress gene expression in Treg cells and limit autoimmunity and anti-tumor immunity. *Immunity* **57**, 2043–2060.e2010 (2024). 39111316 10.1016/j.immuni.2024.07.010

[5] Sakaguchi, S. Naturally arising CD4+ regulatory t cells for immunologic self-tolerance and negative control of immune responses. *Annu. Rev. Immunol.* **22**, 531–562 (2004). 15032588 10.1146/annurev.immunol.21.120601.141122

[6] Shevach, E. M. Regulatory T cells in autoimmmunity*. *Annu. Rev. Immunol.* **18**, 423–449 (2000). 10837065 10.1146/annurev.immunol.18.1.423

[7] Smigiel, K. S., Srivastava, S., Stolley, J. M. & Campbell, D. J. Regulatory T-cell homeostasis: steady-state maintenance and modulation during inflammation. *Immunol. Rev.* **259**, 40–59 (2014). 24712458 10.1111/imr.12170 PMC4083836

[8] McClymont, S. A. et al. Plasticity of human regulatory T cells in healthy subjects and patients with type 1 diabetes. *J. Immunol.* **186**, 3918–3926 (2011). 21368230 10.4049/jimmunol.1003099 PMC3091943

[9] Littman, D. R. & Rudensky, A. Y. Th17 and regulatory T cells in mediating and restraining inflammation. *Cell* **140**, 845–858 (2010). 20303875 10.1016/j.cell.2010.02.021

[10] Kasper, I. R., Apostolidis, S. A., Sharabi, A. & Tsokos, G. C. Empowering regulatory T cells in autoimmunity. *Trends Mol. Med.* **22**, 784–797 (2016). 27461103 10.1016/j.molmed.2016.07.003 PMC5003773

[11] Becker, M., Levings, M. K. & Daniel, C. Adipose-tissue regulatory T cells: critical players in adipose-immune crosstalk. *Eur. J. Immunol.* **47**, 1867–1874 (2017). 28849586 10.1002/eji.201646739

[12] Alfar, R. et al. Selective reprogramming of regulatory T cells in solid tumors can strongly enhance or inhibit tumor growth. *Front. Immunol.* **14**, 1274199 (2023). 37928524 10.3389/fimmu.2023.1274199 PMC10623129

[13] Glasner, A. et al. Conserved transcriptional connectivity of regulatory T cells in the tumor microenvironment informs new combination cancer therapy strategies. *Nat. Immunol.* **24**, 1020–1035 (2023). 37127830 10.1038/s41590-023-01504-2 PMC10232368

[14] Liang, Y. et al. Enhancing anti-tumor immune responses through combination therapies: epigenetic drugs and immune checkpoint inhibitors. *Front. Immunol.* **14**, 1308264 (2023). 38077327 10.3389/fimmu.2023.1308264 PMC10704038

[15] Peng, Y. et al. CD25: a potential tumor therapeutic target. *Int. J. Cancer* **152**, 1290–1303 (2023). 36082452 10.1002/ijc.34281

[16] Zhao, J. et al. TIGIT: An emerging immune checkpoint target for immunotherapy in autoimmune disease and cancer. *Int. Immunopharmacol.* **120**, 110358 (2023). 37262959 10.1016/j.intimp.2023.110358

[17] Tobin, J. W. D., Bednarska, K., Campbell, A. & Keane, C. PD-1 and LAG-3 checkpoint blockade: potential avenues for therapy in B-cell lymphoma. *Cells*. **10**, 1152 (2021). 10.3390/cells10051152 PMC8151045 34068762

[18] Harris, D. T. & Kranz, D. M. Adoptive T cell therapies: a comparison of T cell receptors and chimeric antigen receptors. *Trends Pharm. Sci.* **37**, 220–230 (2016). 26705086 10.1016/j.tips.2015.11.004 PMC4764454

[19] Sawitzki, B. et al. Regulatory cell therapy in kidney transplantation (The ONE Study): a harmonised design and analysis of seven non-randomised, single-arm, phase 1/2A trials. *Lancet* **395**, 1627–1639 (2020). 32446407 10.1016/S0140-6736(20)30167-7 PMC7613154

[20] Roemhild, A. et al. Regulatory T cells for minimising immune suppression in kidney transplantation: phase I/IIa clinical trial. *BMJ* **371**, m3734 (2020). 33087345 10.1136/bmj.m3734 PMC7576328

[21] Beheshti, S. A., Shamsasenjan, K., Ahmadi, M. & Abbasi, B. C. A. R. Treg: a new approach in the treatment of autoimmune diseases. *Int. Immunopharmacol.* **102**, 108409 (2022). 34863655 10.1016/j.intimp.2021.108409

[22] MacDonald, K. G. et al. Alloantigen-specific regulatory T cells generated with a chimeric antigen receptor. *J. Clin. Investig.* **126**, 1413–1424 (2016). 26999600 10.1172/JCI82771 PMC4811124

[23] Bluestone, J. A. et al. Type 1 diabetes immunotherapy using polyclonal regulatory T cells. *Sci. Transl. Med.* **7**, 315ra189 (2015). 26606968 10.1126/scitranslmed.aad4134 PMC4729454

[24] Dong, S. et al. The effect of low-dose IL-2 and Treg adoptive cell therapy in patients with type 1 diabetes. *JCI Insight*. **6**, e147474 (2021). 10.1172/jci.insight.147474 PMC8492314 34324441

[25] MacMillan, M. L. et al. First-in-human phase 1 trial of induced regulatory T cells for graft-versus-host disease prophylaxis in HLA-matched siblings. *Blood Adv.* **5**, 1425–1436 (2021). 33666654 10.1182/bloodadvances.2020003219 PMC7948291

[26] Pierini, A. et al. Haploidentical age-adapted myeloablative transplant and regulatory and effector T cells for acute myeloid leukemia. *Blood Adv.* **5**, 1199–1208 (2021). 33646302 10.1182/bloodadvances.2020003739 PMC7948281

[27] Gershon, R. K. & Kondo, K. Cell interactions in the induction of tolerance: the role of thymic lymphocytes. *Immunology* **18**, 723–737 (1970). 4911896 PMC1455602

[28] Gershon, R. K., Mokyr, M. B. & Mitchell, M. S. Activation of suppressor T cells by tumour cells and specific antibody. *Nature* **250**, 594–596 (1974). 4546445 10.1038/250594a0

[29] Gershon, R. K. & Kondo, K. Infectious immunological tolerance. *Immunology* **21**, 903–914 (1971). 4943147 PMC1408252

[30] Sakaguchi, S. et al. Immunologic self-tolerance maintained by activated T cells expressing IL-2 receptor alpha-chains (CD25). Breakdown of a single mechanism of self-tolerance causes various autoimmune diseases. *J. Immunol.* **155**, 1151–1164 (1995). 7636184

[31] Baecher-Allan, C., Brown, J. A., Freeman, G. J. & Hafler, D. A. CD4+CD25high regulatory cells in human peripheral blood. *J. Immunol.* **167**, 1245–1253 (2001). 11466340 10.4049/jimmunol.167.3.1245

[32] Dieckmann, D. et al. Ex vivo isolation and characterization of CD4(+)CD25(+) T cells with regulatory properties from human blood. *J. Exp. Med.* **193**, 1303–1310 (2001). 11390437 10.1084/jem.193.11.1303 PMC2193384

[33] Jonuleit, H. et al. Identification and functional characterization of human CD4(+)CD25(+) T cells with regulatory properties isolated from peripheral blood. *J. Exp. Med.* **193**, 1285–1294 (2001). 11390435 10.1084/jem.193.11.1285 PMC2193380

[34] Khattri, R., Cox, T., Yasayko, S. A. & Ramsdell, F. An essential role for Scurfin in CD4+CD25+ T regulatory cells. *Nat. Immunol.* **4**, 337–342 (2003). 28115588

[35] Yagi, H. et al. Crucial role of FOXP3 in the development and function of human CD25+CD4+ regulatory T cells. *Int. Immunol.* **16**, 1643–1656 (2004). 15466453 10.1093/intimm/dxh165

[36] Roncador, G. et al. Analysis of FOXP3 protein expression in human CD4+CD25+ regulatory T cells at the single-cell level. *Eur. J. Immunol.* **35**, 1681–1691 (2005). 15902688 10.1002/eji.200526189

[37] Chen, W. et al. Conversion of peripheral CD4+CD25- naive T cells to CD4+CD25+ regulatory T cells by TGF-beta induction of transcription factor Foxp3. *J. Exp. Med.* **198**, 1875–1886 (2003). 14676299 10.1084/jem.20030152 PMC2194145

[38] Trenado, A. et al. Ex vivo-expanded CD4+CD25+ immunoregulatory T cells prevent graft-versus-host-disease by inhibiting activation/differentiation of pathogenic T cells. *J. Immunol.* **176**, 1266–1273 (2006). 16394018 10.4049/jimmunol.176.2.1266

[39] Tanaka, H. et al. Successful immunotherapy of autoimmune cholangitis by adoptive transfer of forkhead box protein 3(+) regulatory T cells. *Clin. Exp. Immunol.* **178**, 253–261 (2014). 25041369 10.1111/cei.12415 PMC4233375

[40] Sagoo, P. et al. Human regulatory T cells with alloantigen specificity are more potent inhibitors of alloimmune skin graft damage than polyclonal regulatory T cells. *Sci. Transl. Med.* **3**, 83ra42 (2011). 21593402 10.1126/scitranslmed.3002076 PMC3776382

[41] Ahmadzadeh, M. et al. Tumor-infiltrating human CD4(+) regulatory T cells display a distinct TCR repertoire and exhibit tumor and neoantigen reactivity. *Sci Immunol*. **4**, eaao4310 (2019). 10.1126/sciimmunol.aao4310 PMC6685542 30635355

[42] Saito, T. et al. Two FOXP3(+)CD4(+) T cell subpopulations distinctly control the prognosis of colorectal cancers. *Nat. Med.* **22**, 679–684 (2016). 27111280 10.1038/nm.4086

[43] Yeapuri, P. et al. Amyloid-β specific regulatory T cells attenuate Alzheimer’s disease pathobiology in APP/PS1 mice. *Mol. Neurodegener.* **18**, 97 (2023). 38111016 10.1186/s13024-023-00692-7 PMC10729469

[44] Yang, H. et al. Adoptive therapy with amyloid-β specific regulatory T cells alleviates Alzheimer’s disease. *Theranostics* **12**, 7668–7680 (2022). 36451854 10.7150/thno.75965 PMC9706584

[45] Trzonkowski, P. et al. First-in-man clinical results of the treatment of patients with graft versus host disease with human ex vivo expanded CD4+CD25+CD127- T regulatory cells. *Clin. Immunol.* **133**, 22–26 (2009). 19559653 10.1016/j.clim.2009.06.001

[46] Edinger, M. & Hoffmann, P. Regulatory T cells in stem cell transplantation: strategies and first clinical experiences. *Curr. Opin. Immunol.* **23**, 679–684 (2011). 21802270 10.1016/j.coi.2011.06.006

[47] Marek-Trzonkowska, N. et al. Therapy of type 1 diabetes with CD4(+)CD25(high)CD127-regulatory T cells prolongs survival of pancreatic islets—results of one year follow-up. *Clin. Immunol.* **153**, 23–30 (2014). 24704576 10.1016/j.clim.2014.03.016

[48] Yamaguchi, T. & Sakaguchi, S. Regulatory T cells in immune surveillance and treatment of cancer. *Semin Cancer Biol.* **16**, 115–123 (2006). 16376102 10.1016/j.semcancer.2005.11.005

[49] Sawant, D. V. et al. Adaptive plasticity of IL-10(+) and IL-35(+) T(reg) cells cooperatively promotes tumor T cell exhaustion. *Nat. Immunol.* **20**, 724–735 (2019). 30936494 10.1038/s41590-019-0346-9 PMC6531353

[50] Long, X. et al. Targeting JMJD1C to selectively disrupt tumor T(reg) cell fitness enhances antitumor immunity. *Nat. Immunol.* **25**, 525–536 (2024). 38356061 10.1038/s41590-024-01746-8

[51] Nie, P. et al. Targeting p97-Npl4 interaction inhibits tumor T(reg) cell development to enhance tumor immunity. *Nat. Immunol.* **25**, 1623–1636 (2024). 39107403 10.1038/s41590-024-01912-y

[52] Hogrebe, N. J., Ishahak, M. & Millman, J. R. Developments in stem cell-derived islet replacement therapy for treating type 1 diabetes. *Cell Stem Cell* **30**, 530–548 (2023). 37146579 10.1016/j.stem.2023.04.002 PMC10167558

[53] Shevach, E. M. & Thornton, A. M. tTregs, pTregs, and iTregs: similarities and differences. *Immunol. Rev.* **259**, 88–102 (2014). 24712461 10.1111/imr.12160 PMC3982187

[54] Thornton, A. M. et al. Expression of Helios, an Ikaros transcription factor family member, differentiates thymic-derived from peripherally induced Foxp3+ T regulatory cells. *J. Immunol.* **184**, 3433–3441 (2010). 20181882 10.4049/jimmunol.0904028 PMC3725574

[55] Weiss, J. M. et al. Neuropilin 1 is expressed on thymus-derived natural regulatory T cells, but not mucosa-generated induced Foxp3+ T reg cells. *J. Exp. Med.* **209**, 1723–1742 (2012). s1721. 22966001 10.1084/jem.20120914 PMC3457733

[56] Yadav, M. et al. Neuropilin-1 distinguishes natural and inducible regulatory T cells among regulatory T cell subsets in vivo. *J. Exp. Med.* **209**, 1713–1722 (2012). 22966003 10.1084/jem.20120822 PMC3457729

[57] Opstelten, R. et al. GPA33: a marker to identify stable human regulatory T cells. *J. Immunol.* **204**, 3139–3148 (2020). 32366581 10.4049/jimmunol.1901250

[58] Zheng, Y. et al. Role of conserved non-coding DNA elements in the Foxp3 gene in regulatory T-cell fate. *Nature* **463**, 808–812 (2010). 20072126 10.1038/nature08750 PMC2884187

[59] Sawant, D. V. & Vignali, D. A. Once a Treg, always a Treg?. *Immunol. Rev.* **259**, 173–191 (2014). 24712466 10.1111/imr.12173 PMC4008876

[60] Seddiki, N. et al. Persistence of naive CD45RA+ regulatory T cells in adult life. *Blood* **107**, 2830–2838 (2006). 16332974 10.1182/blood-2005-06-2403

[61] Booth, N. J. et al. Different proliferative potential and migratory characteristics of human CD4+ regulatory T cells that express either CD45RA or CD45RO. *J. Immunol.* **184**, 4317–4326 (2010). 20231690 10.4049/jimmunol.0903781

[62] Miyara, M. et al. Functional delineation and differentiation dynamics of human CD4+ T cells expressing the FoxP3 transcription factor. *Immunity* **30**, 899–911 (2009). 19464196 10.1016/j.immuni.2009.03.019

[63] Ito, T. et al. Two functional subsets of FOXP3+ regulatory T cells in human thymus and periphery. *Immunity* **28**, 870–880 (2008). 18513999 10.1016/j.immuni.2008.03.018 PMC2709453

[64] Bergot, A. S. et al. TCR sequences and tissue distribution discriminate the subsets of naïve and activated/memory Treg cells in mice. *Eur. J. Immunol.* **45**, 1524–1534 (2015). 25726757 10.1002/eji.201445269

[65] Huehn, J. et al. Developmental stage, phenotype, and migration distinguish naive- and effector/memory-like CD4+ regulatory T cells. *J. Exp. Med.* **199**, 303–313 (2004). 14757740 10.1084/jem.20031562 PMC2211798

[66] Hu, Z. Q. & Zhao, W. H. The IL-33/ST2 axis is specifically required for development of adipose tissue-resident regulatory T cells. *Cell. Mol. Immunol.* **12**, 521–524 (2015). 26277897 10.1038/cmi.2015.49 PMC4579657

[67] Han, J. M. et al. IL-33 Reverses an obesity-induced deficit in visceral adipose tissue ST2+ T regulatory cells and ameliorates adipose tissue inflammation and insulin resistance. *J. Immunol.* **194**, 4777–4783 (2015). 25870243 10.4049/jimmunol.1500020

[68] Cosovanu, C. & Neumann, C. The many functions of Foxp3(+) regulatory T cells in the intestine. *Front. Immunol.* **11**, 600973 (2020). 33193456 10.3389/fimmu.2020.600973 PMC7606913

[69] Yang, B. H. et al. Foxp3(+) T cells expressing RORγt represent a stable regulatory T-cell effector lineage with enhanced suppressive capacity during intestinal inflammation. *Mucosal Immunol.* **9**, 444–457 (2016). 26307665 10.1038/mi.2015.74

[70] Zhang, A. et al. Regulatory T cells in immune checkpoint blockade antitumor therapy. *Mol. Cancer* **23**, 251 (2024). 39516941 10.1186/s12943-024-02156-y PMC11545879

[71] Kim, J. H., Kim, B. S. & Lee, S. K. Regulatory T cells in tumor microenvironment and approach for anticancer immunotherapy. *Immune Netw.* **20**, e4 (2020). 32158592 10.4110/in.2020.20.e4 PMC7049587

[72] Seneschal, J. et al. Human epidermal Langerhans cells maintain immune homeostasis in skin by activating skin resident regulatory T cells. *Immunity* **36**, 873–884 (2012). 22560445 10.1016/j.immuni.2012.03.018 PMC3716276

[73] Li, J., Xiao, C., Li, C. & He, J. Tissue-resident immune cells: from defining characteristics to roles in diseases. *Signal Transduct. Target. Ther.* **10**, 12 (2025). 39820040 10.1038/s41392-024-02050-5 PMC11755756

[74] Yshii, L. et al. Astrocyte-targeted gene delivery of interleukin 2 specifically increases brain-resident regulatory T cell numbers and protects against pathological neuroinflammation. *Nat. Immunol.* **23**, 878–891 (2022). 35618831 10.1038/s41590-022-01208-z PMC9174055

[75] Zemmour, D. et al. Single-cell gene expression reveals a landscape of regulatory T cell phenotypes shaped by the TCR. *Nat. Immunol.* **19**, 291–301 (2018). 29434354 10.1038/s41590-018-0051-0 PMC6069633

[76] Miragaia, R. J. et al. Single-cell transcriptomics of regulatory T cells reveals trajectories of tissue adaptation. *Immunity* **50**, 493–504 e497 (2019). 30737144 10.1016/j.immuni.2019.01.001 PMC6382439

[77] Luo, Y. et al. Single-cell transcriptomic analysis reveals disparate effector differentiation pathways in human T(reg) compartment. *Nat. Commun.* **12**, 3913 (2021). 34162888 10.1038/s41467-021-24213-6 PMC8222404

[78] Azizi, E. et al. Single-cell map of diverse immune phenotypes in the breast tumor microenvironment. *Cell* **174**, 1293–1308.e1236 (2018). 29961579 10.1016/j.cell.2018.05.060 PMC6348010

[79] Trzonkowski, P. et al. CD4+CD25+ T regulatory cells inhibit cytotoxic activity of T CD8+ and NK lymphocytes in the direct cell-to-cell interaction. *Clin. Immunol.* **112**, 258–267 (2004). 15308119 10.1016/j.clim.2004.04.003

[80] Li, Z., Li, D., Tsun, A. & Li, B. FOXP3+ regulatory T cells and their functional regulation. *Cell. Mol. Immunol.* **12**, 558–565 (2015). 25683611 10.1038/cmi.2015.10 PMC4579651

[81] Hahm, K. et al. Helios, a T cell-restricted Ikaros family member that quantitatively associates with Ikaros at centromeric heterochromatin. *Genes Dev.* **12**, 782–796 (1998). 9512513 10.1101/gad.12.6.782 PMC316626

[82] Thornton, A. M. et al. Helios(+) and Helios(-) Treg subpopulations are phenotypically and functionally distinct and express dissimilar TCR repertoires. *Eur. J. Immunol.* **49**, 398–412 (2019). 30620397 10.1002/eji.201847935 PMC6402968

[83] Solomon, I. et al. CD25-T(reg)-depleting antibodies preserving IL-2 signaling on effector T cells enhance effector activation and antitumor immunity. *Nat. Cancer* **1**, 1153–1166 (2020). 33644766 10.1038/s43018-020-00133-0 PMC7116816

[84] Deaglio, S. et al. Adenosine generation catalyzed by CD39 and CD73 expressed on regulatory T cells mediates immune suppression. *J. Exp. Med.* **204**, 1257–1265 (2007). 17502665 10.1084/jem.20062512 PMC2118603

[85] Kobie, J. J. et al. T regulatory and primed uncommitted CD4 T cells express CD73, which suppresses effector CD4 T cells by converting 5’-adenosine monophosphate to adenosine. *J. Immunol.* **177**, 6780–6786 (2006). 17082591 10.4049/jimmunol.177.10.6780

[86] Bono, M. R. et al. CD73 and CD39 ectonucleotidases in T cell differentiation: beyond immunosuppression. *FEBS Lett.* **589**, 3454–3460 (2015). 26226423 10.1016/j.febslet.2015.07.027

[87] Bradley, L. M., Watson, S. R. & Swain, S. L. Entry of naive CD4 T cells into peripheral lymph nodes requires L-selectin. *J. Exp. Med.* **180**, 2401–2406 (1994). 7525854 10.1084/jem.180.6.2401 PMC2191768

[88] Gallatin, W. M., Weissman, I. L. & Butcher, E. C. A cell-surface molecule involved in organ-specific homing of lymphocytes. *Nature* **304**, 30–34 (1983). 6866086 10.1038/304030a0

[89] Venturi, G. M., Conway, R. M., Steeber, D. A. & Tedder, T. F. CD25+CD4+ regulatory T cell migration requires L-selectin expression: L-selectin transcriptional regulation balances constitutive receptor turnover. *J. Immunol.* **178**, 291–300 (2007). 17182566 10.4049/jimmunol.178.1.291

[90] Suffia, I., Reckling, S. K., Salay, G. & Belkaid, Y. A role for CD103 in the retention of CD4+CD25+ Treg and control of Leishmania major infection. *J. Immunol.* **174**, 5444–5455 (2005). 15845457 10.4049/jimmunol.174.9.5444

[91] Redmond, W. L., Ruby, C. E. & Weinberg, A. D. The role of OX40-mediated co-stimulation in T-cell activation and survival. *Crit. Rev. Immunol.* **29**, 187–201 (2009). 19538134 10.1615/critrevimmunol.v29.i3.10 PMC3180959

[92] Kitamura, N. et al. OX40 costimulation can abrogate Foxp3+ regulatory T cell-mediated suppression of antitumor immunity. *Int. J. Cancer* **125**, 630–638 (2009). 19455675 10.1002/ijc.24435 PMC3018329

[93] Yamaguchi, T. et al. Control of immune responses by antigen-specific regulatory T cells expressing the folate receptor. *Immunity* **27**, 145–159 (2007). 17613255 10.1016/j.immuni.2007.04.017

[94] Tian, Y. et al. A novel splice variant of folate receptor 4 predominantly expressed in regulatory T cells. *BMC Immunol.* **13**, 30 (2012). 22694797 10.1186/1471-2172-13-30 PMC3724506

[95] Klein, S., Kretz, C. C., Krammer, P. H. & Kuhn, A. CD127(low/-) and FoxP3(+) expression levels characterize different regulatory T-cell populations in human peripheral blood. *J. Investig. Dermatol.* **130**, 492–499 (2010). 19940860 10.1038/jid.2009.313

[96] Zhou, W. et al. Expression of CD4+CD25+CD127(Low) regulatory T cells and cytokines in peripheral blood of patients with primary liver carcinoma. *Int J. Med. Sci.* **17**, 712–719 (2020). 32218692 10.7150/ijms.44088 PMC7085268

[97] Kolar, P. et al. CTLA-4 (CD152) controls homeostasis and suppressive capacity of regulatory T cells in mice. *Arthritis Rheum.* **60**, 123–132 (2009). 19116935 10.1002/art.24181

[98] Rotte, A. Combination of CTLA-4 and PD-1 blockers for treatment of cancer. *J. Exp. Clin. Cancer Res.* **38**, 255 (2019). 31196207 10.1186/s13046-019-1259-z PMC6567914

[99] Willsmore, Z. N. et al. Combined anti-PD-1 and anti-CTLA-4 checkpoint blockade: treatment of melanoma and immune mechanisms of action. *Eur. J. Immunol.* **51**, 544–556 (2021). 33450785 10.1002/eji.202048747

[100] Amoozgar, Z. et al. Targeting Treg cells with GITR activation alleviates resistance to immunotherapy in murine glioblastomas. *Nat. Commun.* **12**, 2582 (2021). 33976133 10.1038/s41467-021-22885-8 PMC8113440

[101] Joller, N., Anderson, A. C. & Kuchroo, V. K. LAG-3, TIM-3, and TIGIT: distinct functions in immune regulation. *Immunity* **57**, 206–222 (2024). 38354701 10.1016/j.immuni.2024.01.010 PMC10919259

[102] Cai, L. et al. Correction: targeting LAG-3, TIM-3, and TIGIT for cancer immunotherapy. *J. Hematol. Oncol.* **16**, 105 (2023). 37773132 10.1186/s13045-023-01503-8 PMC10543833

[103] Overacre-Delgoffe, A. E. & Vignali, D. A. A. Treg fragility: a prerequisite for effective antitumor immunity?. *Cancer Immunol. Res.* **6**, 882–887 (2018). 30068755 10.1158/2326-6066.CIR-18-0066 PMC6080214

[104] Hori, S. Lineage stability and phenotypic plasticity of Foxp3(+) regulatory T cells. *Immunol. Rev.* **259**, 159–172 (2014). 24712465 10.1111/imr.12175

[105] Sakaguchi, S. et al. The plasticity and stability of regulatory T cells. *Nat. Rev. Immunol.* **13**, 461–467 (2013). 23681097 10.1038/nri3464

[106] Shi, H. & Chi, H. Metabolic control of treg cell stability, plasticity, and tissue-specific heterogeneity. *Front. Immunol.* **10**, 2716 (2019). 31921097 10.3389/fimmu.2019.02716 PMC6917616

[107] Kang, J. H. & Zappasodi, R. Modulating Treg stability to improve cancer immunotherapy. *Trends Cancer* **9**, 911–927 (2023). 37598003 10.1016/j.trecan.2023.07.015 PMC13390109

[108] Watson, M. J. et al. Metabolic support of tumour-infiltrating regulatory T cells by lactic acid. *Nature* **591**, 645–651 (2021). 33589820 10.1038/s41586-020-03045-2 PMC7990682

[109] Gu, J. et al. Tumor metabolite lactate promotes tumorigenesis by modulating MOESIN lactylation and enhancing TGF-beta signaling in regulatory T cells. *Cell Rep.* **39**, 110986 (2022). 35732125 10.1016/j.celrep.2022.110986

[110] Kanno, Y. et al. Transcriptional and epigenetic control of T helper cell specification: molecular mechanisms underlying commitment and plasticity. *Annu. Rev. Immunol.* **30**, 707–731 (2012). 22224760 10.1146/annurev-immunol-020711-075058 PMC3314163

[111] Chen, S. Q. et al. Epigenetically modifying the locus for generation of stable antigen-specific Tregs as cellular therapeutics. *Am. J. Transpl.* **20**, 2366–2379 (2020). 10.1111/ajt.15845 PMC7483360 32167228

[112] von Knethen, A. et al. Histone deacetylation inhibitors as modulators of regulatory T cells. *Int. J. Mol. Sci*. **21**, 2356 (2020). 10.3390/ijms21072356 PMC7177531 32235291

[113] Li, X. et al. Function of a Foxp3 cis-element in protecting regulatory T cell identity. *Cell* **158**, 734–748 (2014). 25126782 10.1016/j.cell.2014.07.030 PMC4151505

[114] Trujillo-Ochoa, J. L., Kazemian, M. & Afzali, B. The role of transcription factors in shaping regulatory T cell identity. *Nat. Rev. Immunol.* **23**, 842–856 (2023). 37336954 10.1038/s41577-023-00893-7 PMC10893967

[115] Ohkura, N. & Sakaguchi, S. Transcriptional and epigenetic basis of Treg cell development and function: its genetic anomalies or variations in autoimmune diseases. *Cell Res.* **30**, 465–474 (2020). 32367041 10.1038/s41422-020-0324-7 PMC7264322

[116] Medof, M. E., Rieder, S. A. & Shevach, E. M. Disabled C3ar1/C5ar1 signaling in Foxp3 T regulatory cells leads to TSDR demethylation and long-term stability. *J. Immunol.* **211**, 1359–1366 (2023). 37756526 10.4049/jimmunol.2300184 PMC10591991

[117] Pokhrel, R. H., Kang, B., Timilshina, M. & Chang, J. H. AMPK amplifies IL2-STAT5 signaling to maintain stability of regulatory T cells in aged mice. *Int. J. Mol. Sci*. **23**, 12384 (2022). 10.3390/ijms232012384 PMC9604214 36293240

[118] Park, Y. J., Yoo, S. A., Kim, M. & Kim, W. U. The role of calcium-calcineurin-NFAT signaling pathway in health and autoimmune diseases. *Front. Immunol.* **11**, 195 (2020). 32210952 10.3389/fimmu.2020.00195 PMC7075805

[119] Rani, A. & Murphy, J. J. STAT5 in cancer and immunity. *J. Interferon Cytokine Res.* **36**, 226–237 (2016). 26716518 10.1089/jir.2015.0054

[120] Zhang, W. et al. Transcriptional and posttranslational regulation of Th17/Treg balance in health and disease. *Eur. J. Immunol.* **51**, 2137–2150 (2021). 34322865 10.1002/eji.202048794

[121] Kim, H. P. & Leonard, W. J. CREB/ATF-dependent T cell receptor-induced FoxP3 gene expression: a role for DNA methylation. *J. Exp. Med.* **204**, 1543–1551 (2007). 17591856 10.1084/jem.20070109 PMC2118651

[122] Tone, Y. et al. Smad3 and NFAT cooperate to induce Foxp3 expression through its enhancer. *Nat. Immunol.* **9**, 194–202 (2008). 18157133 10.1038/ni1549

[123] Dikiy, S. et al. A distal Foxp3 enhancer enables interleukin-2 dependent thymic Treg cell lineage commitment for robust immune tolerance. *Immunity* **54**, 931–946.e911 (2021). 33838102 10.1016/j.immuni.2021.03.020 PMC8317508

[124] Kawakami, R. et al. Distinct Foxp3 enhancer elements coordinate development, maintenance, and function of regulatory T cells. *Immunity* **54**, 947–961.e948 (2021). 33930308 10.1016/j.immuni.2021.04.005

[125] Li, J. et al. Control of Foxp3 induction and maintenance by sequential histone acetylation and DNA demethylation. *Cell Rep.* **37**, 110124 (2021). 34910919 10.1016/j.celrep.2021.110124 PMC8711072

[126] Floess, S. et al. Epigenetic control of the foxp3 locus in regulatory T cells. *PLoS Biol.* **5**, e38 (2007). 17298177 10.1371/journal.pbio.0050038 PMC1783672

[127] Rubtsov, Y. P. et al. Stability of the regulatory T cell lineage in vivo. *Science* **329**, 1667–1671 (2010). 20929851 10.1126/science.1191996 PMC4262151

[128] Rudra, D. et al. Runx-CBFbeta complexes control expression of the transcription factor Foxp3 in regulatory T cells. *Nat. Immunol.* **10**, 1170–1177 (2009). 19767756 10.1038/ni.1795 PMC2764816

[129] Kitoh, A. et al. Indispensable role of the Runx1-Cbfbeta transcription complex for in vivo-suppressive function of FoxP3+ regulatory T cells. *Immunity* **31**, 609–620 (2009). 19800266 10.1016/j.immuni.2009.09.003

[130] Saigusa, R. et al. Fli1-haploinsufficient dermal fibroblasts promote skin-localized transdifferentiation of Th2-like regulatory T cells. *Arthritis Res. Ther.* **20**, 23 (2018). 29415756 10.1186/s13075-018-1521-3 PMC5803841

[131] Ayyoub, M. et al. Human memory FOXP3+ Tregs secrete IL-17 ex vivo and constitutively express the T(H)17 lineage-specific transcription factor RORgamma t. *Proc. Natl. Acad. Sci. USA* **106**, 8635–8640 (2009). 19439651 10.1073/pnas.0900621106 PMC2688993

[132] Zhou, X. et al. Instability of the transcription factor Foxp3 leads to the generation of pathogenic memory T cells in vivo. *Nat. Immunol.* **10**, 1000–1007 (2009). 19633673 10.1038/ni.1774 PMC2729804

[133] Dominguez-Villar, M., Baecher-Allan, C. M. & Hafler, D. A. Identification of T helper type 1-like, Foxp3+ regulatory T cells in human autoimmune disease. *Nat. Med.* **17**, 673–675 (2011). 21540856 10.1038/nm.2389 PMC3675886

[134] Butcher, M. J. et al. Atherosclerosis-driven Treg plasticity results in formation of a dysfunctional subset of plastic IFNgamma+ Th1/Tregs. *Circ. Res.* **119**, 1190–1203 (2016). 27635087 10.1161/CIRCRESAHA.116.309764 PMC5242312

[135] Gao, N., et al. Contribution of Th2-like Treg cells to the pathogenesis of Takayasu’s arteritis. *Clin. Exp. Rheumatol.* **38**(Suppl 124), 48–54 (2020). 31969221

[136] Halim, L. et al. An atlas of human regulatory T helper-like cells reveals features of Th2-like Tregs that support a tumorigenic environment. *Cell Rep.* **20**, 757–770 (2017). 28723576 10.1016/j.celrep.2017.06.079 PMC5529316

[137] Sawant, D. V. et al. Bcl6 controls the Th2 inflammatory activity of regulatory T cells by repressing Gata3 function. *J. Immunol.* **189**, 4759–4769 (2012). 23053511 10.4049/jimmunol.1201794 PMC3490013

[138] Koenen, H. J. et al. Human CD25highFoxp3pos regulatory T cells differentiate into IL-17-producing cells. *Blood* **112**, 2340–2352 (2008). 18617638 10.1182/blood-2008-01-133967

[139] Ivanov, I. I. et al. The orphan nuclear receptor RORgammat directs the differentiation program of proinflammatory IL-17+ T helper cells. *Cell* **126**, 1121–1133 (2006). 16990136 10.1016/j.cell.2006.07.035

[140] Beriou, G. et al. IL-17-producing human peripheral regulatory T cells retain suppressive function. *Blood* **113**, 4240–4249 (2009). 19171879 10.1182/blood-2008-10-183251 PMC2676084

[141] Voo, K. S. et al. Identification of IL-17-producing FOXP3+ regulatory T cells in humans. *Proc. Natl. Acad. Sci. USA* **106**, 4793–4798 (2009). 19273860 10.1073/pnas.0900408106 PMC2653560

[142] Zhou, L. et al. TGF-beta-induced Foxp3 inhibits T(H)17 cell differentiation by antagonizing RORgammat function. *Nature* **453**, 236–240 (2008). 18368049 10.1038/nature06878 PMC2597437

[143] Linterman, M. A. et al. Foxp3+ follicular regulatory T cells control the germinal center response. *Nat. Med.* **17**, 975–982 (2011). 21785433 10.1038/nm.2425 PMC3182542

[144] Chung, Y. et al. Follicular regulatory T cells expressing Foxp3 and Bcl-6 suppress germinal center reactions. *Nat. Med.* **17**, 983–988 (2011). 21785430 10.1038/nm.2426 PMC3151340

[145] Chi, H. Immunometabolism at the intersection of metabolic signaling, cell fate, and systems immunology. *Cell. Mol. Immunol.* **19**, 299–302 (2022). 35190684 10.1038/s41423-022-00840-x PMC8891332

[146] Wang, A., Luan, H. H. & Medzhitov, R. An evolutionary perspective on immunometabolism. *Science* **363**, eaar3932 (2019). 30630899 10.1126/science.aar3932 PMC6892590

[147] Xiao, S. et al. Retinoic acid increases Foxp3+ regulatory T cells and inhibits development of Th17 cells by enhancing TGF-beta-driven Smad3 signaling and inhibiting IL-6 and IL-23 receptor expression. *J. Immunol.* **181**, 2277–2284 (2008). 18684916 10.4049/jimmunol.181.4.2277 PMC2722959

[148] Yue, X. et al. Control of Foxp3 stability through modulation of TET activity. *J. Exp. Med.* **213**, 377–397 (2016). 26903244 10.1084/jem.20151438 PMC4813667

[149] Sasidharan Nair, V., Song, M. H. & Oh, K. I. Vitamin C facilitates demethylation of the Foxp3 enhancer in a tet-dependent manner. *J. Immunol.* **196**, 2119–2131 (2016). 26826239 10.4049/jimmunol.1502352

[150] Joshi, S. et al. 1,25-dihydroxyvitamin D(3) ameliorates Th17 autoimmunity via transcriptional modulation of interleukin-17A. *Mol. Cell Biol.* **31**, 3653–3669 (2011). 21746882 10.1128/MCB.05020-11 PMC3165548

[151] Jeffery, L. E. et al. 1,25-Dihydroxyvitamin D3 and IL-2 combine to inhibit T cell production of inflammatory cytokines and promote development of regulatory T cells expressing CTLA-4 and FoxP3. *J. Immunol.* **183**, 5458–5467 (2009). 19843932 10.4049/jimmunol.0803217 PMC2810518

[152] Kang, S. W. et al. 1,25-Dihyroxyvitamin D3 promotes FOXP3 expression via binding to vitamin D response elements in its conserved noncoding sequence region. *J. Immunol.* **188**, 5276–5282 (2012). 22529297 10.4049/jimmunol.1101211 PMC3358577

[153] Michalek, R. D. et al. Cutting edge: distinct glycolytic and lipid oxidative metabolic programs are essential for effector and regulatory CD4+ T cell subsets. *J. Immunol.* **186**, 3299–3303 (2011). 21317389 10.4049/jimmunol.1003613 PMC3198034

[154] Chang, C. H. et al. Posttranscriptional control of T cell effector function by aerobic glycolysis. *Cell* **153**, 1239–1251 (2013). 23746840 10.1016/j.cell.2013.05.016 PMC3804311

[155] Angelin, A. et al. Foxp3 reprograms T cell metabolism to function in low-glucose, high-lactate environments. *Cell Metab.* **25**, 1282–1293.e1287 (2017). 28416194 10.1016/j.cmet.2016.12.018 PMC5462872

[156] Zhang, W. et al. SRC2 controls CD4(+) T cell activation via stimulating c-Myc-mediated upregulation of amino acid transporter Slc7a5. *Proc. Natl. Acad. Sci. USA* **120**, e2221352120 (2023). 37094160 10.1073/pnas.2221352120 PMC10160970

[157] Field, C. S. et al. Mitochondrial integrity regulated by lipid metabolism is a cell-intrinsic checkpoint for Treg suppressive function. *Cell Metab.* **31**, 422–437.e425 (2020). 31883840 10.1016/j.cmet.2019.11.021 PMC7001036

[158] Lin, L. et al. Oleic acid availability impacts thymocyte preprogramming and subsequent peripheral T(reg) cell differentiation. *Nat. Immunol.* **25**, 54–65 (2024). 38062135 10.1038/s41590-023-01672-1 PMC10918613

[159] Miao, Y. et al. The activation of PPARgamma enhances Treg responses through up-regulating CD36/CPT1-mediated fatty acid oxidation and subsequent N-glycan branching of TbetaRII/IL-2Ralpha. *Cell Commun. Signal* **20**, 48 (2022). 35392915 10.1186/s12964-022-00849-9 PMC8991706

[160] Yan, Y. et al. Metabolic profiles of regulatory T cells and their adaptations to the tumor microenvironment: implications for antitumor immunity. *J. Hematol. Oncol.* **15**, 104 (2022). 35948909 10.1186/s13045-022-01322-3 PMC9364625

[161] Wang, H. et al. CD36-mediated metabolic adaptation supports regulatory T cell survival and function in tumors. *Nat. Immunol.* **21**, 298–308 (2020). 32066953 10.1038/s41590-019-0589-5 PMC7043937

[162] Saravia, J. et al. Homeostasis and transitional activation of regulatory T cells require c-Myc. *Sci. Adv.* **6**, eaaw6443 (2020). 31911938 10.1126/sciadv.aaw6443 PMC6938709

[163] Danileviciute, E. et al. PARK7/DJ-1 promotes pyruvate dehydrogenase activity and maintains T(reg) homeostasis during ageing. *Nat. Metab.* **4**, 589–607 (2022). 35618940 10.1038/s42255-022-00576-y

[164] Sugiura, A. et al. MTHFD2 is a metabolic checkpoint controlling effector and regulatory T cell fate and function. *Immunity* **55**, 65–81.e69 (2022). 34767747 10.1016/j.immuni.2021.10.011 PMC8755618

[165] Covarrubias, A. J., Perrone, R., Grozio, A. & Verdin, E. NAD(+) metabolism and its roles in cellular processes during ageing. *Nat. Rev. Mol. Cell Biol.* **22**, 119–141 (2021). 33353981 10.1038/s41580-020-00313-x PMC7963035

[166] Kim, M. J. et al. Deletion of PD-1 destabilizes the lineage identity and metabolic fitness of tumor-infiltrating regulatory T cells. *Nat. Immunol.* **24**, 148–161 (2023). 36577929 10.1038/s41590-022-01373-1

[167] Kamada, T. et al. PD-1(+) regulatory T cells amplified by PD-1 blockade promote hyperprogression of cancer. *Proc. Natl. Acad. Sci. USA* **116**, 9999–10008 (2019). 31028147 10.1073/pnas.1822001116 PMC6525547

[168] Liu, Q. et al. Non-oxidative pentose phosphate pathway controls regulatory T cell function by integrating metabolism and epigenetics. *Nat. Metab.* **4**, 559–574 (2022). 35606596 10.1038/s42255-022-00575-z

[169] Weinberg, S. E. et al. Mitochondrial complex III is essential for suppressive function of regulatory T cells. *Nature* **565**, 495–499 (2019). 30626970 10.1038/s41586-018-0846-z PMC6345596

[170] Huang, C. et al. SENP3 is responsible for HIF-1 transactivation under mild oxidative stress via p300 de-SUMOylation. *EMBO j.* **28**, 2748–2762 (2009). 19680224 10.1038/emboj.2009.210 PMC2750016

[171] Yan, S. et al. Redox regulation of the stability of the SUMO protease SENP3 via interactions with CHIP and Hsp90. *EMBO J.* **29**, 3773–3786 (2010). 20924358 10.1038/emboj.2010.245 PMC2989103

[172] Yu, X. et al. SENP3 maintains the stability and function of regulatory T cells via BACH2 deSUMOylation. *Nat. Commun.* **9**, 3157 (2018). 30089837 10.1038/s41467-018-05676-6 PMC6082899

[173] Kishore, M. et al. Regulatory T cell migration is dependent on glucokinase-mediated glycolysis. *Immunity* **47**, 875–889.e810 (2017). 29166588 10.1016/j.immuni.2017.10.017 PMC5714502

[174] Vallion, R. et al. Regulatory T cell stability and migration are dependent on mTOR. *J. Immunol.* **205**, 1799–1809 (2020). 32839235 10.4049/jimmunol.1901480

[175] Ben-Shoshan, J. et al. Hypoxia controls CD4+CD25+ regulatory T-cell homeostasis via hypoxia-inducible factor-1alpha. *Eur. J. Immunol.* **38**, 2412–2418 (2008). 18792019 10.1002/eji.200838318

[176] Clambey, E. T. et al. Hypoxia-inducible factor-1 alpha-dependent induction of FoxP3 drives regulatory T-cell abundance and function during inflammatory hypoxia of the mucosa. *Proc. Natl. Acad. Sci. USA* **109**, E2784–E2793 (2012). 22988108 10.1073/pnas.1202366109 PMC3478644

[177] Miska, J. et al. HIF-1α is a metabolic switch between glycolytic-driven migration and oxidative phosphorylation-driven immunosuppression of Tregs in glioblastoma. *Cell Rep.* **27**, 226–237.e224 (2019). 30943404 10.1016/j.celrep.2019.03.029 PMC6461402

[178] Lee, J. H., Elly, C., Park, Y. & Liu, Y. C. E3 Ubiquitin ligase VHL regulates hypoxia-inducible factor-1α to Maintain Regulatory T cell stability and suppressive capacity. *Immunity* **42**, 1062–1074 (2015). 26084024 10.1016/j.immuni.2015.05.016 PMC4498255

[179] Li, L. et al. TLR8-mediated metabolic control of human Treg function: a mechanistic target for cancer immunotherapy. *Cell Metab.* **29**, 103–123.e105 (2019). 30344014 10.1016/j.cmet.2018.09.020 PMC7050437

[180] van der Windt, G. J. & Pearce, E. L. Metabolic switching and fuel choice during T-cell differentiation and memory development. *Immunol. Rev.* **249**, 27–42 (2012). 22889213 10.1111/j.1600-065X.2012.01150.x PMC3645891

[181] Shaw, R. J. et al. The tumor suppressor LKB1 kinase directly activates AMP-activated kinase and regulates apoptosis in response to energy stress. *Proc. Natl. Acad. Sci. USA* **101**, 3329–3335 (2004). 14985505 10.1073/pnas.0308061100 PMC373461

[182] Woods, A. et al. LKB1 is the upstream kinase in the AMP-activated protein kinase cascade. *Curr. Biol.* **13**, 2004–2008 (2003). 14614828 10.1016/j.cub.2003.10.031

[183] Wu, D. et al. Lkb1 maintains T(reg) cell lineage identity. *Nat. Commun.* **8**, 15876 (2017). 28621313 10.1038/ncomms15876 PMC5481770

[184] Timilshina, M. et al. Activation of mevalonate pathway via LKB1 is essential for stability of T(reg) cells. *Cell Rep.* **27**, 2948–2961.e2947 (2019). 31167140 10.1016/j.celrep.2019.05.020

[185] Yang, K. et al. Homeostatic control of metabolic and functional fitness of T(reg) cells by LKB1 signalling. *Nature* **548**, 602–606 (2017). 28847007 10.1038/nature23665 PMC5804356

[186] He, N. et al. Metabolic control of regulatory T cell (Treg) survival and function by Lkb1. *Proc. Natl. Acad. Sci. USA* **114**, 12542–12547 (2017). 29109251 10.1073/pnas.1715363114 PMC5703326

[187] Shi, H. et al. Amino acids license kinase mTORC1 activity and Treg cell function via small G proteins Rag and Rheb. *Immunity* **51**, 1012–1027.e1017 (2019). 31668641 10.1016/j.immuni.2019.10.001 PMC6948188

[188] Do, M. H. et al. Nutrient mTORC1 signaling underpins regulatory T cell control of immune tolerance. *J. Exp. Med.* **217**, e20190848 (2020). 31649036 10.1084/jem.20190848 PMC7037250

[189] Schiering, C. et al. The alarmin IL-33 promotes regulatory T-cell function in the intestine. *Nature* **513**, 564–568 (2014). 25043027 10.1038/nature13577 PMC4339042

[190] Kolodin, D. et al. Antigen- and cytokine-driven accumulation of regulatory T cells in visceral adipose tissue of lean mice. *Cell Metab.* **21**, 543–557 (2015). 25863247 10.1016/j.cmet.2015.03.005 PMC4747251

[191] Li, C. et al. TCR transgenic mice reveal stepwise, multi-site acquisition of the distinctive fat-Treg phenotype. *Cell* **174**, 285–299.e212 (2018). 29887374 10.1016/j.cell.2018.05.004 PMC6046274

[192] Delacher, M. et al. Precursors for nonlymphoid-tissue Treg cells reside in secondary lymphoid organs and are programmed by the transcription factor BATF. *Immunity* **52**, 295–312.e211 (2020). 31924477 10.1016/j.immuni.2019.12.002 PMC7026712

[193] Vasanthakumar, A. & Kallies, A. The regulatory T cell: jack-of-all-trades. *Trends Immunol.* **36**, 756–758 (2015). 26511762 10.1016/j.it.2015.10.002

[194] Quandt, J. et al. Wnt-beta-catenin activation epigenetically reprograms T(reg) cells in inflammatory bowel disease and dysplastic progression. *Nat. Immunol.* **22**, 471–484 (2021). 33664518 10.1038/s41590-021-00889-2 PMC8262575

[195] Gazzerro, E. et al. Enhancement of muscle T regulatory cells and improvement of muscular dystrophic process in MDX mice by blockade of extracellular ATP/P2X axis. *Am. J. Pathol.* **185**, 3349–3360 (2015). 26465071 10.1016/j.ajpath.2015.08.010

[196] Schenk, U. et al. ATP inhibits the generation and function of regulatory T cells through the activation of purinergic P2X receptors. *Sci. Signal.* **4**, ra12 (2011). 21364186 10.1126/scisignal.2001270

[197] Villalta, S. A. et al. Regulatory T cells suppress muscle inflammation and injury in muscular dystrophy. *Sci. Transl. Med.* **6**, 258ra142 (2014). 25320234 10.1126/scitranslmed.3009925 PMC4889432

[198] Garg, G. et al. Blimp1 prevents methylation of Foxp3 and loss of regulatory T cell identity at sites of inflammation. *Cell Rep.* **26**, 1854–1868.e1855 (2019). 30759395 10.1016/j.celrep.2019.01.070 PMC6389594

[199] Wagner, E. J. & Carpenter, P. B. Understanding the language of Lys36 methylation at histone H3. *Nat. Rev. Mol. Cell Biol.* **13**, 115–126 (2012). 22266761 10.1038/nrm3274 PMC3969746

[200] Ji, Z. et al. The histone methyltransferase Setd2 is indispensable for V(D)J recombination. *Nat. Commun.* **10**, 3353 (2019). 31350389 10.1038/s41467-019-11282-x PMC6659703

[201] Xu, Q. et al. SETD2 regulates the maternal epigenome, genomic imprinting and embryonic development. *Nat. Genet* **51**, 844–856 (2019). 31040401 10.1038/s41588-019-0398-7

[202] Hu, M. et al. Histone H3 lysine 36 methyltransferase Hypb/Setd2 is required for embryonic vascular remodeling. *Proc. Natl. Acad. Sci. USA* **107**, 2956–2961 (2010). 20133625 10.1073/pnas.0915033107 PMC2840328

[203] Chen, K. et al. Methyltransferase SETD2-Mediated Methylation of STAT1 is critical for interferon antiviral activity. *Cell* **170**, 492–506.e414 (2017). 28753426 10.1016/j.cell.2017.06.042

[204] Ding, Z. et al. Setd2 supports GATA3+ST2+ thymic-derived Treg cells and suppresses intestinal inflammation. *Nat. Commun.* **13**, 7468 (2022). 36463230 10.1038/s41467-022-35250-0 PMC9719510

[205] Zhang, W. et al. Steroid nuclear receptor coactivator 2 controls immune tolerance by promoting induced T(reg) differentiation via up-regulating Nr4a2. *Sci. Adv.* **8**, eabn7662 (2022). 35704583 10.1126/sciadv.abn7662 PMC9200286

[206] Shi, Y. et al. Cancer-associated SF3B1-K700E mutation controls immune responses by regulating T(reg) function via aberrant Anapc13 splicing. *Sci. Adv.* **10**, eado4274 (2024). 39303038 10.1126/sciadv.ado4274 PMC11414738

[207] Wang, A. et al. ZFP91 is required for the maintenance of regulatory T cell homeostasis and function. *J Exp. Med*. **218**, e20201217 (2021). 10.1084/jem.20201217 PMC7769166 33355624

[208] Wu, D. et al. The Ube2m-Rbx1 neddylation-Cullin-RING-Ligase proteins are essential for the maintenance of Regulatory T cell fitness. *Nat. Commun.* **13**, 3021 (2022). 35641500 10.1038/s41467-022-30707-8 PMC9156764

[209] Yang, X. et al. RNF213 promotes Treg cell differentiation by facilitating K63-linked ubiquitination and nuclear translocation of FOXO1. *Nat. Commun.* **15**, 5961 (2024). 39013878 10.1038/s41467-024-50392-z PMC11252262

[210] Montauti, E. et al. A deubiquitination module essential for T(reg) fitness in the tumor microenvironment. *Sci. Adv.* **8**, eabo4116 (2022). 36427305 10.1126/sciadv.abo4116 PMC9699683

[211] Grant, F. M. et al. BACH2 drives quiescence and maintenance of resting Treg cells to promote homeostasis and cancer immunosuppression. *J. Exp. Med.* **217**, e20190711 (2020). 32515782 10.1084/jem.20190711 PMC7478731

[212] Rosetti, F., Madera-Salcedo, I. K., Rodriguez-Rodriguez, N. & Crispin, J. C. Regulation of activated T cell survival in rheumatic autoimmune diseases. *Nat. Rev. Rheumatol.* **18**, 232–244 (2022). 35075294 10.1038/s41584-021-00741-9

[213] Li, X. et al. Dysfunctions, molecular mechanisms, and therapeutic strategies of regulatory T cells in rheumatoid arthritis. *Front. Pharm.* **12**, 716081 (2021). 10.3389/fphar.2021.716081 PMC8428974 34512345

[214] Beavis, P. A. et al. Resistance to regulatory T cell-mediated suppression in rheumatoid arthritis can be bypassed by ectopic foxp3 expression in pathogenic synovial T cells. *Proc. Natl. Acad. Sci. USA* **108**, 16717–16722 (2011). 21926327 10.1073/pnas.1112722108 PMC3189031

[215] Yan, S., Kotschenreuther, K., Deng, S. & Kofler, D. M. Regulatory T cells in rheumatoid arthritis: functions, development, regulation, and therapeutic potential. *Cell Mol. Life Sci.* **79**, 533 (2022). 36173485 10.1007/s00018-022-04563-0 PMC9522664

[216] Sun, J. et al. Efficient therapeutic function and mechanisms of human polyclonal CD8(+)CD103(+)Foxp3(+) regulatory T cells on collagen-induced arthritis in mice. *J. Immunol. Res.* **2019**, 8575407 (2019). 30915372 10.1155/2019/8575407 PMC6399536

[217] Li, J. et al. KIR(+)CD8(+) T cells suppress pathogenic T cells and are active in autoimmune diseases and COVID-19. *Science* **376**, eabi9591 (2022). 35258337 10.1126/science.abi9591 PMC8995031

[218] Koh, C. H. et al. CD8 T-cell subsets: heterogeneity, functions, and therapeutic potential. *Exp. Mol. Med.* **55**, 2287–2299 (2023). 37907738 10.1038/s12276-023-01105-x PMC10689838

[219] Rezaei Kahmini, F., Shahgaldi, S., Azimi, M. & Mansourabadi, A. H. Emerging therapeutic potential of regulatory T (Treg) cells for rheumatoid arthritis: new insights and challenges. *Int. Immunopharmacol.* **108**, 108858 (2022). 35597122 10.1016/j.intimp.2022.108858

[220] Wong, H. S. et al. A local regulatory T cell feedback circuit maintains immune homeostasis by pruning self-activated T cells. *Cell* **184**, 3981–3997 e3922 (2021). 34157301 10.1016/j.cell.2021.05.028 PMC8390950

[221] Hosseini, A. et al. CTLA-4: from mechanism to autoimmune therapy. *Int Immunopharmacol.* **80**, 106221 (2020). 32007707 10.1016/j.intimp.2020.106221

[222] Fang, Y. et al. Epigenetic regulatory axis MIR22-TET3-MTRNR2L2 represses fibroblast-like synoviocyte-mediated inflammation in rheumatoid arthritis. *Arthritis Rheumatol.* **76**, 845–856 (2024). 38221658 10.1002/art.42795

[223] Rossetti, M. et al. Ex vivo-expanded but not in vitro-induced human regulatory T cells are candidates for cell therapy in autoimmune diseases thanks to stable demethylation of the FOXP3 regulatory T cell-specific demethylated region. *J. Immunol.* **194**, 113–124 (2015). 25452562 10.4049/jimmunol.1401145 PMC4383769

[224] Yoshida, H. et al. Effects of interleukin-6 signal inhibition on Treg subpopulations and association of Tregs with clinical outcomes in rheumatoid arthritis. *Rheumatology* **63**, 2515–2524 (2024). 38530780 10.1093/rheumatology/keae196 PMC11371379

[225] Eugster, A. et al. Physiological and pathogenic T cell autoreactivity converge in type 1 diabetes. *Nat. Commun.* **15**, 9204 (2024). 39472557 10.1038/s41467-024-53255-9 PMC11522472

[226] Bettini, M. & Bettini, M. L. Function, failure, and the future potential of Tregs in type 1 diabetes. *Diabetes* **70**, 1211–1219 (2021). 34016597 10.2337/dbi18-0058 PMC8275894

[227] Todd, J. A. et al. Robust associations of four new chromosome regions from genome-wide analyses of type 1 diabetes. *Nat. Genet* **39**, 857–864 (2007). 17554260 10.1038/ng2068 PMC2492393

[228] Kukreja, A. et al. Multiple immuno-regulatory defects in type-1 diabetes. *J. Clin. Invest* **109**, 131–140 (2002). 11781358 10.1172/JCI13605 PMC150819

[229] Santoso, C. et al. Autoimmune diseases and the risk and prognosis of latent autoimmune diabetes in adults. *Diabetologia* **68**, 331–341 (2024). 10.1007/s00125-024-06303-4 PMC11732938 39467873

[230] Sutton, C. E. et al. Interleukin-1 and IL-23 induce innate IL-17 production from gammadelta T cells, amplifying Th17 responses and autoimmunity. *Immunity* **31**, 331–341 (2009). 19682929 10.1016/j.immuni.2009.08.001

[231] Attfield, K. E. et al. The immunology of multiple sclerosis. *Nat. Rev. Immunol.* **22**, 734–750 (2022). 35508809 10.1038/s41577-022-00718-z

[232] Yang, H. et al. Cost-effectiveness analysis of ocrelizumab versus subcutaneous interferon beta-1a for the treatment of relapsing multiple sclerosis. *J. Med. Econ.* **20**, 1056–1065 (2017). 28703659 10.1080/13696998.2017.1355310

[233] Hauser, S. L. et al. Ocrelizumab versus Interferon Beta-1a in relapsing multiple sclerosis. *N. Engl. J. Med.* **376**, 221–234 (2017). 28002679 10.1056/NEJMoa1601277

[234] Fox, R. J. et al. Placebo-controlled phase 3 study of oral BG-12 or glatiramer in multiple sclerosis. *N. Engl. J. Med.* **367**, 1087–1097 (2012). 22992072 10.1056/NEJMoa1206328

[235] Li, W., Deng, C., Yang, H. & Wang, G. The regulatory T cell in active systemic lupus erythematosus patients: a systemic review and meta-analysis. *Front. Immunol.* **10**, 159 (2019). 30833946 10.3389/fimmu.2019.00159 PMC6387904

[236] Muller, F. et al. CD19 CAR T-cell therapy in autoimmune disease—a case series with follow-up. *N. Engl. J. Med.* **390**, 687–700 (2024). 38381673 10.1056/NEJMoa2308917

[237] Mougiakakos, D. et al. CD19-targeted CAR T cells in refractory systemic lupus erythematosus. *N. Engl. J. Med.* **385**, 567–569 (2021). 34347960 10.1056/NEJMc2107725

[238] Raeber, M. E. et al. Interleukin-2 immunotherapy reveals human regulatory T cell subsets with distinct functional and tissue-homing characteristics. *Immunity* **57**, 2232–2250 e2210 (2024). 39137779 10.1016/j.immuni.2024.07.016

[239] Qin, D. et al. Targeting tumor-infiltrating tregs for improved antitumor responses. *Front. Immunol.* **15**, 1325946 (2024). 38500876 10.3389/fimmu.2024.1325946 PMC10944859

[240] Li, C. et al. Regulatory T cells in tumor microenvironment: new mechanisms, potential therapeutic strategies and future prospects. *Mol. Cancer* **19**, 116 (2020). 32680511 10.1186/s12943-020-01234-1 PMC7367382

[241] Frey, D. M. et al. High frequency of tumor-infiltrating FOXP3(+) regulatory T cells predicts improved survival in mismatch repair-proficient colorectal cancer patients. *Int. J. Cancer* **126**, 2635–2643 (2010). 19856313 10.1002/ijc.24989

[242] deLeeuw, R. J., Kost, S. E., Kakal, J. A. & Nelson, B. H. The prognostic value of FoxP3+ tumor-infiltrating lymphocytes in cancer: a critical review of the literature. *Clin. Cancer Res.* **18**, 3022–3029 (2012). 22510350 10.1158/1078-0432.CCR-11-3216

[243] Bergsland, C. H. et al. Spatial analysis and CD25-expression identify regulatory T cells as predictors of a poor prognosis in colorectal cancer. *Mod. Pathol.* **35**, 1236–1246 (2022). 35484226 10.1038/s41379-022-01086-8 PMC9424114

[244] Kidani, Y. et al. CCR8-targeted specific depletion of clonally expanded Treg cells in tumor tissues evokes potent tumor immunity with long-lasting memory. *Proc. Natl. Acad. Sci. USA* **119**, e2114282119 (2022). 35140181 10.1073/pnas.2114282119 PMC8851483

[245] Tay, C., Tanaka, A. & Sakaguchi, S. Tumor-infiltrating regulatory T cells as targets of cancer immunotherapy. *Cancer Cell* **41**, 450–465 (2023). 36917950 10.1016/j.ccell.2023.02.014

[246] Gong, L. et al. Nasopharyngeal carcinoma cells promote regulatory T cell development and suppressive activity via CD70-CD27 interaction. *Nat. Commun.* **14**, 1912 (2023). 37024479 10.1038/s41467-023-37614-6 PMC10079957

[247] Wang, Y., Li, J., Nakahata, S. & Iha, H. Complex role of regulatory T cells (Tregs) in the Tumor microenvironment: their molecular mechanisms and bidirectional effects on cancer progression. *Int. J. Mol. Sci*. **25**, 7346 (2024). 10.3390/ijms25137346 PMC11242872 39000453

[248] Iglesias-Escudero, M., Arias-Gonzalez, N. & Martinez-Caceres, E. Regulatory cells and the effect of cancer immunotherapy. *Mol. Cancer* **22**, 26 (2023). 36739406 10.1186/s12943-023-01714-0 PMC9898962

[249] Kim, H. R. et al. Tumor microenvironment dictates regulatory T cell phenotype: upregulated immune checkpoints reinforce suppressive function. *J. Immunother. Cancer* **7**, 339 (2019). 31801611 10.1186/s40425-019-0785-8 PMC6894345

[250] Zhou, Z. et al. Infiltrating treg reprogramming in the tumor immune microenvironment and its optimization for immunotherapy. *Biomark. Res.* **12**, 97 (2024). 39227959 10.1186/s40364-024-00630-9 PMC11373505

[251] Lv, Q. et al. CSF1R inhibition reprograms tumor-associated macrophages to potentiate anti-PD-1 therapy efficacy against colorectal cancer. *Pharm. Res.* **202**, 107126 (2024). 10.1016/j.phrs.2024.107126 38432446

[252] de Visser, K. E. & Joyce, J. A. The evolving tumor microenvironment: from cancer initiation to metastatic outgrowth. *Cancer Cell* **41**, 374–403 (2023). 36917948 10.1016/j.ccell.2023.02.016

[253] Maharaj, K., Uriepero, A., Sahakian, E. & Pinilla-Ibarz, J. Regulatory T cells (Tregs) in lymphoid malignancies and the impact of novel therapies. *Front. Immunol.* **13**, 943354 (2022). 35979372 10.3389/fimmu.2022.943354 PMC9376239

[254] Weiss, L. et al. Regulatory T cells predict the time to initial treatment in early stage chronic lymphocytic leukemia. *Cancer* **117**, 2163–2169 (2011). 21523729 10.1002/cncr.25752

[255] Spasevska, I. et al. Diversity of intratumoral regulatory T cells in B-cell non-Hodgkin lymphoma. *Blood Adv.* **7**, 7216–7230 (2023). 37695745 10.1182/bloodadvances.2023010158 PMC10698546

[256] Jaime-Ramirez, A. C. et al. NK cell-mediated antitumor effects of a folate-conjugated immunoglobulin are enhanced by cytokines. *Cancer Immunol. Res.* **4**, 323–336 (2016). 26865456 10.1158/2326-6066.CIR-15-0168 PMC4818694

[257] Sur, S. Y. et al. Anti-tumor effect of activated canine B cells with interleukin-21 and anti-B cell receptor. *Anticancer Res.* **43**, 4007–4014 (2023). 37648292 10.21873/anticanres.16588

[258] Rubino, V. et al. IL-21/IL-21R signaling renders acute myeloid leukemia stem cells more susceptible to cytarabine treatment and CAR T cell therapy. *Cell Rep. Med.* **5**, 101826 (2024). 39536753 10.1016/j.xcrm.2024.101826 PMC11604404

[259] Balsas, P. et al. SOX11, CD70, and Treg cells configure the tumor-immune microenvironment of aggressive mantle cell lymphoma. *Blood* **138**, 2202–2215 (2021). 34189576 10.1182/blood.2020010527 PMC8641098

[260] Maj, T. et al. Oxidative stress controls regulatory T cell apoptosis and suppressor activity and PD-L1-blockade resistance in tumor. *Nat. Immunol.* **18**, 1332–1341 (2017). 29083399 10.1038/ni.3868 PMC5770150

[261] Sundstrom, P. et al. Regulatory T cells from colon cancer patients inhibit effector T-cell migration through an adenosine-dependent mechanism. *Cancer Immunol. Res.* **4**, 183–193 (2016). 26787824 10.1158/2326-6066.CIR-15-0050

[262] Elias, S. et al. CXCR4+ Treg cells control serum IgM levels and natural IgM autoantibody production by B-1 cells in the bone marrow. *J. Exp. Med*. **219**, e20220047 (2022). 10.1084/jem.20220047 PMC9178519 35670812

[263] Zhang, S. et al. The alterations in and the role of the Th17/Treg balance in metabolic diseases. *Front Immunol.* **12**, 678355 (2021). 34322117 10.3389/fimmu.2021.678355 PMC8311559

[264] Yuan, N. et al. Expression of CD4+CD25+Foxp3+ regulatory T cells, interleukin 10 and transforming growth factor beta in newly diagnosed type 2 diabetic patients. *Exp. Clin. Endocrinol. Diab* **126**, 96–101 (2018). 10.1055/s-0043-113454 28954308

[265] Knochelmann, H. M. et al. When worlds collide: Th17 and Treg cells in cancer and autoimmunity. *Cell Mol. Immunol.* **15**, 458–469 (2018). 29563615 10.1038/s41423-018-0004-4 PMC6068176

[266] Shaikh, S. R., Beck, M. A., Alwarawrah, Y. & MacIver, N. J. Emerging mechanisms of obesity-associated immune dysfunction. *Nat. Rev. Endocrinol.* **20**, 136–148 (2024). 38129700 10.1038/s41574-023-00932-2 PMC13082746

[267] Ma, Y. & Cao, H. Lnc-ing’ T(reg) cells to the aging liver. *Nat. Aging* **3**, 760–761 (2023). 37291220 10.1038/s43587-023-00439-5

[268] Tang, L. et al. Dysfunction of circulating CD3(+)CD56(+) NKT-like cells in type 2 diabetes mellitus. *Int. J. Med. Sci.* **20**, 652–662 (2023). 37082729 10.7150/ijms.83317 PMC10110473

[269] Sharma, A. & Rudra, D. Emerging functions of regulatory T cells in tissue homeostasis. *Front. Immunol.* **9**, 883 (2018). 29887862 10.3389/fimmu.2018.00883 PMC5989423

[270] Bour-Jordan, H. & Bluestone, J. A. Regulating the regulators: costimulatory signals control the homeostasis and function of regulatory T cells. *Immunol. Rev.* **229**, 41–66 (2009). 19426214 10.1111/j.1600-065X.2009.00775.x PMC2714548

[271] Berman, P., Davies, J. & Horton, R. Diabetes, obesity, and the metabolic syndrome: a call for papers for EASD and the World Diabetes Congress. *Lancet Diab Endocrinol.* **3**, 591 (2015). 10.1016/S2213-8587(15)00254-5 26144066

[272] Cipolletta, D. et al. PPAR-gamma is a major driver of the accumulation and phenotype of adipose tissue Treg cells. *Nature* **486**, 549–553 (2012). 22722857 10.1038/nature11132 PMC3387339

[273] Li, C. et al. Interferon-alpha-producing plasmacytoid dendritic cells drive the loss of adipose tissue regulatory T cells during obesity. *Cell Metab.* **33**, 1610–1623 e1615 (2021). 34256015 10.1016/j.cmet.2021.06.007 PMC8350961

[274] Ding, C. et al. A T(reg)-specific long noncoding RNA maintains immune-metabolic homeostasis in aging liver. *Nat. Aging* **3**, 813–828 (2023). 37277640 10.1038/s43587-023-00428-8

[275] Lee, J., Kim, D. & Min, B. Tissue resident Foxp3(+) regulatory T cells: sentinels and saboteurs in health and disease. *Front. Immunol.* **13**, 865593 (2022). 35359918 10.3389/fimmu.2022.865593 PMC8963273

[276] Nordestgaard, B. G. & Langsted, A. Lipoprotein(a) and cardiovascular disease. *Lancet* **404**, 1255–1264 (2024). 39278229 10.1016/S0140-6736(24)01308-4

[277] Albany, C. J. et al. Getting to the heart of the matter: the role of regulatory T-cells (Tregs) in cardiovascular disease (CVD) and atherosclerosis. *Front. Immunol.* **10**, 2795 (2019). 31849973 10.3389/fimmu.2019.02795 PMC6894511

[278] Xia, Y. et al. Role of Treg cell subsets in cardiovascular disease pathogenesis and potential therapeutic targets. *Front. Immunol.* **15**, 1331609 (2024). 38558816 10.3389/fimmu.2024.1331609 PMC10978666

[279] Hu, W., Li, J. & Cheng, X. Regulatory T cells and cardiovascular diseases. *Chin. Med. J.* **136**, 2812–2823 (2023). 37840195 10.1097/CM9.0000000000002875 PMC10686601

[280] Kuan, R., Agrawal, D. K. & Thankam, F. G. Treg cells in atherosclerosis. *Mol. Biol. Rep.* **48**, 4897–4910 (2021). 34117978 10.1007/s11033-021-06483-x

[281] Yazdani, M. R., Khosropanah, S. & Doroudchi, M. Interleukin-17 production by CD4+CD45RO+Foxp3+ T cells in peripheral blood of patients with atherosclerosis. *Arch. Med. Sci. Atheroscler. Dis.* **4**, e215–e224 (2019). 31538127 10.5114/amsad.2019.87525 PMC6749180

[282] Collison, L. W. et al. IL-35-mediated induction of a potent regulatory T cell population. *Nat. Immunol.* **11**, 1093–1101 (2010). 20953201 10.1038/ni.1952 PMC3008395

[283] Ye, C., Yano, H., Workman, C. J. & Vignali, D. A. A. Interleukin-35: structure, function and its impact on immune-related diseases. *J. Interferon Cytokine Res.* **41**, 391–406 (2021). 34788131 10.1089/jir.2021.0147 PMC8820099

[284] Landon, B. E. et al. Differences in treatment patterns and outcomes of acute myocardial infarction for low- and high-income patients in 6 countries. *JAMA* **329**, 1088–1097 (2023). 37014339 10.1001/jama.2023.1699 PMC10074220

[285] Blue, L. et al. Effects of the million hearts model on myocardial infarctions, strokes, and medicare spending: a randomized clinical trial. *JAMA* **330**, 1437–1447 (2023). 37847273 10.1001/jama.2023.19597 PMC10582785

[286] Yang, F. et al. Propionate alleviates abdominal aortic aneurysm by modulating colonic regulatory t-cell expansion and recirculation. *JACC Basic Transl. Sci.* **7**, 934–947 (2022). 36317128 10.1016/j.jacbts.2022.05.001 PMC9617133

[287] Blanco-Dominguez, R. et al. CD69 expression on regulatory T cells protects from immune damage after myocardial infarction. *J. Clin. Investig.* **132**, e152418 (2022). 36066993 10.1172/JCI152418 PMC9621142

[288] Alshoubaki, Y. K. et al. Tregs delivered post-myocardial infarction adopt an injury-specific phenotype promoting cardiac repair via macrophages in mice. *Nat. Commun.* **15**, 6480 (2024). 39090108 10.1038/s41467-024-50806-y PMC11294480

[289] Sriranjan, R. et al. Low-dose interleukin 2 for the reduction of vascular inflammation in acute coronary syndromes (IVORY): protocol and study rationale for a randomised, double-blind, placebo-controlled, phase II clinical trial. *BMJ Open* **12**, e062602 (2022). 36207050 10.1136/bmjopen-2022-062602 PMC9558794

[290] Nayer, B. et al. Local administration of regulatory T cells promotes tissue healing. *Nat. Commun.* **15**, 7863 (2024). 39251592 10.1038/s41467-024-51353-2 PMC11383969

[291] Stucchi, A., Maspes, F., Montee-Rodrigues, E. & Fousteri, G. Engineered Treg cells: the heir to the throne of immunotherapy. *J. Autoimmun.* **144**, 102986 (2024). 36639301 10.1016/j.jaut.2022.102986

[292] Hardiman, O. et al. Amyotrophic lateral sclerosis. *Nat. Rev. Dis. Prim.* **3**, 17071 (2017). 28980624 10.1038/nrdp.2017.71

[293] Valko, K. & Ciesla, L. Amyotrophic lateral sclerosis. *Prog. Med. Chem.* **58**, 63–117 (2019). 30879475 10.1016/bs.pmch.2018.12.001

[294] Beers, D. R., Zhao, W. & Appel, S. H. The role of regulatory T lymphocytes in amyotrophic lateral sclerosis. *JAMA Neurol.* **75**, 656–658 (2018). 29507936 10.1001/jamaneurol.2018.0043

[295] Henkel, J. S. et al. Regulatory T-lymphocytes mediate amyotrophic lateral sclerosis progression and survival. *EMBO Mol. Med.* **5**, 64–79 (2013). 23143995 10.1002/emmm.201201544 PMC3569654

[296] Beers, D. R. et al. ALS patients’ regulatory T lymphocytes are dysfunctional, and correlate with disease progression rate and severity. *JCI Insight* **2**, e89530 (2017). 28289705 10.1172/jci.insight.89530 PMC5333967

[297] Yazdani, S. et al. T cell responses at diagnosis of amyotrophic lateral sclerosis predict disease progression. *Nat. Commun.* **13**, 6733 (2022). 36347843 10.1038/s41467-022-34526-9 PMC9643478

[298] Beers, D. R. et al. Endogenous regulatory T lymphocytes ameliorate amyotrophic lateral sclerosis in mice and correlate with disease progression in patients with amyotrophic lateral sclerosis. *Brain* **134**, 1293–1314 (2011). 21596768 10.1093/brain/awr074 PMC3097891

[299] Sheean, R. K. et al. Association of regulatory T-cell expansion with progression of amyotrophic lateral sclerosis: a study of humans and a transgenic mouse model. *JAMA Neurol.* **75**, 681–689 (2018). 29507931 10.1001/jamaneurol.2018.0035 PMC5885208

[300] Toomer, K. H. et al. Essential and non-overlapping IL-2Rα-dependent processes for thymic development and peripheral homeostasis of regulatory T cells. *Nat. Commun.* **10**, 1037 (2019). 30833563 10.1038/s41467-019-08960-1 PMC6399264

[301] Arenas-Ramirez, N., Woytschak, J. & Boyman, O. Interleukin-2: biology, design and application. *Trends Immunol.* **36**, 763–777 (2015). 26572555 10.1016/j.it.2015.10.003

[302] Harris, F., Berdugo, Y. A. & Tree, T. IL-2-based approaches to Treg enhancement. *Clin. Exp. Immunol.* **211**, 149–163 (2023). 36399073 10.1093/cei/uxac105 PMC10019135

[303] Ito, S. et al. Ultra-low dose interleukin-2 promotes immune-modulating function of regulatory T cells and natural killer cells in healthy volunteers. *Mol. Ther.* **22**, 1388–1395 (2014). 24686272 10.1038/mt.2014.50 PMC4089007

[304] Hartemann, A. et al. Low-dose interleukin 2 in patients with type 1 diabetes: a phase 1/2 randomised, double-blind, placebo-controlled trial. *Lancet Diab Endocrinol.* **1**, 295–305 (2013). 10.1016/S2213-8587(13)70113-X 24622415

[305] Humrich, J. Y. & Riemekasten, G. Low-dose interleukin-2 therapy for the treatment of systemic lupus erythematosus. *Curr. Opin. Rheumatol.* **31**, 208–212 (2019). 30562181 10.1097/BOR.0000000000000575

[306] He, J. et al. Efficacy and safety of low-dose IL-2 in the treatment of systemic lupus erythematosus: a randomised, double-blind, placebo-controlled trial. *Ann. Rheum. Dis.* **79**, 141–149 (2020). 31537547 10.1136/annrheumdis-2019-215396 PMC6937406

[307] Duffy, S. S. et al. Regulatory T cells and their derived cytokine, interleukin-35, reduce pain in experimental autoimmune encephalomyelitis. *J. Neurosci.* **39**, 2326–2346 (2019). 30651334 10.1523/JNEUROSCI.1815-18.2019 PMC6433755

[308] Thonhoff, J. R. et al. Combined regulatory T-lymphocyte and IL-2 treatment is safe, tolerable, and biologically active for 1 year in persons with amyotrophic lateral sclerosis. *Neurol. Neuroimmunol. Neuroinflammation* **9**, e200019 (2022). 10.1212/NXI.0000000000200019 PMC9423710 36038262

[309] Camu, W. et al. Repeated 5-day cycles of low dose aldesleukin in amyotrophic lateral sclerosis (IMODALS): A phase 2a randomised, double-blind, placebo-controlled trial. *EBioMedicine* **59**, 102844 (2020). 32651161 10.1016/j.ebiom.2020.102844 PMC7502670

[310] Giovannelli, I. et al. Amyotrophic lateral sclerosis transcriptomics reveals immunological effects of low-dose interleukin-2. *Brain Commun.* **3**, fcab141 (2021). 34409288 10.1093/braincomms/fcab141 PMC8364666

[311] Scheltens, P. et al. Alzheimer’s disease. *Lancet* **397**, 1577–1590 (2021). 33667416 10.1016/S0140-6736(20)32205-4 PMC8354300

[312] Scheltens, P. et al. Alzheimer’s disease. *Lancet* **388**, 505–517 (2016). 26921134 10.1016/S0140-6736(15)01124-1

[313] Sun, L. et al. Decreased Netrin-1 and correlated Th17/Tregs balance disorder in Aβ(1-42) induced Alzheimer’s disease model rats. *Front. Aging Neurosci.* **11**, 124 (2019). 31191297 10.3389/fnagi.2019.00124 PMC6548067

[314] Oberstein, T. J. et al. Imbalance of circulating T(h)17 and regulatory T cells in Alzheimer’s disease: a case control study. *Front. Immunol.* **9**, 1213 (2018). 29915582 10.3389/fimmu.2018.01213 PMC5994416

[315] Jafarzadeh, A., Sheikhi, A., Jafarzadeh, Z. & Nemati, M. Differential roles of regulatory T cells in Alzheimer’s disease. *Cell Immunol.* **393-394**, 104778 (2023). 37907046 10.1016/j.cellimm.2023.104778

[316] Machhi, J. et al. CD4+ effector T cells accelerate Alzheimer’s disease in mice. *J. Neuroinflammation* **18**, 272 (2021). 34798897 10.1186/s12974-021-02308-7 PMC8603581

[317] Dansokho, C. et al. Regulatory T cells delay disease progression in Alzheimer-like pathology. *Brain* **139**, 1237–1251 (2016). 26912648 10.1093/brain/awv408

[318] Jung, M. et al. A therapeutic nanovaccine that generates anti-amyloid antibodies and amyloid-specific regulatory T cells for Alzheimer’s disease. *Adv. Mater.* **35**, e2207719 (2023). 36329674 10.1002/adma.202207719

[319] Elbaz, A., Carcaillon, L., Kab, S. & Moisan, F. Epidemiology of Parkinson’s disease. *Rev. Neurol.* **172**, 14–26 (2016). 26718594 10.1016/j.neurol.2015.09.012

[320] Feigin, V. L. et al. Global, regional, and national burden of neurological disorders, 1990–2016: a systematic analysis for the Global Burden of Disease Study 2016. *Lancet Neurol*. **18**, 459-480, (2019). 10.1016/S1474-4422(18)30499-X PMC6459001 30879893

[321] Savica, R. et al. Time Trends in the Incidence of Parkinson Disease. *JAMA Neurol.* **73**, 981–989 (2016). 27323276 10.1001/jamaneurol.2016.0947 PMC5004732

[322] Chung, E. S. et al. Neuro-protective effects of bee venom by suppression of neuroinflammatory responses in a mouse model of Parkinson’s disease: role of regulatory T cells. *Brain Behav. Immun.* **26**, 1322–1330 (2012). 22974722 10.1016/j.bbi.2012.08.013

[323] Duffy, S. S., Keating, B. A., Perera, C. J. & Moalem-Taylor, G. The role of regulatory T cells in nervous system pathologies. *J. Neurosci. Res.* **96**, 951–968 (2018). 28488363 10.1002/jnr.24073

[324] Huang, Y. et al. Treg cells attenuate neuroinflammation and protect neurons in a mouse model of Parkinson’s disease. *J. Neuroimmune Pharm.* **15**, 224–237 (2020). 10.1007/s11481-019-09888-5 31802419

[325] Ip, C. W. & Wischhusen, J. Versatile guardians: regenerative regulatory T cells in Parkinson’s disease rodent models. *Signal Transduct. Target. Ther.* **8**, 430 (2023). 37981647 10.1038/s41392-023-01681-4 PMC10658175

[326] Thome, A. D. et al. Ex vivo expansion of dysfunctional regulatory T lymphocytes restores suppressive function in Parkinson’s disease. *NPJ Parkinsons Dis.* **7**, 41 (2021). 33986285 10.1038/s41531-021-00188-5 PMC8119976

[327] Olson, K. E. et al. Safety, tolerability, and immune-biomarker profiling for year-long sargramostim treatment of Parkinson’s disease. *EBioMedicine* **67**, 103380 (2021). 34000620 10.1016/j.ebiom.2021.103380 PMC8138485

[328] Gendelman, H. E. et al. Evaluation of the safety and immunomodulatory effects of sargramostim in a randomized, double-blind phase 1 clinical Parkinson’s disease trial. *NPJ Parkinsons Dis.* **3**, 10 (2017). 28649610 10.1038/s41531-017-0013-5 PMC5445595

[329] Saunders, J. A. et al. CD4+ regulatory and effector/memory T cell subsets profile motor dysfunction in Parkinson’s disease. *J. Neuroimmune Pharm.* **7**, 927–938 (2012). 10.1007/s11481-012-9402-z PMC3515774 23054369

[330] Reynolds, A. D. et al. Regulatory T cells attenuate Th17 cell-mediated nigrostriatal dopaminergic neurodegeneration in a model of Parkinson’s disease. *J. Immunol.* **184**, 2261–2271 (2010). 20118279 10.4049/jimmunol.0901852 PMC2824790

[331] Takahashi, T. & Sakaguchi, S. Naturally arising CD25+CD4+ regulatory T cells in maintaining immunologic self-tolerance and preventing autoimmune disease. *Curr. Mol. Med.* **3**, 693–706 (2003). 14682491 10.2174/1566524033479429

[332] Parmar, M., Grealish, S. & Henchcliffe, C. The future of stem cell therapies for Parkinson disease. *Nat. Rev. Neurosci.* **21**, 103–115 (2020). 31907406 10.1038/s41583-019-0257-7

[333] Barker, R. A., Drouin-Ouellet, J. & Parmar, M. Cell-based therapies for Parkinson disease—past insights and future potential. *Nat. Rev. Neurol.* **11**, 492–503 (2015). 26240036 10.1038/nrneurol.2015.123

[334] Lindvall, O. Clinical translation of stem cell transplantation in Parkinson’s disease. *J. Intern. Med.* **279**, 30–40 (2016). 26332959 10.1111/joim.12415

[335] Wenker, S. D. & Pitossi, F. J. Cell therapy for Parkinson’s disease is coming of age: current challenges and future prospects with a focus on immunomodulation. *Gene Ther.* **27**, 6–14 (2020). 30992523 10.1038/s41434-019-0077-4

[336] Brundin, P. et al. Improving the survival of grafted dopaminergic neurons: a review over current approaches. *Cell Transpl.* **9**, 179–195 (2000). 10.1177/096368970000900205 10811392

[337] Park, T. Y. et al. Co-transplantation of autologous T(reg) cells in a cell therapy for Parkinson’s disease. *Nature* **619**, 606–615 (2023). 37438521 10.1038/s41586-023-06300-4 PMC12012854

[338] Muckenhuber, M., Wekerle, T. & Schwarz, C. Costimulation blockade and Tregs in solid organ transplantation. *Front Immunol.* **13**, 969633 (2022). 36119115 10.3389/fimmu.2022.969633 PMC9478950

[339] Huang, D. L. et al. The immunomodulation role of Th17 and Treg in renal transplantation. *Front. Immunol.* **14**, 1113560 (2023). 36817486 10.3389/fimmu.2023.1113560 PMC9928745

[340] Ni, X., Wang, Q., Gu, J. & Lu, L. Clinical and basic research progress on Treg-induced immune tolerance in liver transplantation. *Front. Immunol.* **12**, 535012 (2021). 34093514 10.3389/fimmu.2021.535012 PMC8173171

[341] Fisher, J. D. et al. Treg-inducing microparticles promote donor-specific tolerance in experimental vascularized composite allotransplantation. *Proc. Natl. Acad. Sci. USA* **116**, 25784–25789 (2019). 31792185 10.1073/pnas.1910701116 PMC6925993

[342] Juneja, T., Kazmi, M., Mellace, M. & Saidi, R. F. Utilization of Treg cells in solid organ transplantation. *Front. Immunol.* **13**, 746889 (2022). 35185868 10.3389/fimmu.2022.746889 PMC8854209

[343] Mandapathil, M. et al. Generation and accumulation of immunosuppressive adenosine by human CD4+CD25highFOXP3+ regulatory T cells. *J. Biol. Chem.* **285**, 7176–7186 (2010). 19858205 10.1074/jbc.M109.047423 PMC2844167

[344] Yamada, Y. et al. Biased IL-2 signals induce Foxp3-rich pulmonary lymphoid structures and facilitate long-term lung allograft acceptance in mice. *Nat. Commun.* **14**, 1383 (2023). 36914624 10.1038/s41467-023-36924-z PMC10011523

[345] Wagner, J. C. et al. Anti-HLA-A2-CAR Tregs prolong vascularized mouse heterotopic heart allograft survival. *Am. J. Transpl.* **22**, 2237–2245 (2022). 10.1111/ajt.17063 PMC9427704 35434896

[346] Todo, S. et al. A pilot study of operational tolerance with a regulatory T-cell-based cell therapy in living donor liver transplantation. *Hepatology* **64**, 632–643 (2016). 26773713 10.1002/hep.28459

[347] Cabello-Kindelan, C. et al. Immunomodulation followed by antigen-specific T(reg) infusion controls islet autoimmunity. *Diabetes* **69**, 215–227 (2020). 31712320 10.2337/db19-0061 PMC6971488

[348] Matsuoka, K. et al. Altered regulatory T cell homeostasis in patients with CD4+ lymphopenia following allogeneic hematopoietic stem cell transplantation. *J. Clin. Investig.* **120**, 1479–1493 (2010). 20389017 10.1172/JCI41072 PMC2860902

[349] Guo, W. W. et al. Regulatory T cells in GVHD therapy. *Front. Immunol.* **12**, 697854 (2021). 34220860 10.3389/fimmu.2021.697854 PMC8250864

[350] Kennedy-Nasser, A. A. et al. Ultra low-dose IL-2 for GVHD prophylaxis after allogeneic hematopoietic stem cell transplantation mediates expansion of regulatory T cells without diminishing antiviral and antileukemic activity. *Clin. Cancer Res.* **20**, 2215–2225 (2014). 24573552 10.1158/1078-0432.CCR-13-3205 PMC3989436

[351] Ramos, T. L. et al. Prevention of acute GVHD using an orthogonal IL-2/IL-2Rbeta system to selectively expand regulatory T cells in vivo. *Blood* **141**, 1337–1352 (2023). 36564052 10.1182/blood.2022018440 PMC10082364

[352] Lohmeyer, J. K. et al. Analysis of the T-cell repertoire and transcriptome identifies mechanisms of regulatory T-cell suppression of GVHD. *Blood* **141**, 1755–1767 (2023). 36574344 10.1182/blood.2022017982 PMC13122327

[353] Thiolat, A. et al. Treg-targeted IL-2/anti-IL-2 complex controls graft-versus-host disease and supports anti-tumor effect in allogeneic hematopoietic stem cell transplantation. *Haematologica* **109**, 129–142 (2024). 37706355 10.3324/haematol.2022.282653 PMC10772500

[354] Hippen, K. L. et al. Distinct regulatory and effector T cell metabolic demands during graft-versus-host disease. *Trends Immunol.* **41**, 77–91 (2020). 31791718 10.1016/j.it.2019.11.005 PMC6934920

[355] Yang, S. J. et al. Pancreatic islet-specific engineered T(regs) exhibit robust antigen-specific and bystander immune suppression in type 1 diabetes models. *Sci. Transl. Med.* **14**, eabn1716 (2022). 36197963 10.1126/scitranslmed.abn1716

[356] Bittner, S., Hehlgans, T. & Feuerer, M. Engineered Treg cells as putative therapeutics against inflammatory diseases and beyond. *Trends Immunol.* **44**, 468–483 (2023). 37100644 10.1016/j.it.2023.04.005

[357] Sakaguchi, S. et al. Regulatory T cells and human disease. *Annu. Rev. Immunol.* **38**, 541–566 (2020). 32017635 10.1146/annurev-immunol-042718-041717

[358] Cobbold, S. P. & Li, X. C. Translating tolerogenic therapies to the clinic—where do we stand and what are the barriers?. *Front Immunol.* **3**, 317 (2012). 23091475 10.3389/fimmu.2012.00317 PMC3469784

[359] Togashi, Y., Shitara, K. & Nishikawa, H. Regulatory T cells in cancer immunosuppression—implications for anticancer therapy. *Nat. Rev. Clin. Oncol.* **16**, 356–371 (2019). 30705439 10.1038/s41571-019-0175-7

[360] Edner, N. M., Carlesso, G., Rush, J. S. & Walker, L. S. K. Publisher Correction: Targeting co-stimulatory molecules in autoimmune disease. *Nat. Rev. Drug Discov.* **20**, 82 (2021). 33208921 10.1038/s41573-020-00116-x

[361] Pauken, K. E. & Wherry, E. J. Overcoming T cell exhaustion in infection and cancer. *Trends Immunol.* **36**, 265–276 (2015). 25797516 10.1016/j.it.2015.02.008 PMC4393798

[362] Croft, M. Control of immunity by the TNFR-related molecule OX40 (CD134). *Annu. Rev. Immunol.* **28**, 57–78 (2010). 20307208 10.1146/annurev-immunol-030409-101243 PMC2882161

[363] Golshayan, D. et al. In vitro-expanded donor alloantigen-specific CD4+CD25+ regulatory T cells promote experimental transplantation tolerance. *Blood* **109**, 827–835 (2007). 17003369 10.1182/blood-2006-05-025460

[364] Pereira, J. A. et al. PD-1 and CTLA-4 exert additive control of effector regulatory T cells at homeostasis. *Front. Immunol.* **14**, 997376 (2023). 36960049 10.3389/fimmu.2023.997376 PMC10028286

[365] Marangoni, F. et al. Expansion of tumor-associated Treg cells upon disruption of a CTLA-4-dependent feedback loop. *Cell* **184**, 3998–4015 e3919 (2021). 34157302 10.1016/j.cell.2021.05.027 PMC8664158

[366] Tekguc, M. et al. Treg-expressed CTLA-4 depletes CD80/CD86 by trogocytosis, releasing free PD-L1 on antigen-presenting cells. *Proc. Natl. Acad. Sci. USA* **118**, e2023739118 (2021). 10.1073/pnas.2023739118 PMC8325248 34301886

[367] Zappasodi, R. et al. CTLA-4 blockade drives loss of T(reg) stability in glycolysis-low tumours. *Nature* **591**, 652–658 (2021). 33588426 10.1038/s41586-021-03326-4 PMC8057670

[368] Simpson, T. R. et al. Fc-dependent depletion of tumor-infiltrating regulatory T cells co-defines the efficacy of anti-CTLA-4 therapy against melanoma. *J. Exp. Med.* **210**, 1695–1710 (2013). 23897981 10.1084/jem.20130579 PMC3754863

[369] Ha, D. et al. Differential control of human Treg and effector T cells in tumor immunity by Fc-engineered anti-CTLA-4 antibody. *Proc. Natl. Acad. Sci. USA* **116**, 609–618 (2019). 30587582 10.1073/pnas.1812186116 PMC6329979

[370] John, P. et al. The immune checkpoint B7x expands tumor-infiltrating Tregs and promotes resistance to anti-CTLA-4 therapy. *Nat. Commun.* **13**, 2506 (2022). 35523809 10.1038/s41467-022-30143-8 PMC9076640

[371] Knorr, D. A. et al. FcgammaRIIB is an immune checkpoint limiting the activity of Treg-targeting antibodies in the tumor microenvironment. *Cancer Immunol. Res.* **12**, 322–333 (2024). 38147316 10.1158/2326-6066.CIR-23-0389 PMC10911703

[372] Shan, F. et al. Therapeutic targeting of regulatory T cells in cancer. *Trends Cancer* **8**, 944–961 (2022). 35853825 10.1016/j.trecan.2022.06.008 PMC9588644

[373] Crepeau, R. L. & Ford, M. L. Challenges and opportunities in targeting the CD28/CTLA-4 pathway in transplantation and autoimmunity. *Expert Opin. Biol. Ther.* **17**, 1001–1012 (2017). 28525959 10.1080/14712598.2017.1333595 PMC5590720

[374] Bano, A. et al. CD28 (null) CD4 T-cell expansions in autoimmune disease suggest a link with cytomegalovirus infection. *F1000Research* **8**, F1000-aculty (2019). 10.12688/f1000research.17119.1 PMC6436193 30984377

[375] Liu, D., Badell, I. R. & Ford, M. L. Selective CD28 blockade attenuates CTLA-4-dependent CD8+ memory T cell effector function and prolongs graft survival. *JCI Insight*. **3**, e96378 (2018). 10.1172/jci.insight.96378 PMC5821191 29321374

[376] Hossen, M. M. et al. Current understanding of CTLA-4: from mechanism to autoimmune diseases. *Front. Immunol.* **14**, 1198365 (2023). 37497212 10.3389/fimmu.2023.1198365 PMC10367421

[377] Cope, A. P. et al. Abatacept in individuals at high risk of rheumatoid arthritis (APIPPRA): a randomised, double-blind, multicentre, parallel, placebo-controlled, phase 2b clinical trial. *Lancet* **403**, 838–849 (2024). 38364839 10.1016/S0140-6736(23)02649-1

[378] Budde, K. et al. Conversion from calcineurin inhibitor—to belatacept-based maintenance immunosuppression in renal transplant recipients: a randomized phase 3b trial. *J. Am. Soc. Nephrol.* **32**, 3252–3264 (2021). 34706967 10.1681/ASN.2021050628 PMC8638403

[379] Liu, B. et al. Temporal single-cell tracing reveals clonal revival and expansion of precursor exhausted T cells during anti-PD-1 therapy in lung cancer. *Nat. Cancer* **3**, 108–121 (2022). 35121991 10.1038/s43018-021-00292-8

[380] Champiat, S. et al. Hyperprogressive disease: recognizing a novel pattern to improve patient management. *Nat. Rev. Clin. Oncol.* **15**, 748–762 (2018). 30361681 10.1038/s41571-018-0111-2

[381] Wu, Q. et al. Targeting neuropilin-1 abolishes anti-PD-1-upregulated regulatory T cells and synergizes with 4-1BB agonist for liver cancer treatment. *Hepatology* **78**, 1402–1417 (2023). 36811396 10.1097/HEP.0000000000000320

[382] Kumagai, S. et al. Lactic acid promotes PD-1 expression in regulatory T cells in highly glycolytic tumor microenvironments. *Cancer Cell* **40**, 201–218 e209 (2022). 35090594 10.1016/j.ccell.2022.01.001

[383] van Gulijk, M. et al. PD-L1 checkpoint blockade promotes regulatory T cell activity that underlies therapy resistance. *Sci. Immunol.* **8**, eabn6173 (2023). 37205768 10.1126/sciimmunol.abn6173

[384] Adamczyk, M. & Krasowska, D. PD1/PD-L1 pathway in psoriasis and psoriatic arthritis: a review. *Postepy Dermatol. Alergol.* **38**, 925–930 (2021). 35125995 10.5114/ada.2021.112274 PMC8802966

[385] Borges, T. J. et al. Overexpression of PD-1 on T cells promotes tolerance in cardiac transplantation via ICOS-dependent mechanisms. *JCI Insight*. **6**, e142909 (2021). 10.1172/jci.insight.142909 PMC8783692 34752418

[386] Liang, Y. et al. Blockade of PD-1/PD-L1 increases effector T cells and aggravates murine chronic graft-versus-host disease. *Int. Immunopharmacol.* **110**, 109051 (2022). 35850051 10.1016/j.intimp.2022.109051

[387] Lim, S. A. et al. Lipid signalling enforces functional specialization of T(reg) cells in tumours. *Nature* **591**, 306–311 (2021). 33627871 10.1038/s41586-021-03235-6 PMC8168716

[388] Ferreira, L. M. R., Muller, Y. D., Bluestone, J. A. & Tang, Q. Next-generation regulatory T cell therapy. *Nat. Rev. Drug Discov.* **18**, 749–769 (2019). 31541224 10.1038/s41573-019-0041-4 PMC7773144

[389] Guan, X. et al. Anti-TIGIT antibody improves PD-L1 blockade through myeloid and T(reg) cells. *Nature* **627**, 646–655 (2024). 38418879 10.1038/s41586-024-07121-9 PMC11139643

[390] Yue, C. et al. TIGIT as a promising therapeutic target in autoimmune diseases. *Front. Immunol.* **13**, 911919 (2022). 35720417 10.3389/fimmu.2022.911919 PMC9203892

[391] Guo, Q. et al. Engineered PD-1/TIGIT dual-activating cell-membrane nanoparticles with dexamethasone act synergistically to shape the effector T cell/Treg balance and alleviate systemic lupus erythematosus. *Biomaterials* **285**, 121517 (2022). 35504179 10.1016/j.biomaterials.2022.121517

[392] Heiduk, M. et al. TIGIT expression delineates T-cell populations with distinct functional and prognostic impact in pancreatic cancer. *Clin. Cancer Res.* **29**, 2638–2650 (2023). 37140899 10.1158/1078-0432.CCR-23-0258 PMC10345964

[393] Chauvin, J. M. et al. IL15 Stimulation with TIGIT blockade reverses CD155-mediated NK-cell dysfunction in melanoma. *Clin. Cancer Res.* **26**, 5520–5533 (2020). 32591463 10.1158/1078-0432.CCR-20-0575 PMC8045409

[394] Fourcade, J. et al. CD226 opposes TIGIT to disrupt Tregs in melanoma. *JCI Insight*. **3**, e121157 (2018). 10.1172/jci.insight.121157 PMC6124410 30046006

[395] Selby, M. J. et al. Anti-CTLA-4 antibodies of IgG2a isotype enhance antitumor activity through reduction of intratumoral regulatory T cells. *Cancer Immunol. Res.* **1**, 32–42 (2013). 24777248 10.1158/2326-6066.CIR-13-0013

[396] Arce et al. Fc effector function contributes to the activity of human anti-CTLA-4 antibodies. *Cancer Cell* **33**, 649–663 e644 (2018). 29576375 10.1016/j.ccell.2018.02.010 PMC5904288

[397] Gedaly, R. et al. Metabolic disruption induced by mTOR signaling pathway inhibition in regulatory T-cell expansion for clinical application. *Cells* **12**, 2066 (2023). 37626877 10.3390/cells12162066 PMC10453008

[398] Rudin, C. M. et al. SKYSCRAPER-02: tiragolumab in combination with atezolizumab plus chemotherapy in untreated extensive-stage small-cell lung cancer. *J. Clin. Oncol.* **42**, 324–335 (2024). 37976444 10.1200/JCO.23.01363 PMC10824371

[399] Bae, J. et al. Targeting LAG3/GAL-3 to overcome immunosuppression and enhance anti-tumor immune responses in multiple myeloma. *Leukemia* **36**, 138–154 (2022). 34290359 10.1038/s41375-021-01301-6 PMC8727303

[400] Mulholland, M. et al. LAG3 Regulates T cell activation and plaque infiltration in atherosclerotic mice. *JACC CardioOncol* **4**, 635–645 (2022). 36636446 10.1016/j.jaccao.2022.09.005 PMC9830219

[401] Aggarwal, V., Workman, C. J. & Vignali, D. A. A. LAG-3 as the third checkpoint inhibitor. *Nat. Immunol.* **24**, 1415–1422 (2023). 37488429 10.1038/s41590-023-01569-z PMC11144386

[402] Chocarro, L. et al. Understanding LAG-3 signaling. *Int. J. Mol. Sci*. **22**, 5282 (2021). 10.3390/ijms22105282 PMC8156499 34067904

[403] Huo, J. L. et al. The promising immune checkpoint LAG-3 in cancer immunotherapy: from basic research to clinical application. *Front. Immunol.* **13**, 956090 (2022). 35958563 10.3389/fimmu.2022.956090 PMC9361790

[404] Grebinoski, S. et al. Autoreactive CD8(+) T cells are restrained by an exhaustion-like program that is maintained by LAG3. *Nat. Immunol.* **23**, 868–877 (2022). 35618829 10.1038/s41590-022-01210-5 PMC9179227

[405] Jones, B. E. et al. Fewer LAG-3(+) T cells in relapsing-remitting multiple sclerosis and type 1 diabetes. *J. Immunol.* **208**, 594–602 (2022). 35022272 10.4049/jimmunol.2100850 PMC8820445

[406] Garcia-Martin, E. et al. Association between LAG3/CD4 genes variants and risk for multiple sclerosis. *Int J. Mol. Sci.* **23**, 15244 (2022). 36499569 10.3390/ijms232315244 PMC9735634

[407] Saevarsdottir, S. et al. Start codon variant in LAG3 is associated with decreased LAG-3 expression and increased risk of autoimmune thyroid disease. *Nat. Commun.* **15**, 5748 (2024). 38982041 10.1038/s41467-024-50007-7 PMC11233504

[408] Qureshi, F. M. et al. Immunotherapy with low-dose IL-2/CD25 prevents beta-cell dysfunction and dysglycemia in prediabetic NOD mice. *Diabetes* **72**, 769–780 (2023). 36939730 10.2337/db22-0482 PMC10202767

[409] Ren, Z. et al. Selective delivery of low-affinity IL-2 to PD-1+ T cells rejuvenates antitumor immunity with reduced toxicity. *J. Clin. Investig*. **132**, e153604 (2022). 10.1172/JCI153604 PMC8803347 35104810

[410] Dixit, N. et al. NKTR-358: a novel regulatory T-cell stimulator that selectively stimulates expansion and suppressive function of regulatory T cells for the treatment of autoimmune and inflammatory diseases. *J. Transl. Autoimmun*. **4**, 100103 (2021). 10.1016/j.jtauto.2021.100103 PMC8141531 34041473

[411] Karpisheh, V. et al. The role of regulatory T cells in the pathogenesis and treatment of prostate cancer. *Life Sci.* **284**, 119132 (2021). 33513396 10.1016/j.lfs.2021.119132

[412] Zammarchi, F. et al. CD25-targeted antibody-drug conjugate depletes regulatory T cells and eliminates established syngeneic tumors via antitumor immunity. *J. Immunother. Cancer*. **8**, e000860 (2020). 10.1136/jitc-2020-000860 PMC7482493 32912922

[413] Villanueva, M. T. Anti-CD25 antibody tips the T cell balance. *Nat. Rev. Drug Discov.* **20**, 18 (2021). 33247220 10.1038/d41573-020-00206-w

[414] Arce et al. Fc-optimized anti-CD25 depletes tumor-infiltrating regulatory T cells and synergizes with PD-1 blockade to eradicate established tumors. *Immunity* **46**, 577–586 (2017). 28410988 10.1016/j.immuni.2017.03.013 PMC5437702

[415] Buzzatti, G., Dellepiane, C. & Del Mastro, L. New emerging targets in cancer immunotherapy: the role of GITR. *ESMO Open* **4**, e000738 (2020). 32817129 10.1136/esmoopen-2020-000738 PMC7451269

[416] Sun, J. et al. Aberrant GITR expression on different T cell subsets and the regulation by glucocorticoid in systemic lupus erythematosus. *Int. J. Rheum. Dis.* **19**, 199–204 (2016). 25293713 10.1111/1756-185X.12451

[417] Hilaire, M. & Aubert, N. Boosting Treg activity by TNFR2 and GITR agonists: new therapeutic approaches for autoimmune diseases. *Med. Sci.* **35**, 702–705 (2019). 10.1051/medsci/2019138 31532385

[418] Tian, J., Zhang, B., Rui, K. & Wang, S. The role of GITR/GITRL interaction in autoimmune diseases. *Front Immunol.* **11**, 588682 (2020). 33163004 10.3389/fimmu.2020.588682 PMC7581784

[419] Kohm, A. P., Williams, J. S. & Miller, S. D. Cutting edge: ligation of the glucocorticoid-induced TNF receptor enhances autoreactive CD4+ T cell activation and experimental autoimmune encephalomyelitis. *J. Immunol.* **172**, 4686–4690 (2004). 15067043 10.4049/jimmunol.172.8.4686

[420] Sun, Q. et al. Phototherapy and anti-GITR antibody-based therapy synergistically reinvigorate immunogenic cell death and reject established cancers. *Biomaterials* **269**, 120648 (2021). 33445099 10.1016/j.biomaterials.2020.120648

[421] Schaer, D. A. et al. GITR pathway activation abrogates tumor immune suppression through loss of regulatory T cell lineage stability. *Cancer Immunol. Res.* **1**, 320–331 (2013). 24416730 10.1158/2326-6066.CIR-13-0086 PMC3885345

[422] Zappasodi, R. et al. Rational design of anti-GITR-based combination immunotherapy. *Nat. Med.* **25**, 759–766 (2019). 31036879 10.1038/s41591-019-0420-8 PMC7457830

[423] Sathe, A. et al. GITR and TIGIT immunotherapy provokes divergent multi-cellular responses in the tumor microenvironment of gastrointestinal cancers. *Genome Med.* **15**, 100 (2023). 38008725 10.1186/s13073-023-01259-3 PMC10680277

[424] Ke, S. et al. High-level of intratumoral GITR+ CD4 T cells associate with poor prognosis in gastric cancer. *iScience* **25**, 105529 (2022). 36419848 10.1016/j.isci.2022.105529 PMC9676631

[425] Ji, Y. et al. Quantitative systems pharmacology model of GITR-mediated T cell dynamics in tumor microenvironment. *CPT Pharmacomet. Syst. Pharm.* **12**, 413–424 (2023). 10.1002/psp4.12925 PMC10014051 36710369

[426] Ward-Kavanagh, L. K., Lin, W. W., Sedy, J. R. & Ware, C. F. The TNF receptor superfamily in co-stimulating and co-inhibitory responses. *Immunity* **44**, 1005–1019 (2016). 27192566 10.1016/j.immuni.2016.04.019 PMC4882112

[427] Kumar, P. et al. Critical role of OX40 signaling in the TCR-independent phase of human and murine thymic Treg generation. *Cell. Mol. Immunol.* **16**, 138–153 (2019). 29578532 10.1038/cmi.2018.8 PMC6355936

[428] Kurata, I. et al. Potential involvement of OX40 in the regulation of autoantibody sialylation in arthritis. *Ann. Rheum. Dis.* **78**, 1488–1496 (2019). 31300460 10.1136/annrheumdis-2019-215195

[429] Zhou, X. et al. Clinical significance of OX40 and OX40 ligand in the peripheral blood of patients with myasthenia gravis. *J. Immunol. Res.* **2022**, 4337399 (2022). 35265719 10.1155/2022/4337399 PMC8901326

[430] Iriki, H., Takahashi, H. & Amagai, M. Diverse role of OX40 on T cells as a therapeutic target for skin diseases. *J. Investig. Dermatol.* **143**, 545–553 (2023). 36842860 10.1016/j.jid.2022.11.009

[431] Pacella, I. et al. Fatty acid metabolism complements glycolysis in the selective regulatory T cell expansion during tumor growth. *Proc. Natl. Acad. Sci. USA* **115**, E6546–E6555 (2018). 29941600 10.1073/pnas.1720113115 PMC6048537

[432] Oberst, M. D. et al. Potent immune modulation by MEDI6383, an engineered human OX40 ligand IgG4P Fc fusion protein. *Mol. Cancer Ther.* **17**, 1024–1038 (2018). 29545330 10.1158/1535-7163.MCT-17-0200 PMC5932227

[433] Knisely, A. et al. Phase 1/2 trial of avelumab combined with utomilumab (4-1BB agonist), PF-04518600 (OX40 agonist), or radiotherapy in patients with advanced gynecologic malignancies. *Cancer* **130**, 400–409 (2024). 37864520 10.1002/cncr.35063 PMC10841432

[434] Liang, S. et al. BAT6026, a novel anti-OX40 antibody with enhanced antibody dependent cellular cytotoxicity effect for cancer immunotherapy. *Front. Oncol.* **13**, 1211759 (2023). 37576888 10.3389/fonc.2023.1211759 PMC10421724

[435] Davis, E. J. et al. First-in-human phase I/II, open-label study of the anti-OX40 agonist INCAGN01949 in patients with advanced solid tumors. *J. Immunother. Cancer* **10**, e004235 (2022). 36316061 10.1136/jitc-2021-004235 PMC9628691

[436] Duhen, R. et al. Neoadjuvant anti-OX40 (MEDI6469) therapy in patients with head and neck squamous cell carcinoma activates and expands antigen-specific tumor-infiltrating T cells. *Nat. Commun.* **12**, 1047 (2021). 33594075 10.1038/s41467-021-21383-1 PMC7886909

[437] Knoechel, B. et al. Sequential development of interleukin 2-dependent effector and regulatory T cells in response to endogenous systemic antigen. *J. Exp. Med.* **202**, 1375–1386 (2005). 16287710 10.1084/jem.20050855 PMC2212975

[438] Alves Costa Silva, C., Facchinetti, F., Routy, B. & Derosa, L. New pathways in immune stimulation: targeting OX40. *ESMO Open*. **5**, e000573 (2020). 10.1136/esmoopen-2019-000573 PMC7046367 32392177

[439] Herman, A. E., Freeman, G. J., Mathis, D. & Benoist, C. CD4+CD25+ T regulatory cells dependent on ICOS promote regulation of effector cells in the prediabetic lesion. *J. Exp. Med.* **199**, 1479–1489 (2004). 15184501 10.1084/jem.20040179 PMC2211778

[440] Landuyt, A. E. et al. Cutting edge: ICOS-deficient regulatory T cells display normal induction of Il10 but readily downregulate expression of Foxp3. *J. Immunol.* **202**, 1039–1044 (2019). 30642977 10.4049/jimmunol.1801266 PMC6363853

[441] Whitehead, G. S. et al. IL-35 production by inducible costimulator (ICOS)-positive regulatory T cells reverses established IL-17-dependent allergic airways disease. *J. Allergy Clin. Immunol.* **129**, 207–215 (2012). e201-205. 21906794 10.1016/j.jaci.2011.08.009 PMC3269135

[442] Li, D. Y. & Xiong, X. Z. Corrigendum: ICOS+ Tregs: a functional subset of tregs in immune diseases. *Front Immunol.* **12**, 701515 (2021). 34054885 10.3389/fimmu.2021.701515 PMC8149802

[443] Borgeaud, M. et al. Novel targets for immune-checkpoint inhibition in cancer. *Cancer Treat. Rev.* **120**, 102614 (2023). 37603905 10.1016/j.ctrv.2023.102614

[444] Fu, T., He, Q. & Sharma, P. The ICOS/ICOSL pathway is required for optimal antitumor responses mediated by anti–CTLA-4 therapy. *Cancer Res.* **71**, 5445–5454 (2011). 21708958 10.1158/0008-5472.CAN-11-1138

[445] Panneton, V. et al. ICOS costimulation is indispensable for the differentiation of T follicular regulatory cells. *Life Sci. Alliance* **6**, e202201615 (2023). 36754569 10.26508/lsa.202201615 PMC9909462

[446] Sainson, R. C. A. et al. An antibody targeting ICOS increases intratumoral cytotoxic to regulatory T-cell ratio and induces tumor regression. *Cancer Immunol. Res.* **8**, 1568–1582 (2020). 32999002 10.1158/2326-6066.CIR-20-0034

[447] Shabaneh, T. B. et al. Oncogenic BRAF(V600E) governs regulatory T-cell recruitment during melanoma tumorigenesis. *Cancer Res.* **78**, 5038–5049 (2018). 30026331 10.1158/0008-5472.CAN-18-0365 PMC6319620

[448] Yamamoto, K. et al. Phase I study of KW-0761, a defucosylated humanized anti-CCR4 antibody, in relapsed patients with adult T-cell leukemia-lymphoma and peripheral T-cell lymphoma. *J. Clin. Oncol.* **28**, 1591–1598 (2010). 20177026 10.1200/JCO.2009.25.3575

[449] Korbecki, J. et al. CC chemokines in a tumor: a review of pro-cancer and anti-cancer properties of the ligands of receptors CCR1, CCR2, CCR3, and CCR4. *Int. J. Mol. Sci.* **21**, 8412 (2020). 33182504 10.3390/ijms21218412 PMC7665155

[450] Marshall, L. A. et al. Tumors establish resistance to immunotherapy by regulating T(reg) recruitment via CCR4. *J. Immunother. Cancer* **8**, e000764 (2020). 33243932 10.1136/jitc-2020-000764 PMC7692993

[451] Plitas, G. et al. Regulatory T cells exhibit distinct features in human breast cancer. *Immunity* **45**, 1122–1134 (2016). 27851913 10.1016/j.immuni.2016.10.032 PMC5134901

[452] Campbell, J. R. et al. Fc-optimized anti-CCR8 antibody depletes regulatory T cells in human tumor models. *Cancer Res.* **81**, 2983–2994 (2021). 33757978 10.1158/0008-5472.CAN-20-3585

[453] Wu, Y. et al. Discovery of a potent and selective CCR8 small molecular antagonist IPG7236 for the treatment of cancer. *J. Med. Chem.* **66**, 4548–4564 (2023). 36988587 10.1021/acs.jmedchem.3c00030

[454] Facciabene, A. et al. Tumour hypoxia promotes tolerance and angiogenesis via CCL28 and T(reg) cells. *Nature* **475**, 226–230 (2011). 21753853 10.1038/nature10169

[455] Hui, Z. et al. Single-cell sequencing reveals the transcriptome and TCR characteristics of pTregs and in vitro expanded iTregs. *Front Immunol.* **12**, 619932 (2021). 33868236 10.3389/fimmu.2021.619932 PMC8044526

[456] Bellanti, J. A. & Li, D. Treg cells and epigenetic regulation. *Adv. Exp. Med. Biol.* **1278**, 95–114 (2021). 33523445 10.1007/978-981-15-6407-9_6

[457] Sun, X. et al. TGF-beta signaling controls Foxp3 methylation and T reg cell differentiation by modulating Uhrf1 activity. *J. Exp. Med.* **216**, 2819–2837 (2019). 31515281 10.1084/jem.20190550 PMC6888975

[458] Liu, L. et al. UHRF1 downregulation promotes T follicular helper cell differentiation by increasing BCL6 expression in SLE. *Clin. Epigenetics* **13**, 31 (2021). 33568199 10.1186/s13148-021-01007-7 PMC7874639

[459] DuPage, M. et al. The chromatin-modifying enzyme Ezh2 is critical for the maintenance of regulatory T cell identity after activation. *Immunity* **42**, 227–238 (2015). 25680271 10.1016/j.immuni.2015.01.007 PMC4347854

[460] Wang, D. et al. Targeting EZH2 reprograms intratumoral regulatory T cells to enhance cancer immunity. *Cell Rep.* **23**, 3262–3274 (2018). 29898397 10.1016/j.celrep.2018.05.050 PMC6094952

[461] Lee, J. C. et al. Regulatory T cell control of systemic immunity and immunotherapy response in liver metastasis. *Sci. Immunol*. **5**, eaba0759 (2020). 10.1126/sciimmunol.aba0759 PMC7755924 33008914

[462] Singh, V. et al. Epigenetic reprogramming of T cells: unlocking new avenues for cancer immunotherapy. *Cancer Metastasis Rev.* **43**, 175–195 (2024). 38233727 10.1007/s10555-024-10167-w

[463] Goswami, S. et al. Modulation of EZH2 expression in T cells improves efficacy of anti-CTLA-4 therapy. *J. Clin. Investig.* **128**, 3813–3818 (2018). 29905573 10.1172/JCI99760 PMC6118570

[464] Christensen, L. M. & Hancock, W. W. Nuclear coregulatory complexes in Tregs as targets to promote anticancer immune responses. *Front. Immunol.* **13**, 909816 (2022). 35795673 10.3389/fimmu.2022.909816 PMC9251111

[465] Sacristan-Gomez, P. et al. Analysis of expression of different histone deacetylases in autoimmune thyroid disease. *J. Clin. Endocrinol. Metab.* **106**, 3213–3227 (2021). 34272941 10.1210/clinem/dgab526 PMC8530745

[466] Buglio, D. et al. HDAC11 plays an essential role in regulating OX40 ligand expression in Hodgkin lymphoma. *Blood* **117**, 2910–2917 (2011). 21239696 10.1182/blood-2010-08-303701 PMC3062301

[467] Wang, L. et al. FOXP3+ regulatory T cell development and function require histone/protein deacetylase 3. *J. Clin. Investig.* **125**, 1111–1123 (2015). 25642770 10.1172/JCI77088 PMC4362235

[468] Liu, Y. et al. Complementary roles of GCN5 and PCAF in Foxp3+ T-regulatory cells. *Cancers* **11**, 554 (2019). 10.3390/cancers11040554 PMC6520961 31003455

[469] Wu, B. et al. A novel liver cancer-selective histone deacetylase inhibitor is effective against hepatocellular carcinoma and induces durable responses with immunotherapy. *ACS Pharm. Transl. Sci.* **7**, 3155–3169 (2024). 10.1021/acsptsci.4c00358 PMC11475281 39416967

[470] Tao, R. et al. Deacetylase inhibition promotes the generation and function of regulatory T cells. *Nat. Med.* **13**, 1299–1307 (2007). 17922010 10.1038/nm1652

[471] Liu, Y. et al. Two histone/protein acetyltransferases, CBP and p300, are indispensable for Foxp3+ T-regulatory cell development and function. *Mol. Cell. Biol.* **34**, 3993–4007 (2014). 25154413 10.1128/MCB.00919-14 PMC4386456

[472] de Almeida Nagata, D. E. et al. Regulation of tumor-associated myeloid cell activity by CBP/EP300 bromodomain modulation of H3K27 acetylation. *Cell Rep.* **27**, 269–281.e264 (2019). 30943407 10.1016/j.celrep.2019.03.008

[473] Feng, Y. et al. A mechanism for expansion of regulatory T-cell repertoire and its role in self-tolerance. *Nature* **528**, 132–136 (2015). 26605529 10.1038/nature16141 PMC4862833

[474] Rosado-Sanchez, I. & Levings, M. K. Building a CAR-Treg: going from the basic to the luxury model. *Cell Immunol.* **358**, 104220 (2020). 33096321 10.1016/j.cellimm.2020.104220

[475] Mannie, M. D., DeOca, K. B., Bastian, A. G. & Moorman, C. D. Tolerogenic vaccines: targeting the antigenic and cytokine niches of FOXP3(+) regulatory T cells. *Cell Immunol.* **355**, 104173 (2020). 32712270 10.1016/j.cellimm.2020.104173 PMC7444458

[476] Rana, J. & Biswas, M. Regulatory T cell therapy: current and future design perspectives. *Cell Immunol.* **356**, 104193 (2020). 32823038 10.1016/j.cellimm.2020.104193

[477] Arjomandnejad, M., Kopec, A. L. & Keeler, A. M. CAR-T regulatory (CAR-Treg) cells: engineering and applications. *Biomedicines* **10**, 287 (2022). 35203496 10.3390/biomedicines10020287 PMC8869296

[478] Fritsche, E., Volk, H. D., Reinke, P. & Abou-El-Enein, M. Toward an optimized process for clinical manufacturing of CAR-Treg cell therapy. *Trends Biotechnol.* **38**, 1099–1112 (2020). 31982150 10.1016/j.tibtech.2019.12.009

[479] Good, Z. et al. Post-infusion CAR T(Reg) cells identify patients resistant to CD19-CAR therapy. *Nat. Med.* **28**, 1860–1871 (2022). 36097223 10.1038/s41591-022-01960-7 PMC10917089

[480] Haradhvala, N. J. et al. Distinct cellular dynamics associated with response to CAR-T therapy for refractory B cell lymphoma. *Nat. Med.* **28**, 1848–1859 (2022). 36097221 10.1038/s41591-022-01959-0 PMC9509487

[481] Zhang, A. H., Yoon, J., Kim, Y. C. & Scott, D. W. Targeting antigen-specific B cells using antigen-expressing transduced regulatory T cells. *J. Immunol.* **201**, 1434–1441 (2018). 30021767 10.4049/jimmunol.1701800 PMC6103823

[482] Bulliard, Y. et al. From promise to practice: CAR T and Treg cell therapies in autoimmunity and other immune-mediated diseases. *Front. Immunol.* **15**, 1509956 (2024). 39697333 10.3389/fimmu.2024.1509956 PMC11653210

[483] Skuljec, J. et al. Chimeric antigen receptor-redirected regulatory T cells suppress experimental allergic airway inflammation, a model of asthma. *Front. Immunol.* **8**, 1125 (2017). 28955341 10.3389/fimmu.2017.01125 PMC5600908

[484] Mohammadi, V. et al. Chimeric antigen receptor (CAR)-based cell therapy for type 1 diabetes mellitus (T1DM); current progress and future approaches. *Stem Cell Rev. Rep.* **20**, 585–600 (2024). 38153634 10.1007/s12015-023-10668-1

[485] Frikeche, J. et al. MOG-specific CAR Tregs: a novel approach to treat multiple sclerosis. *J. Neuroinflammation* **21**, 268 (2024). 39428507 10.1186/s12974-024-03262-w PMC11490997

[486] Wang, S. W. et al. Current applications and future perspective of CRISPR/Cas9 gene editing in cancer. *Mol. Cancer* **21**, 57 (2022). 35189910 10.1186/s12943-022-01518-8 PMC8862238

[487] Zhai, N. et al. Lack of IFN-gamma receptor signaling inhibits graft-versus-host disease by potentiating regulatory T cell expansion and conversion. *J. Immunol.* **211**, 885–894 (2023). 37486211 10.4049/jimmunol.2200411

[488] Chen, X., Zhong, S., Zhan, Y. & Zhang, X. CRISPR-Cas9 applications in T cells and adoptive T cell therapies. *Cell Mol. Biol. Lett.* **29**, 52 (2024). 38609863 10.1186/s11658-024-00561-1 PMC11010303

[489] Van Zeebroeck, L. et al. Fast and efficient genome editing of human FOXP3(+) regulatory T cells. *Front. Immunol.* **12**, 655122 (2021). 34408743 10.3389/fimmu.2021.655122 PMC8365355

[490] Obradovic, A. et al. Systematic elucidation and pharmacological targeting of tumor-infiltrating regulatory T cell master regulators. *Cancer Cell* **41**, 933–949 e911 (2023). 37116491 10.1016/j.ccell.2023.04.003 PMC10193511

[491] Borna, S. et al. Identification of unstable regulatory and autoreactive effector T cells that are expanded in patients with FOXP3 mutations. *Sci. Transl. Med.* **15**, eadg6822 (2023). 38117899 10.1126/scitranslmed.adg6822 PMC11070150

[492] Tang, N. et al. TGF-beta inhibition via CRISPR promotes the long-term efficacy of CAR T cells against solid tumors. *JCI Insight* **5**, e133977 (2020). 10.1172/jci.insight.133977 PMC7101140 31999649

[493] Tian, H., Lyu, Y., Yang, Y. G. & Hu, Z. Humanized Rodent Models for Cancer Research. *Front Oncol.* **10**, 1696 (2020). 33042811 10.3389/fonc.2020.01696 PMC7518015

[494] Cao, Y. et al. Integrative analysis from multi-center studies identifies a weighted gene co-expression network analysis-based Tregs signature in ovarian cancer. *Environ. Toxicol*. **39**, 736–750 (2023). 10.1002/tox.23948 37713585

[495] Ou, X. et al. CRISPR/Cas9 gene-editing in cancer immunotherapy: promoting the present revolution in cancer therapy and exploring more. *Front. Cell Dev. Biol.* **9**, 674467 (2021). 34095145 10.3389/fcell.2021.674467 PMC8172808

[496] Liu, Z. et al. Recent advances and applications of CRISPR-Cas9 in cancer immunotherapy. *Mol. Cancer* **22**, 35 (2023). 36797756 10.1186/s12943-023-01738-6 PMC9933290

[497] Nguyen, D. N. et al. Polymer-stabilized Cas9 nanoparticles and modified repair templates increase genome editing efficiency. *Nat. Biotechnol.* **38**, 44–49 (2020). 31819258 10.1038/s41587-019-0325-6 PMC6954310

[498] Chen, C. N. et al. Restoration of Foxp3(+) regulatory T Cells by HDAC-dependent epigenetic modulation plays a pivotal role in resolving pulmonary arterial hypertension pathology. *Am. J. Respir. Crit. Care Med.* **208**, 879–895 (2023). 37676930 10.1164/rccm.202301-0181OC

[499] Zhang, F. et al. Involvement of CHRNA6 in the immune response in lung squamous cell carcinoma and its potential as a drug target for the disease. *Curr. Pharm. Des.* **29**, 2091–2100 (2023). 37680128 10.2174/1381612829666230901143203

[500] Liao, W., Lin, J. X. & Leonard, W. J. Interleukin-2 at the crossroads of effector responses, tolerance, and immunotherapy. *Immunity* **38**, 13–25 (2013). 23352221 10.1016/j.immuni.2013.01.004 PMC3610532

[501] Humrich, J. Y. et al. Low-dose interleukin-2 therapy in active systemic lupus erythematosus (LUPIL-2): a multicentre, double-blind, randomised and placebo-controlled phase II trial. *Ann. Rheum. Dis.* **81**, 1685–1694 (2022). 35973803 10.1136/ard-2022-222501

[502] Todd, J. A. et al. Regulatory T cell responses in participants with type 1 diabetes after a single dose of interleukin-2: a non-randomised, open label, adaptive dose-finding trial. *PLoS Med.* **13**, e1002139 (2016). 27727279 10.1371/journal.pmed.1002139 PMC5058548

[503] Wang, Z. et al. The effects of low-dose IL-2 on Th17/Treg cell imbalance in primary biliary cholangitis mouse models. *BMC Gastroenterol.* **24**, 87 (2024). 38408917 10.1186/s12876-024-03176-0 PMC10895794

[504] Allegretti, J. R. et al. Low-dose interleukin 2 for the treatment of moderate to severe ulcerative colitis. *Gastroenterology* **165**, 492–495. e492 (2023). 37030335 10.1053/j.gastro.2023.03.230 PMC10523991

[505] Zhang, R. et al. Low-dose IL-2 therapy in autoimmune diseases: an update review. *Int. Rev. Immunol.* **43**, 113–137 (2024). 37882232 10.1080/08830185.2023.2274574

[506] Wang, J. et al. The numbers of peripheral regulatory T cells are reduced in patients with psoriatic arthritis and are restored by low-dose interleukin-2. *Ther. Adv. Chronic Dis.* **11**, 2040622320916014 (2020). 32523664 10.1177/2040622320916014 PMC7236566

[507] Amini, L., Kaeda, J., Weber, O. & Reinke, P. Low-dose interleukin-2 therapy: fine-tuning Treg in solid organ transplantation?. *Transplantation* **108**, 1492–1508 (2024). 38294829 10.1097/TP.0000000000004866 PMC11188637

[508] Bentebibel, S. E. et al. A first-in-human study and biomarker analysis of NKTR-214, a novel IL2Rbetagamma-biased cytokine, in patients with advanced or metastatic solid tumors. *Cancer Discov.* **9**, 711–721 (2019). 30988166 10.1158/2159-8290.CD-18-1495

[509] Sharma, M. et al. Bempegaldesleukin selectively depletes intratumoral Tregs and potentiates T cell-mediated cancer therapy. *Nat. Commun.* **11**, 661 (2020). 32005826 10.1038/s41467-020-14471-1 PMC6994577

[510] VanDyke, D. et al. Engineered human cytokine/antibody fusion proteins expand regulatory T cells and confer autoimmune disease protection. *Cell Rep.* **41**, 111478 (2022). 36261022 10.1016/j.celrep.2022.111478 PMC9631798

[511] de Picciotto, S. et al. Selective activation and expansion of regulatory T cells using lipid encapsulated mRNA encoding a long-acting IL-2 mutein. *Nat. Commun.* **13**, 3866 (2022). 35790728 10.1038/s41467-022-31130-9 PMC9256694

[512] Marshall, G. P. et al. Biomaterials-based nanoparticles conjugated to regulatory T cells provide a modular system for localized delivery of pharmacotherapeutic agents. *J. Biomed. Mater. Res. A* **111**, 185–197 (2023). 36082558 10.1002/jbm.a.37442 PMC9742177

[513] Kishimoto, T. K. et al. Rapamycin nanoparticles increase the therapeutic window of engineered interleukin-2 and drive expansion of antigen-specific regulatory T cells for protection against autoimmune disease. *J. Autoimmun.* **140**, 103125 (2023). 37844543 10.1016/j.jaut.2023.103125

[514] Oh, J. et al. The effect of the nanoparticle shape on T cell activation. *Small* **18**, e2107373 (2022). 35297179 10.1002/smll.202107373

[515] Prame Kumar, K., Ooi, J. D. & Goldberg, R. The interplay between the microbiota, diet and T regulatory cells in the preservation of the gut barrier in inflammatory bowel disease. *Front. Microbiol.* **14**, 1291724 (2023). 38107848 10.3389/fmicb.2023.1291724 PMC10722198

[516] Su, S. H. et al. Fecal microbiota transplantation and replenishment of short-chain fatty acids protect against chronic cerebral hypoperfusion-induced colonic dysfunction by regulating gut microbiota, differentiation of Th17 cells, and mitochondrial energy metabolism. *J. Neuroinflammation* **19**, 313 (2022). 36567333 10.1186/s12974-022-02675-9 PMC9791754

[517] Liu, H. et al. Gut microbiota from coronary artery disease patients contributes to vascular dysfunction in mice by regulating bile acid metabolism and immune activation. *J. Transl. Med.* **18**, 382 (2020). 33036625 10.1186/s12967-020-02539-x PMC7547479

[518] Hou, T. et al. IL-37 ameliorating allergic inflammation in atopic dermatitis through regulating microbiota and AMPK-mTOR signaling pathway-modulated autophagy mechanism. *Front. Immunol.* **11**, 752 (2020). 32411145 10.3389/fimmu.2020.00752 PMC7198885

[519] Britton, G. J. et al. Microbiotas from humans with inflammatory bowel disease alter the balance of gut Th17 and RORgammat(+) regulatory T cells and exacerbate colitis in mice. *Immunity* **50**, 212–224.e214 (2019). 30650377 10.1016/j.immuni.2018.12.015 PMC6512335

[520] Chen, P. & Tang, X. Gut microbiota as regulators of Th17/Treg balance in patients with myasthenia gravis. *Front. Immunol.* **12**, 803101 (2021). 35003133 10.3389/fimmu.2021.803101 PMC8732367

[521] Li, L. et al. Exploring the relationship between intestinal microbiota and immune checkpoint inhibitors in the treatment of non-small cell lung cancer: insights from the “lung and large intestine stand in exterior-interior relationship” theory. *Front. Cell. Infect. Microbiol.* **14**, 1341032 (2024). 38415012 10.3389/fcimb.2024.1341032 PMC10898591

[522] Matson, V. et al. The commensal microbiome is associated with anti-PD-1 efficacy in metastatic melanoma patients. *Science* **359**, 104–108 (2018). 29302014 10.1126/science.aao3290 PMC6707353

[523] Tanaka, A. & Sakaguchi, S. Regulatory T cells in cancer immunotherapy. *Cell Res.* **27**, 109–118 (2017). 27995907 10.1038/cr.2016.151 PMC5223231

[524] Michot, J. M. et al. Immune-related adverse events with immune checkpoint blockade: a comprehensive review. *Eur. J. Cancer* **54**, 139–148 (2016). 26765102 10.1016/j.ejca.2015.11.016

[525] Schnell, A. et al. Targeting PGLYRP1 promotes antitumor immunity while inhibiting autoimmune neuroinflammation. *Nat. Immunol.* **24**, 1908–1920 (2023). 37828379 10.1038/s41590-023-01645-4 PMC10864036

[526] Lai, Z. W. et al. Sirolimus in patients with clinically active systemic lupus erythematosus resistant to, or intolerant of, conventional medications: a single-arm, open-label, phase 1/2 trial. *Lancet* **391**, 1186–1196 (2018). 29551338 10.1016/S0140-6736(18)30485-9 PMC5891154

[527] Yan, J. J. et al. IL-2/anti-IL-2 complexes ameliorate lupus nephritis by expansion of CD4 CD25 Foxp3 regulatory T cells. *Kidney Int.* **91**, 603–615 (2017). 27914701 10.1016/j.kint.2016.09.022

[528] Proto, J. D. et al. Regulatory T cells promote macrophage efferocytosis during inflammation resolution. *Immunity* **49**, 666–667.e666 (2018). 30291029 10.1016/j.immuni.2018.07.015 PMC6192849

[529] Tanaka, A. & Sakaguchi, S. Targeting Treg cells in cancer immunotherapy. *Eur. J. Immunol.* **49**, 1140–1146 (2019). 31257581 10.1002/eji.201847659

[530] Hernandez, R., Poder, J., LaPorte, K. M. & Malek, T. R. Engineering IL-2 for immunotherapy of autoimmunity and cancer. *Nat. Rev. Immunol.* **22**, 614–628 (2022). 35217787 10.1038/s41577-022-00680-w

[531] Cinier, J. et al. Recruitment and expansion of Tregs cells in the tumor environment—how to target them? *Cancers* **13**, 1850 (2021). 10.3390/cancers13081850 PMC8069615 33924428

[532] Wang, H. P., Franco, F. & Ho, P. C. Metabolic regulation of Tregs in cancer: opportunities for immunotherapy. *Trends Cancer* **3**, 583–592 (2017). 28780935 10.1016/j.trecan.2017.06.005

[533] Liu, C. et al. Treg cells promote the SREBP1-dependent metabolic fitness of tumor-promoting macrophages via repression of CD8 T cell-derived interferon-γ. *Immunity* **51**, 381–397.e386 (2019). 31350177 10.1016/j.immuni.2019.06.017 PMC6703933

[534] Shitara, K. & Nishikawa, H. Regulatory T cells: a potential target in cancer immunotherapy. *Ann. N.Y. Acad. Sci.* **1417**, 104–115 (2018). 29566262 10.1111/nyas.13625

[535] Lainé, A. et al. Regulatory T cells promote cancer immune-escape through integrin αvβ8-mediated TGF-β activation. *Nat. Commun.* **12**, 6228 (2021). 34711823 10.1038/s41467-021-26352-2 PMC8553942

[536] Josefowicz, S. Z., Lu, L. F. & Rudensky, A. Y. Regulatory T cells: mechanisms of differentiation and function. *Annu. Rev. Immunol.* **30**, 531–564 (2012). 22224781 10.1146/annurev.immunol.25.022106.141623 PMC6066374

[537] Meyerson, H. J. et al. NRP-1/CD304 expression in acute leukemia: a potential marker for minimal residual disease detection in precursor B-cell acute lymphoblastic leukemia. *Am. J. Clin. Pathol.* **137**, 39–50 (2012). 22180477 10.1309/AJCP6VDBL4BRXRQA

[538] Adamczyk, M. et al. The expression of activation markers CD25 and CD69 increases during biologic treatment of psoriasis. *J. Clin. Med*. **12**, 6573 (2023). 10.3390/jcm12206573 PMC10607364 37892710

[539] Geoffroy, J. S. & Rosen, S. D. Demonstration that a lectin-like receptor (gp90MEL) directly mediates adhesion of lymphocytes to high endothelial venules of lymph nodes. *J. Cell Biol.* **109**, 2463–2469 (1989). 2681232 10.1083/jcb.109.5.2463 PMC2115886

[540] Schmaler, M. et al. IL-7R signaling in regulatory T cells maintains peripheral and allograft tolerance in mice. *Proc. Natl. Acad. Sci. USA* **112**, 13330–13335 (2015). 26450881 10.1073/pnas.1510045112 PMC4629352

[541] Wang, F. et al. Structures of mouse and human GITR-GITRL complexes reveal unique TNF superfamily interactions. *Nat. Commun.* **12**, 1378 (2021). 33654081 10.1038/s41467-021-21563-z PMC7925557

[542] Godfrey, J. et al. TIGIT is a key inhibitory checkpoint receptor in lymphoma. *J. Immunother. Cancer*. **11**, e006582 (2023). 10.1136/jitc-2022-006582 PMC10410806 37364933

[543] Chiang, E. Y. & Mellman, I. TIGIT-CD226-PVR axis: advancing immune checkpoint blockade for cancer immunotherapy. *J Immunother. Cancer*. **10**, 104–115 (2022). 10.1136/jitc-2022-004711 PMC8981293 35379739

[544] Zhang, Z., Guo, J. & Jia, R. Treg plasticity and human diseases. *Inflamm. Res.* **72**, 2181–2197 (2023). 37878023 10.1007/s00011-023-01808-x

